# The bidirectional immunoregulatory effects of Traditional Chinese Medicine on the cGAS-STING signaling pathway and their translational prospects

**DOI:** 10.1186/s13020-026-01494-x

**Published:** 2026-09-16

**Authors:** Yi Yang, Yiheng Sun, Yuxiang Cheng, Ziteng Pan, Jing Zhou

**Affiliations:** 1 School of Medical Imaging, Hangzhou Medical College, Hangzhou, 310053 Zhejiang China; 2 Department of Chemoradiotherapy, Ningbo No. 2 Hospital, Wenzhou Medical University, Ningbo, 315000 Zhejiang China; 3 Savaid Stomatology School, Hangzhou Medical College, Hangzhou, 311300 Zhejiang China; 4 School of Clinical Medicine, Hangzhou Medical College, Hangzhou, 310053 Zhejiang China

**Keywords:** cGAS-STING pathway, Traditional Chinese Medicine, Innate immunity, Inflammatory regulation, Tumor immunity, Autoimmune diseases

## Abstract

The cyclic GMP-AMP synthase–stimulator of interferon genes (cGAS-STING) pathway is a central component of innate immunity, mediating host defense against pathogens, antitumor responses, and inflammation. Aberrant activation of this pathway is implicated in a spectrum of diseases, including autoimmune disorders, chronic inflammation, organ injury, and metabolic dysregulation. Traditional Chinese Medicine (TCM), with its multi-component and multi-target characteristics, presents a promising strategy for modulating cGAS-STING signaling. Emerging evidence indicates that bioactive compounds, single herbs, herbal formulas, and related nanomedicines can bidirectionally regulate this pathway, either by directly targeting cGAS or STING or by influencing upstream processes such as DNA damage, mitochondrial dysfunction, and mitochondrial DNA (mtDNA) release. These interventions modulate inflammatory responses, immune cell activity, and disease progression. TCM does not regulate cGAS-STING signaling through a single pathway alone. The cGAS-STING pathway interacts with NF-κB signaling, NLRP3 inflammasome activation, MAPK pathways, oxidative stress, autophagy, ferroptosis, and pyroptosis. These interactions may help explain the multi-target and multi-pathway effects of TCM in immune regulation. Here, we review recent advances in understanding TCM-cGAS-STING interactions, highlighting their roles in infection, cancer, autoimmune disorders, and other complex diseases, and discuss the mechanistic insights and translational potential of TCM in immune modulation.

**Graphical Abstract:**

**Figure:**
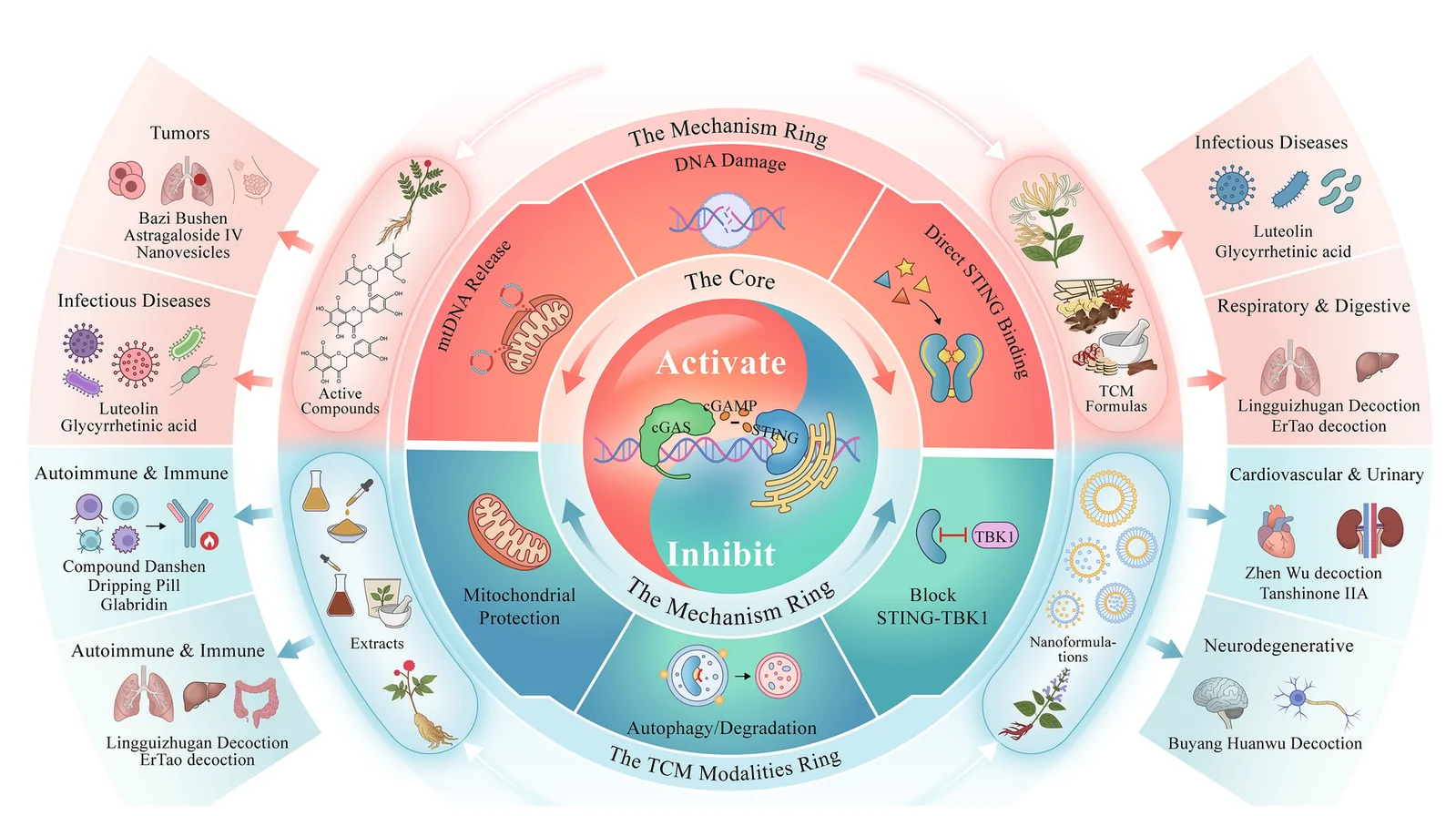

## Highlights

- Traditional Chinese Medicine bidirectionally regulates cGAS-STING signaling.
- Compounds, formulas, and nanoformulations target cGAS-STING at multiple levels.
- Mitochondrial damage and mtDNA release are common triggers of cGAS-STING activation.
- Target validation and clinical translation remain major barriers.

## Introduction

Traditional Chinese Medicine (TCM) has been practiced for millennia and represents a fundamental component of Chinese healthcare and cultural heritage. Chinese Herbal Medicine (CHM), a major branch of TCM, is derived from natural medicinal substances and includes single active compounds (such as astragaloside, curcumin, and quercetin), single-herb preparations, and multi-herb formulations [1]. CHM is characterized by its multi-component and multi-target properties, enabling modulation of multiple signaling pathways. Consequently, TCM has attracted increasing attention in chronic disease prevention, treatment, and sub-health management. The clinical efficacy of CHM has been widely validated, and elucidating its pharmacological mechanisms has become a key area of contemporary research [2]. Previous studies have shown that CHM modulates the cyclic GMP-AMP synthase–stimulator of interferon genes (cGAS-STING) signaling pathway, thereby influencing the progression of inflammatory and immune-related diseases, as well as tumors. For example, astragaloside can significantly increase the secretion of interferon-γ (IFN-γ) by T cells, thereby enhancing their immunological responsiveness [3]. Resveratrol reduces the expression levels of transcription factors associated with epithelial-mesenchymal transition (EMT), thereby inhibiting the invasion and migration of cancer cells [4]. These findings illustrate that CHM, through its ability to target multiple pathways, holds significant potential for the prevention and treatment of inflammation, immune dysregulation, and cancer.

Cyclic GMP-AMP synthase (cGAS) serves as a principal cytoplasmic DNA sensor in mammalian cells, regulating innate immune responses and mediating STING-dependent production of pro-inflammatory cytokines cytokines, including type I interferons (IFN-I) and interleukin-6 (IL-6). Beyond its immune functions, the cGAS-STING pathway contributes to cellular processes such as apoptosis, senescence, and autophagy, thereby maintaining cellular homeostasis. Activation of this pathway bridges innate and adaptive immunity, exerting bidirectional effects on tumorigenesis [5]. Specifically, STING signaling promotes dendritic cell maturation, enhances antigen presentation, facilitates T-cell activation and CD4⁺ differentiation, and induces CD8⁺ T-cell expansion with increased cytokine secretion. This orchestrates effector T-cell infiltration into tumors, synergizing with intrinsic IFN-I and chemokine signals to establish an antitumor immune microenvironment [6]. Conversely, sustained or excessive STING activation can paradoxically promote tumor progression through NF-κB and STAT3 signaling, as observed in contexts such as pancreatic ductal adenocarcinoma [7, 8]. Mechanistically, cGAS recognizes cytoplasmic double-stranded DNA (dsDNA) in a sequence-independent manner. Upon detection of abnormal dsDNA, cGAS catalyzes the formation of 2′,3′-cyclic GMP-AMP (2′,3′-cGAMP) from ATP and GTP. 2′,3′-cGAMP serves as a second messenger, binding to STING on the endoplasmic reticulum (ER), inducing STING oligomerization, and initiating its translocation to the Golgi apparatus via the ER–Golgi intermediate compartment(ERGIC). Activated STING recruits TANK-binding kinase 1(TBK1), which phosphorylates interferon regulatory factor 3(IRF3), while concurrently activating inhibitor of nuclear factor κB kinase(IKK) and NF-κB. Phosphorylated IRF3 and activated NF-κB translocate to the nucleus, promoting the transcription of downstream inflammatory mediators and IFN-Is, including TNF-α, IL-1β, IL-6, and IFN-I [9, 10].

**Fig. 1:**
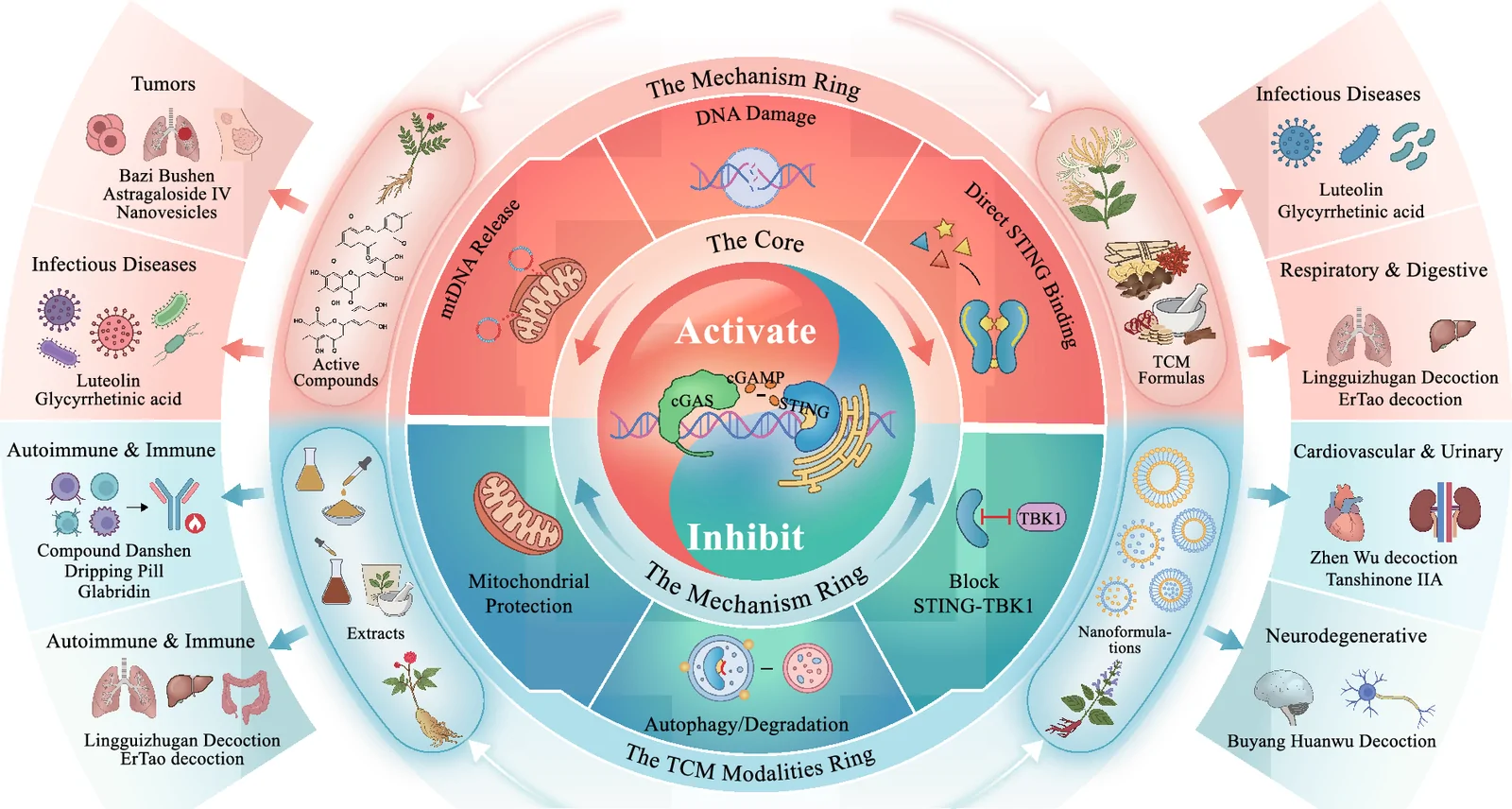
The various modes, molecular mechanisms and application scenarios of traditional Chinese medicine targeting the cGAS-STING pathway in treating diseases

In summary, the cGAS-STING signaling pathway functions as a central hub for cytoplasmic DNA recognition and innate immune responses, playing crucial roles in inflammation, immune regulation, and tumor immune surveillance. Dysregulation or persistent activation of this pathway is closely associated with the onset and progression of various diseases [11]. Recent evidence demonstrates that bioactive components of TCM, as well as single-herb and compound formulations, can modulate the cGAS-STING pathway and its downstream signaling molecules, such as TBK1, IRF3, and NF-κB, thereby influencing inflammatory factor release, immune cell activation, and cellular fate [12]. The multi-component, multi-target, and multi-pathway characteristics of TCM provide a mechanistic foundation for its use in anti-inflammatory, immunomodulatory, and antitumor applications, offering insights into potential strategies for disease prevention and therapeutic intervention (Fig. 1). From the perspective of TCM theory, this bidirectional modulation can be understood as a modern molecular interpretation of “strengthening healthy qi and eliminating pathogenic factors” and “balancing yin and yang.” Activation of cGAS-STING signaling in tumors and infections may enhance immune surveillance and pathogen or tumor clearance, corresponding to strengthening healthy qi and expelling pathogenic factors. Conversely, inhibition of excessive cGAS-STING activation in autoimmune diseases, chronic inflammation, and organ injury may help prevent immune-mediated damage and restore immune homeostasis, corresponding to balancing yin and yang.

## Introduction to the STING pathway: functions and clinical applications

### STING and the inflammatory response

**Fig. 2:**
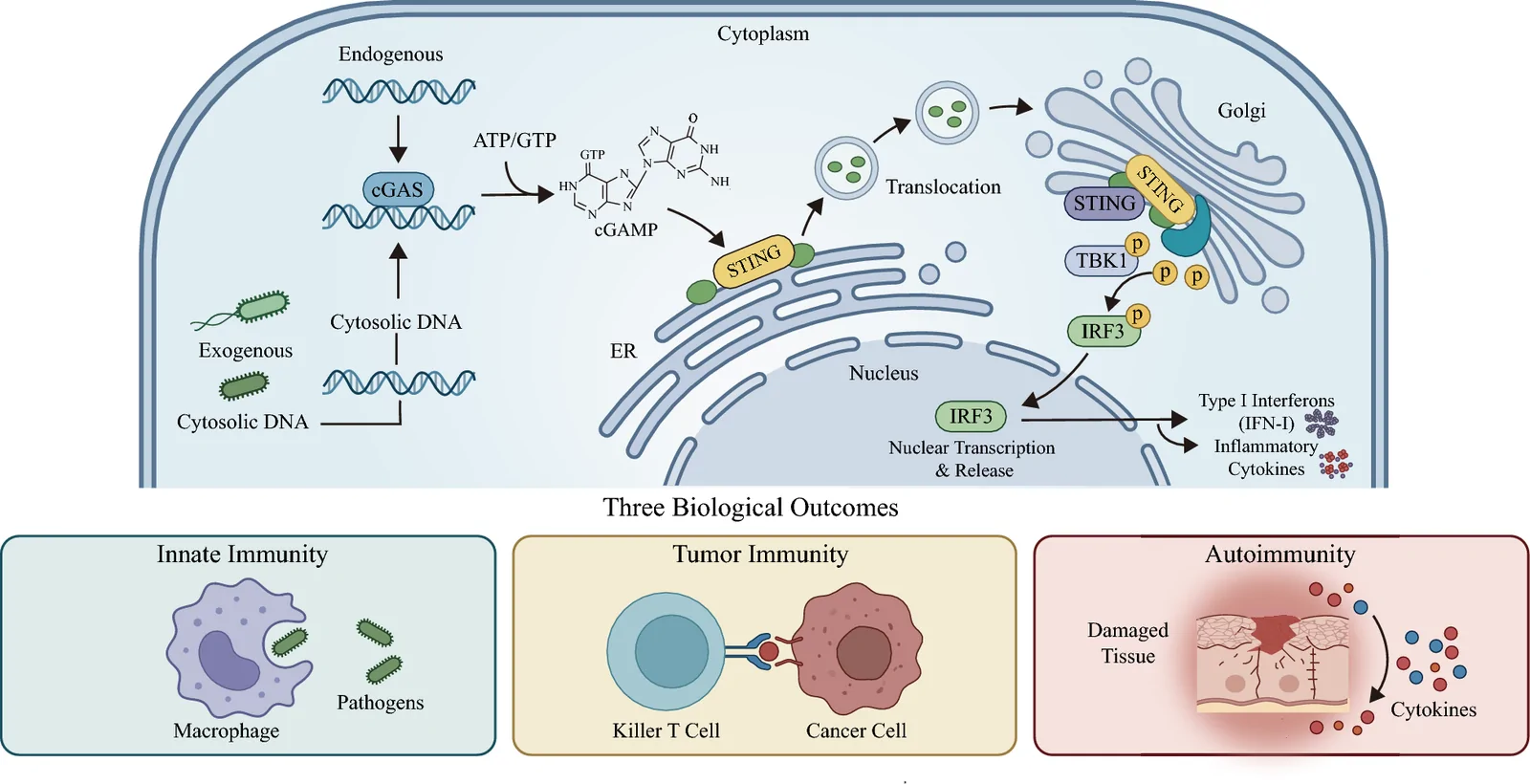
Canonical activation process of the cGAS–STING signaling pathway and its major biological effects

The cGAS-STING pathway amplifies inflammatory responses and orchestrates the innate immune defense. Dysregulation of this pathway is recognized as a central mechanism underlying multiple inflammatory disorders. STING-dependent inflammatory responses are crucial for maintaining homeostasis; moderate activation helps rapidly restore immune balance. In contrast, excessive or sustained activation leads to abnormal secretion of proinflammatory cytokines, particularly IFN-Is (IFN-I), potentially precipitating autoimmune diseases such as Aicardi–Goutières syndrome and systemic lupus erythematosus. These pathological effects are primarily mediated through two mechanisms: first, accumulation of intracellular DNA, which persistently activates the cGAS-STING axis and induces overproduction of inflammatory mediators; second, gain-of-function mutations in STING, resulting in constitutive activation independent of upstream signaling, ultimately driving autoimmune pathology [13]. Accordingly, precise modulation of the intensity and duration of STING signaling represents a promising strategy for maintaining immune homeostasis, preventing aberrant inflammatory responses, and developing targeted anti-inflammatory interventions (Fig. 2).

This figure illustrates the basic activation process of the cGAS–STING signaling pathway. Cytosolic DNA, derived either from exogenous pathogenic nucleic acids or endogenous abnormally leaked DNA, is recognized by the cytosolic DNA sensor cGAS, which catalyzes the synthesis of the second messenger cGAMP from ATP and GTP. Binding of cGAMP to STING on the endoplasmic reticulum triggers STING activation and promotes its translocation from the endoplasmic reticulum to the Golgi apparatus, where it recruits and activates TBK1. Activated TBK1 subsequently mediates IRF3 phosphorylation and nuclear translocation, ultimately inducing the expression of type I interferons and pro-inflammatory cytokines. As a central hub linking cytosolic DNA sensing to immune responses, the cGAS–STING pathway is broadly involved in innate immune defense, tumor immunosurveillance, and autoimmune/inflammatory responses. Moderate activation of this pathway contributes to anti-infective and antitumor immunity, whereas persistent or aberrant activation may drive chronic inflammation and autoimmune tissue injury.

### STING and the immune system

Innate immunity constitutes the first line of defense against invading pathogens, enabling rapid recognition and clearance of foreign microorganisms. The STING pathway functions as a critical hub within innate immunity, sensing cytoplasmic DNA and inducing rapid expression of IFN-I and other inflammatory mediators, thereby mobilizing host defenses [14, 15]. Moreover, STING modulates neutrophil activity via interferon-stimulated gene (ISGs) induction, influencing the efficacy of immunotherapeutic interventions [16]. Excessive activation, however, may promote autoimmune or inflammatory diseases. For instance, during SARS-CoV-2 infection, hyperactivation of the cGAS-STING pathway leads to elevated IFN-I production and immunopathological consequences, underscoring the necessity of balancing immune protection with potential inflammatory risk [17].

Beyond innate immunity, STING plays a pivotal role in adaptive immune regulation. By modulating antigen presentation and cytokine secretion, STING influences T and B cell activation, contributing to immune homeostasis [13, 14]. Studies have shown that small-molecule inhibitors, such as C-176 and C-178, can target and inhibit STING, providing a novel approach to modulating the immune system in conditions of excessive immune activation. These inhibitors have been explored as potential therapies for autoimmune and inflammatory diseases, where STING-driven inflammation may be contributing to disease pathogenesis [18]. However, the role of STING in autoimmune diseases is complex and dual-natured. On one hand, activation of the STING signaling pathway is essential for enhancing immune responses against pathogens and tumors. On the other hand, dysregulated or chronic STING activation can drive the development of autoimmune diseases, as seen in conditions such as systemic lupus erythematosus (SLE) and Aicardi–Goutières syndrome [18]. In experimental models, it has been demonstrated that excessive STING activation can induce the generation of regulatory B cells, which in turn suppress natural killer (NK) cell activity. This suppression of NK cell function weakens the body’s ability to surveil and eliminate malignant cells, increasing the risk of autoimmune responses [19]. Furthermore, sustained activation of the STING pathway promotes the overproduction of IFN-I, which fuels chronic inflammation and exacerbates autoimmune conditions. For example, in the case of SLE, excessive STING activation contributes to the persistent production of inflammatory cytokines, which accelerates disease progression [20]. These findings suggest that while STING signaling is protective in response to pathogens, its excessive or prolonged activation can have detrimental effects, potentially triggering autoimmune disease. Therefore, the precise regulation of STING activation is critical for balancing effective immune responses with the prevention of immune pathology. Strategies to modulate STING signaling in a controlled manner may hold significant therapeutic potential for a variety of autoimmune and inflammatory diseases.

### STING and tumor immunology

STING contributes to tumor immune surveillance by detecting cytoplasmic DNA released from malignant cells. DNA fragments derived from proliferating or dying tumor cells activate the cGAS-STING pathway, inducing IFN-I and pro-inflammatory cytokine production, thereby enhancing antitumor immunity [21]. STING agonists stimulate dendritic cell activation and NK cell recruitment, improving the efficiency of antitumor immune responses [16]. Additionally, STING signaling can suppress the reactivation of dormant metastatic foci, delaying tumor recurrence [21], underscoring its dual role in initiating immune responses and regulating tumor progression.

Tumor cells may, however, exploit STING signaling to evade immune surveillance. For example, STING-induced regulatory B cells can inhibit NK cell activity, weakening antitumor immunity [19]. Conversely, three prime repair exonuclease 1(TREX1) inhibition elevates cytoplasmic DNA accumulation, enhancing STING-dependent interferon signaling and bolstering antitumor responses [22]. This illustrates that STING signaling in tumor immunity is subject to complex bidirectional regulation, necessitating precise control to achieve optimal therapeutic outcomes. 

Despite its potential, STING-targeted cancer therapies face challenges, including systemic inflammation, dose-dependent toxicity, and limited delivery efficiency [23, 24]. Consequently, the development of STING-targeted therapies requires an integrated approach combining rational drug design, advanced delivery technologies, and strategic combination treatments to achieve safer, more effective, and personalized cancer immunotherapy.

### Crosstalk between cGAS-STING and other inflammatory pathways

The cGAS-STING pathway acts as a signaling hub in inflammatory regulation. This pathway does not work alone. After activation, STING recruits TBK1 and promotes IRF3 phosphorylation [25]. This process induces type I interferon production [26]. STING can also activate NF-κB signaling. NF-κB then promotes the expression of inflammatory cytokines, including TNF-α, IL-6, and IL-1β [12, 25].

The cGAS-STING pathway also interacts with the NLRP3 inflammasome. This interaction can promote caspase-1 activation, IL-1β maturation, and pyroptosis. Mitochondrial damage can further amplify this process. Damaged mitochondria release mitochondrial DNA and reactive oxygen species. These signals can activate cGAS-STING, NLRP3, and MAPK-related inflammatory responses [27, 28].

Autophagy also regulates cGAS-STING signaling. Autophagy can remove damaged mitochondria and cytosolic DNA [29]. This process may limit persistent STING activation and reduce excessive inflammation. In contrast, impaired autophagy may increase cytosolic DNA accumulation and strengthen cGAS-STING activation [29].

The cGAS-STING pathway is also linked to regulated cell death. STING activation may promote pyroptosis, ferroptosis, apoptosis, and senescence under different pathological conditions [27, 30, 31]. These forms of cell death can release additional danger signals. These signals may further enhance inflammatory responses. Therefore, cGAS-STING should be regarded as a central node. This node connects DNA sensing, interferon responses, NF-κB-mediated inflammation, inflammasome activation, oxidative stress, MAPK signaling, autophagy, and regulated cell death.

In summary, STING plays a crucial role in antitumor immunity by enhancing immune surveillance and modulating responses through complex feedback mechanisms. Activation of the cGAS-STING pathway stimulates IFN-Is and proinflammatory cytokines, promoting the activation of dendritic cells, natural killer cells, and cytotoxic T lymphocytes. Precise regulation of STING signaling is essential, as excessive activation may cause immune suppression or chronic inflammation, while optimal modulation can strengthen antitumor responses and improve the efficacy of immunotherapies. Future research should focus on identifying strategies to regulate STING activity across different tumor types, microenvironments, and treatment regimens, thereby guiding clinical translation and enabling more effective, personalized cancer therapies.

## The role of Traditional Chinese Medicine in immune regulation

In TCM theory, disease progression is closely related to the dynamic imbalance between healthy qi and pathogenic factors, as well as disruption of yin-yang homeostasis. Therefore, therapeutic principles such as strengthening healthy qi, eliminating pathogenic factors, clearing heat and toxins, resolving blood stasis, and tonifying deficiency while reducing excess are central to TCM intervention. In modern immunological terms, these concepts are closely associated with the maintenance of immune homeostasis. Depending on the pathological context, TCM may either activate or inhibit cGAS-STING signaling. When immune surveillance is insufficient or immune escape predominates, activation of cGAS–STING signaling may enhance antitumor or anti-pathogen immune responses. Conversely, when persistent or aberrant cGAS-STING activation drives chronic inflammation, tumor-promoting immune dysregulation, autoimmune responses, or tissue injury, TCM-mediated inhibition of this pathway may help limit pathological inflammation and prevent immune-mediated damage. Thus, the bidirectional regulation of the cGAS-STING pathway by TCM provides a molecular basis for understanding the classical TCM concept of restoring systemic balance [32, 33].

Research indicates that the regulation of the cGAS-STING pathway by TCM is not confined to a single disease or organ. Instead, it manifests as an intervention in common pathological processes such as inflammation, oxidative stress, and tissue damage. For example, active components from Ligustri Lucidi Fructus (LLF) inhibit cGAS-STING signaling and modulate the NF-κB inflammatory pathway, thereby alleviating inflammation and damage in renal tubular cells induced by high glucose and fatty acids [34]. Similar studies suggest that natural products often act on multiple targets, forming a multi-target synergistic immune regulatory network by interacting with cGAS-STING, NF-κB, MAPK, and oxidative stress-related pathways [3, 33]. These findings also reflect the correspondence between TCM herbal properties and modern immune mechanisms. For example, tonic herbs are often associated with strengthening healthy qi and supporting host defense, heat-clearing and detoxifying herbs with limiting excessive inflammatory responses, and blood-activating herbs with improving inflammatory tissue injury and microenvironmental remodeling.

This characteristic is prominent in studies of TCM formulas. Linggui Zhugan Decoction reduces inflammatory factor expression by inhibiting the STING-TBK1-NF-κB signaling axis in macrophages, improving high-fat diet-induced hepatic lipid accumulation and oxidative stress responses [35]. Linggui Zhugan Decoction provides another example of how the monarch-minister-assistant-guide structure may be linked to multilevel regulation of cGAS-STING-related inflammation. In the traditional compatibility structure of this formula, Poria serves as the monarch herb, Cinnamomi Ramulus as the minister herb, Atractylodis Macrocephalae Rhizoma as the assistant herb, and Glycyrrhizae Radix et Rhizoma Praeparata cum Melle as the guide and harmonizing herb. Poria-derived triterpenoids and polysaccharides may provide the principal anti-inflammatory and metabolic regulatory effects, whereas constituents derived from Cinnamomi Ramulus may complement these effects by regulating circulation, oxidative stress, and inflammatory signaling. Atractylodis Macrocephalae Rhizoma may further support mitochondrial and metabolic homeostasis, thereby reducing upstream signals such as ROS accumulation and mtDNA leakage that can drive cGAS-STING activation. Glycyrrhiza-derived constituents may modulate downstream cytokine responses, harmonize the pharmacological actions of the other herbs, and potentially limit excessive inflammatory or adverse effects.

This compatibility pattern suggests that the inhibition of STING-TBK1-NF-κB signaling by Linggui Zhugan Decoction may not result from a single herb acting directly on STING. Instead, it may reflect coordinated regulation of upstream mitochondrial and metabolic stress, the core STING signaling cascade, and downstream inflammatory responses. However, because the available study evaluated the complete decoction, the individual contributions of the four herbs and their representative constituents have not been confirmed. Moreover, findings obtained from isolated constituents cannot be directly extrapolated to their effects within the complete formula, because extraction, dosage ratios, metabolism, and herb-erb interactions may alter both pathway activity and biological outcome.

The Wen-Shen-Tong-Luo-Zhi-Tong Decoction targets the intertwined pathological processes of inflammation, senescence, and tissue repair. By inhibiting LONP1-mediated cGAS-STING activation, it alleviates the senescence of bone marrow mesenchymal stem cells and promotes bone formation [36]. Although these studies involve different tissues, such as kidneys, liver, and bone, they share a common mechanism: TCM alleviates tissue damage caused by immune imbalance through regulation of the cGAS-STING pathway and related inflammatory networks.

Overall, leveraging its multi-component, multi-target, and multi-pathway characteristics, TCM not only directly modulates the cGAS-STING signaling pathway but also synergistically influences processes related to NF-κB, MAPK, oxidative stress, and cellular senescence. This provides immunomodulatory and tissue-protective effects across various disease contexts [3, 33]. Researchers also emphasize that natural plant products targeting digestive system diseases can improve the immune environment in conditions like inflammatory bowel disease, fatty liver, and gastrointestinal tumors by regulating the cGAS-STING pathway, providing a molecular basis for TCM interventions in digestive diseases [33].

In summary, Traditional Chinese Medicine modulates the cGAS-STING signaling pathway through its multi-component, multi-target mechanisms, enhancing disease resistance and antitumor activity while suppressing excessive inflammation and autoimmune responses. This offers a scientific basis for immunological interventions in inflammatory, metabolic, and autoimmune diseases as well as cancer, and lays the groundwork for the modernization of traditional Chinese medicine and the development of precision immunotherapy.

## The relationship between the STING pathway and Traditional Chinese Medicine

In recent years, the cGAS-STING signaling pathway has emerged as a central hub for the detection of cytoplasmic DNA and the activation of innate immunity, playing critical roles in infection defense, inflammation regulation, and tumor immunity [26, 37]. Upon recognition of cytoplasmic DNA by cGAS, the second messenger cGAMP is generated, which subsequently activates STING and its downstream TBK1-IRF3 and NF-κB signaling pathways. This activation induces the expression of IFN-Is (IFN-I) and various inflammatory mediators, thereby reshaping the immune landscape and influencing disease progression [11, 25, 38]. The STING pathway exhibits dual effects: moderate activation supports host anti-infectious and antitumor immunity, whereas sustained or aberrant activation may drive chronic inflammation, immune dysregulation, and tissue damage [12, 39]. Recent studies indicate that TCM and natural bioactive compounds can modulate cGAS-STING signaling by regulating processes such as the DNA damage response, mitochondrial homeostasis, oxidative stress, and autophagy, highlighting their potential for bidirectional modulation of this pathway [12, 31]. Therefore, reviewing TCM’s regulation of the STING pathway from both activation and inhibition perspectives is essential for elucidating its pharmacological basis and clinical translational value. The cGAS-STING pathway is closely connected with NF-κB, NLRP3 inflammasome, MAPK, oxidative stress, autophagy, ferroptosis, pyroptosis, apoptosis, and senescence. Therefore, TCM may regulate immune responses by acting on several related pathways at the same time. However, the studies summarized in this section differ substantially in evidence strength. Therefore, we distinguished direct target validation, indirect pathway modulation, and prediction-based analyses when discussing these studies.

To avoid an oversimplified pathway-level description, the mechanisms by which TCM-derived interventions regulate cGAS-STING signaling can be further classified according to their molecular modes of action. These mechanisms include direct binding to cGAS or STING, covalent modification of specific residues, regulation of STING oligomerization and intracellular trafficking, promotion of STING degradation, disruption of STING-TBK1 or STING-IRF3 interactions, and indirect modulation through upstream danger signals such as DNA damage, mitochondrial dysfunction, mtDNA leakage, ROS accumulation, mitophagy, chromatin fragment formation, and nuclear DNA release. In addition, nanomedicine-based systems may regulate this pathway through delivery-dependent and metal-ion-assisted mechanisms. This classification helps distinguish direct target engagement from indirect pathway modulation and provides a clearer framework for understanding the bidirectional regulation of cGAS-STING signaling by TCM.

Aberrant cytosolic double-stranded DNA (dsDNA) may originate from pathogen infection, such as HSV-1 and SARS-CoV-2, leakage of damaged nuclear DNA, or release of mitochondrial DNA (mtDNA). Upon sensing cytosolic dsDNA, cGAS catalyzes the synthesis of 2′,3′-cGAMP from ATP and GTP. cGAMP then binds to STING on the endoplasmic reticulum and induces its activation, followed by STING translocation to the Golgi apparatus and recruitment of TBK1, which subsequently activates IRF3 and NF-κB, ultimately leading to the expression of type I interferons, chemokines, and pro-inflammatory cytokines. Some bioactive compounds from traditional Chinese medicine enhance cGAS-STING signaling by promoting the leakage of damaged nuclear DNA, increasing mitochondrial reactive oxygen species production, and facilitating mtDNA release, thereby promoting CD8^+^ T-cell infiltration, inducing M1 macrophage polarization, and enhancing tumor immunogenicity. These effects have been implicated in lung cancer, breast cancer, colorectal cancer, and viral infection. Representative compounds include honokiol (HNK), Astragaloside IV, tetrandrine (TET), and cucurbitacin B (Cuc B). In contrast, other bioactive compounds suppress cGAS-STING signaling by alleviating mitochondrial damage, reducing mtDNA leakage, inhibiting STING trafficking, or promoting autophagy–lysosomal degradation, thereby attenuating oxidative stress and excessive inflammatory responses and facilitating tissue repair. Such protective effects have been reported in acute lung, liver, and kidney injury, cardiovascular diseases, neuroinflammation, and organ fibrosis. Representative compounds include naringenin, curcumol, and licochalcone A (Lico A).

### Traditional Chinese Medicine activates the STING pathway

**Fig. 3:**
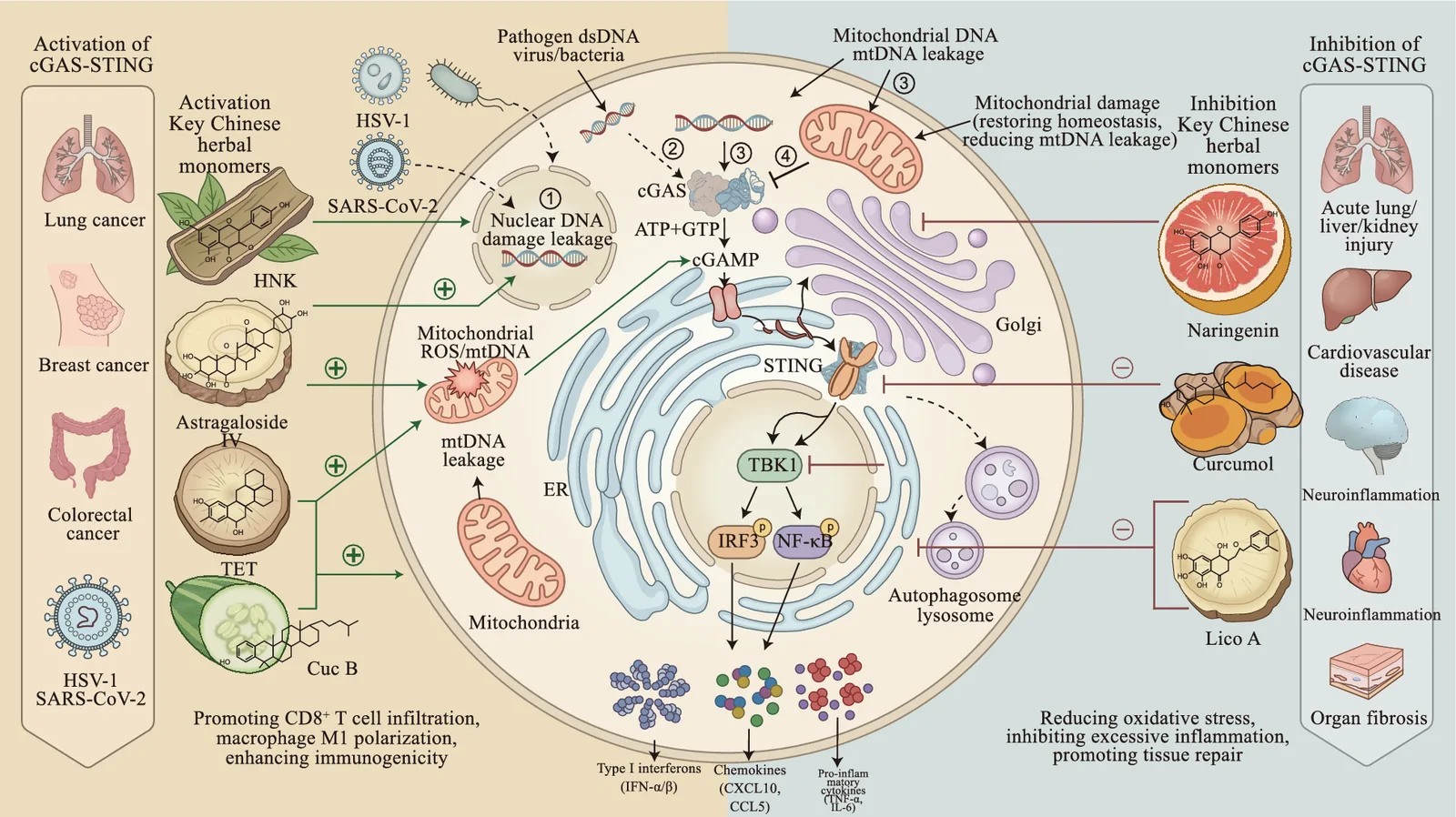
Bidirectional regulation of the cGAS–STING signaling pathway by bioactive compounds from traditional Chinese medicine and their roles in tumors, viral infection, and inflammatory injury

Activation of the STING pathway is a key mechanism for enhancing innate immune recognition and antitumor immunity [11, 25, 38]. In pathological contexts characterized by insufficient immune surveillance or immune escape, such immunostimulatory effects are consistent with the TCM principle of strengthening healthy qi and eliminating pathogenic factors. In cancer, STING activation promotes dendritic cell maturation and antigen presentation, enhances CD8⁺ T cell recruitment and effector function, and thereby improves the tumor immune microenvironment, increasing sensitivity to antitumor therapies [40–42]. Certain naturally derived compounds and active components of TCM can indirectly activate the cGAS–STING pathway by promoting cytoplasmic DNA accumulation, inducing mitochondrial DNA release, or enhancing DNA damage-associated immune signaling, thus exerting immunostimulatory and antitumor effects [43]. Therefore, STING pathway activation represents a critical mechanism through which TCM mobilizes the body’s immune response and intervenes in tumor- and pathogen-related diseases (Fig. 3).

Mechanistically, TCM-mediated activation of the cGAS-STING pathway can be divided into several distinct modes. The first mode involves direct engagement of cGAS or STING. For example, luteolin directly binds the active site of cGAS and enhances cGAS oligomerization, whereas glycyrrhetinic acid and magnolol have been reported to directly bind STING and promote downstream TBK1-IRF3 activation. The second mode involves DNA damage-mediated activation, in which compounds such as paeoniflorin, plumbagin, and cucurbitacin B increase cytosolic dsDNA accumulation and subsequently activate cGAS-dependent signaling. The third mode is mitochondrial stress-mediated activation, characterized by mitochondrial dysfunction, ROS accumulation, and mtDNA leakage, as observed with isoliensinine, cucurbitacin B, dihydroartemisinin, brazilin, and astragaloside IV. The fourth mode involves enhancement of STING stability or signalosome assembly, such as atractylenolide-mediated inhibition of STING lysosomal degradation and schisandrin C-mediated promotion of STING-TBK1 interaction. In nanomedicine-based systems, metal ions such as Mn^2+^ may further amplify DNA sensing and STING activation, generating a delivery system-dependent mode of immunostimulation. These differentiated mechanisms indicate that TCM-derived activators do not merely converge on STING/TBK1/IRF3 phosphorylation but regulate the pathway through multiple upstream and molecularly distinct processes.

#### Tumor diseases

In recent years, an increasing body of research has demonstrated that various bioactive components in TCM can activate the cGAS–STING signaling pathway. This can occur either by directly acting on STING or by inducing DNA damage, mitochondrial dysfunction, and mitochondrial DNA (mtDNA) release. As a result, these components exert antitumor, anti-metastatic, and immunotherapy-sensitizing effects in various cancers, including lung cancer, breast cancer, colorectal cancer, osteosarcoma, and T-cell malignancies. From a TCM perspective, these antitumor effects can be partly interpreted through the principles of strengthening healthy qi, eliminating toxins, resolving masses, and dispersing stasis, especially when STING activation enhances immune surveillance and reverses tumor immune escape.

From a mechanistic perspective, some active components of TCM can directly activate STING and initiate downstream immune responses. Magnolol, for instance, directly binds to key sites on STING, activating the STING/TBK1/IRF3 pathway. This induces the expression of IFN-Is and chemokines, promotes CD8⁺ T-cell infiltration and activation in melanoma, colorectal cancer, and breast cancer models, and enhances the efficacy of anti-PD-(L)1 immunotherapy [44]. Vanillic acid also directly activates STING, particularly in macrophages, promoting the phosphorylation and nuclear translocation of STING/TBK1/IRF3, inducing the expression of IFN-β and CXCL10. This, in turn, promotes the polarization of macrophages toward the M1 phenotype, enhancing their phagocytic capacity and ability to induce tumor cell apoptosis [45]. Astragaloside IV not only binds to and activates STING stably but is also associated with mitochondrial dysfunction and mtDNA release, suggesting it plays a dual role in directly activating and amplifying the STING signaling pathway [46]. Since Astragalus membranaceus is traditionally regarded as a qi-tonifying herb, this mechanism may provide a molecular explanation for the TCM concept of strengthening healthy qi to enhance immune surveillance.

In addition to direct activation, the activation of the STING pathway mediated by DNA damage has garnered considerable attention. In non-small cell lung cancer (NSCLC), paeoniflorin induces DNA damage and promotes the accumulation of cytoplasmic dsDNA, which subsequently activates the STING/TBK1/IRF3 pathway. This upregulates CCL5 and CXCL10 expression, thereby enhancing the infiltration of dendritic cells, macrophages, and CD8⁺ T cells, thus improving the efficacy of anti-PD-1 immunotherapy [47]. Plumbagin also induces DNA damage and cytoplasmic dsDNA accumulation via ROS, activates the STING pathway, and inhibits tumor cell proliferation while inducing apoptosis [48]. In osteosarcoma, cucurbitacin B induces DNA damage responses by increasing γ-H2AX levels, further activating the cGAS-STING pathway, promoting IFN-γ and CCL5 expression, and increasing CD4⁺ and CD8⁺ T-cell infiltration within the tumor while reducing regulatory T cells (Tregs) and Myeloid-Derived Suppressor Cell (MDSC). This illustrates that such compounds not only directly kill tumor cells but also improve the tumor microenvironment through immune activation [49].

Mitochondrial damage and mtDNA release are among the most common upstream activating events. In T-cell malignancies, isoliensinine induces ROS-dependent mitochondrial damage by inhibiting PRDX1 and suppressing PINK1/Parkin-mediated mitochondrial autophagy, leading to the accumulation of mtDNA in the cytoplasm. This activates the cGAS-STING pathway, enhancing the immunogenicity of tumor cells and increasing NK cell clearance capacity [50]. In malignant breast cancer, cucurbitacin B targets MTCH2 to induce mitochondrial fragmentation and elevated ROS levels, promoting mtDNA release and activating the cGAS-STING pathway. This, in turn, promotes antitumor neutrophil differentiation and CD8⁺ T-cell activation [51]. Dihydroartemisinin disrupts mitochondrial homeostasis by inhibiting TOM70, leading to mtDNA damage and release, which activates the cGAS/STING/NLRP3 pathway, induces pyroptosis, and enhances tumor immunogenicity [52]. Furthermore, compounds such as brazilin, colchicine, rocaglamide, and astragaloside IV activate the STING pathway through mitochondrial dysfunction, ROS production, or mtDNA release. Hence, mitochondrial stress may serve as a common upstream mechanism by which TCM modulates STING signaling [46, 53].

It is noteworthy that the activation of the STING pathway by TCM not only enhances effector immune responses but also alleviates tumor-induced immunosuppression. In NSCLC, Brazilin downregulates CXCL1 expression by activating the STING/TBK1/IRF3 pathway, inhibiting MDSC recruitment, reducing Treg infiltration, and increasing CD8⁺ T-cell infiltration. This reshapes the local tumor microenvironment, making it more conducive to antitumor immunity [53]. On the other hand, vanillic acid promotes the polarization of macrophages toward the M1 phenotype, suggesting that STING activation not only targets tumor cells but also enhances the overall antitumor immune response by regulating myeloid cell function [45]. Moreover, rutecarpine upregulates chemokine expression, reduces PD-L1 expression, and increases CD8⁺ T-cell infiltration in NSCLC by activating the STING/TBK1/IRF3 pathway, further underscoring the potential value of TCM in modulating the tumor immune microenvironment [54].

Additionally, the potential of TCM to activate the STING pathway in combination therapy is garnering increasing attention. In a lung cancer metastasis model, salvianolic acid B acts as a Mn^2^⁺ ion carrier, promoting its uptake and retention in lung tissue while directly activating STING. This synergistically enhances the STING/TBK1/IRF3 pathway with Mn^2^⁺, promoting the elimination of tumor cells by CD8⁺ T cells and macrophages, while simultaneously mitigating Mn^2^⁺-induced inflammatory responses [55]. As a representative component of Salvia miltiorrhiza, salvianolic acid B also reflects the traditional function of activating blood circulation and resolving stasis, which may be linked to improved immune-cell infiltration and remodeling of the tumor microenvironment. Compounds such as paeoniflorin, magnolol, and cucurbitacin B can also enhance the efficacy of anti-PD-1 or anti-PD-L1 therapies [56]. Astragaloside IV increases the sensitivity of cisplatin in treating triple-negative breast cancer while reducing its toxic side effects [46]. Ginsenoside Rh2 synergizes with IFN-γ to activate the STING/TBK1/IRF3 pathway, enhancing interferon signaling and promoting apoptosis in colorectal cancer cells [57]. These studies suggest that TCM activation of the STING pathway not only exerts direct antitumor effects but also holds promise as a key sensitization strategy for immunotherapy and chemotherapy. 

In summary, bioactive components of TCM can activate the STING pathway through multiple mechanisms, including direct binding to STING, induction of DNA damage, disruption of mitochondrial homeostasis, and promotion of mtDNA release. This activation enhances the immunogenicity of tumor cells by inducing the expression of IFN-Is and chemokines, promoting the infiltration of effector immune cells, and alleviating immunosuppression, thereby exerting antitumor effects. Although this field shows promising prospects, the relevant evidence primarily stems from cellular and animal studies. Future research should focus on further validating target mechanisms, conducting safety evaluations, and advancing clinical translation studies to facilitate the application of TCM targeting the STING pathway in cancer therapy. The strength of evidence also differs among these antitumor studies. Studies involving direct STING binding, pathway rescue, or pharmacological validation provide stronger mechanistic support. In contrast, studies that only report changes in STING-related proteins, immune-cell infiltration, or cytokine expression provide indirect pathway-associated evidence. These differences should be considered when interpreting the antitumor potential of TCM targeting cGAS-STING signaling.

#### Viral infections

Beyond oncology, antiviral immunity represents another important therapeutic context in which TCM-mediated STING activation has attracted increasing attention. In viral infections, activation of the cGAS-STING pathway primarily enhances host innate immune recognition and promotes type I interferon-mediated antiviral defense.

In viral infections, various TCM components enhance STING-related innate immune responses through multiple mechanisms, thereby inhibiting viral replication and alleviating tissue damage. This is consistent with the TCM principle of clearing heat and toxins and eliminating pathogenic factors, in which appropriate immune activation contributes to the clearance of invading pathogens. Some components act directly on cGAS or STING. In an HSV-1 infection model, luteolin binds directly to the active site of cGAS, promoting its oligomerization, activating antiviral signaling, and upregulating IFN-β and interferon-stimulated gene expression. This ultimately inhibits viral replication and mitigates neurological damage associated with viral encephalitis [58]. Glycyrrhetinic acid binds key residues on STING, promoting TBK1 and IRF3 activation and nuclear translocation, upregulating IFN-I expression, and exhibiting significant antiviral and pulmonary protective effects in SARS-CoV-2 Omicron infection models [59]. Beyond direct activation, some compounds amplify antiviral responses by stabilizing STING or enhancing signal transduction. Atractylenolide inhibits lysosomal degradation of STING by targeting the cholesterol transporter NPC1, enhancing STING-dependent innate immunity and suppressing replication of viruses including VSV and H1N1 [60]. Schisandrin C promotes STING-TBK1 interaction, enhancing STING activation and upregulating IFN-β and interferon-stimulated gene expression, thereby reducing HBsAg levels in HBV infection models [61]. Corilagin similarly strengthens STING signaling, mitigating liver and lung tissue pathology caused by cytomegalovirus infection [62]. Some TCM compounds act via STING-mediated mechanisms beyond the cGAS pathway. Cepharanthine promotes autophagosome maturation through STING/TBK1/p62 signaling, enhancing viral clearance and inhibiting HSV-1 replication [63]. The Euphorbia lathyris compound Dpo activates host innate immunity via STING, and through the synergistic action of IRF3, IRF7, and ELF4, induces interferon-stimulated genes and chemokines, demonstrating inhibitory effects against VSV and HSV-1 in vitro and in vivo [64].

Recent studies have further expanded STING-targeted antiviral research from isolated natural compounds to traditional Chinese medicines and classical herbal formulations, indicating a transition from single-compound exploration toward integrated regulation of antiviral immunity. Ginsenoside Rg3, a representative bioactive constituent derived from Panax ginseng, enhances resistance to enteric viral infection by enriching short-chain fatty acid-producing commensal bacteria, thereby activating the cGAS-STING/type I interferon axis and strengthening antiviral innate immunity [65]. In addition, Sophora tonkinensis, a traditional medicinal herb, suppresses HBV replication by enhancing cGAS-STING activation and downstream interferon signaling [66]. Moreover, the topical herbal formulation JieZe-1 alleviates HSV-2 and Candida albicans co-infection through activation of the cGAS/STING/IFN-I pathway, highlighting the therapeutic potential of multi-component TCM formulas in antiviral immune regulation [67]. Collectively, TCM-mediated STING activation in antiviral defense involves not only interferon production but also STING stabilization, autophagic clearance, and transcriptional networks.

#### Fibrotic diseases

In fibrotic diseases, STING signaling exhibits a highly context-dependent function distinct from its classical role in immune activation. Although persistent STING activation may contribute to chronic inflammation and tissue injury, emerging evidence indicates that controlled activation of this pathway can also promote resolution of pathological remodeling. In these diseases, the therapeutic relevance of traditional Chinese medicine in activating the STING pathway extends beyond merely enhancing inflammation; it involves inducing pathological cellular senescence, promoting immune-mediated clearance, and inhibiting aberrant extracellular matrix deposition. Ligustilide, for instance, disrupts cytoskeletal and nuclear membrane homeostasis, leading to the leakage of nuclear double-stranded DNA into the cytoplasm. This triggers the cGAS-STING pathway, promotes hepatic stellate cell senescence, and mitigates liver fibrosis [68]. Oroxylin A further enhances this pathway through multiple mechanisms: it inhibits SIRT7, promotes the succinylation and degradation of PRMT5, and relieves its suppression of cGAS, thereby inducing hepatic stellate cell senescence. Additionally, Oroxylin A facilitates calreticulin externalization, which enhances natural killer (NK) cell recognition and clearance of senescent hepatic stellate cells [69]. Similarly, Oroxylin A potentiates STING pathway activity by inhibiting MAT2A, reducing SAM levels, and alleviating hypermethylation of the cGAS promoter, thereby upregulating cGAS expression [70]. Concurrently, it promotes the accumulation of iron ions and reactive oxygen species via NCOA4-mediated ferritin autophagy, further driving hepatic stellate cell senescence and ultimately attenuating experimental liver fibrosis [71]. Collectively, these studies indicate that TCM-mediated STING activation in fibrotic diseases primarily achieves therapeutic benefit by regulating cell fate, maintaining mitochondrial homeostasis, and facilitating immune clearance.

#### Autoimmune and neuroimmune disorders

In autoimmune and neuroimmune disorders, STING activation exhibits a distinct biological role compared with cancer and infectious diseases, as excessive immune stimulation may aggravate pathological inflammation. Emerging evidence suggests that controlled activation of STING signaling by TCM can restore immune balance through regulation of interferon responses and inflammatory cell polarization.

In an experimental model of autoimmune encephalomyelitis, oxymatrine activates the STING-TBK1-IRF3 signaling axis in microglia, thereby promoting the production of endogenous IFN-β and inhibiting M1-type microglial polarization. This modulation leads to a reduction in the expression of pro-inflammatory cytokines factors such as iNOS and TNF-α, while simultaneously enhancing the levels of anti-inflammatory molecules like IL-10 and IL-27. As a result, oxymatrine alleviates neuroinflammation, demyelination, and neurological dysfunction [72]. Beyond single bioactive compounds, classical TCM prescriptions have also demonstrated immunomodulatory effects through regulation of STING signaling. Shaoyao Gancao Decoction delays the progression of autoimmune hepatitis by restoring splenic immune homeostasis through modulation of the cGAS-STING pathway [73].

These findings suggest that, under specific immune conditions, appropriate activation or fine-tuning of STING signaling does not necessarily exacerbate inflammation but may instead restore immune balance through protective interferon responses and modulation of immune cell polarization.

#### Context-dependent effects and risks of STING modulation

It should be noted that not all STING activation induced by traditional Chinese medicine produces protective effects. In an acute kidney injury model, Esculentoside A triggers mitochondrial dysfunction and the release of mtDNA into the cytoplasm, activating the cGAS-STING pathway and promoting inflammatory responses, epithelial–mesenchymal transition, and renal fibrosis. This suggests that STING activation in this context is closely associated with nephrotoxicity [74]. Similarly, in drug-induced ovarian injury, triptolide induces mitochondrial dysfunction and mtDNA release, activating the cGAS-STING pathway and exacerbating ovarian inflammation, abnormal follicular development, and reduced oocyte quality [75]. Conversely, in spinal cord injury models, dihydromyricetin suppresses rather than activates STING expression, thereby inhibiting the PI3K/AKT signaling pathway and enhancing autophagy, which alleviates oxidative stress, pyroptosis, and secondary neuronal damage in microglia [76].

These findings highlight the context-dependent nature of STING modulation by TCM. The biological consequences of STING activation are determined not only by the intensity and duration of pathway stimulation but also by the specific cellular and pathological environments. Therefore, therapeutic strategies targeting STING should emphasize precise regulation rather than indiscriminate activation or inhibition, particularly when translating TCM-based interventions into clinical applications.

Collectively, TCM-mediated modulation of the STING pathway involves diverse upstream mechanisms, including direct interaction with cGAS/STING, regulation of mitochondrial DNA sensing, and alteration of STING protein stability. Despite these differences, they converge on the transcriptional activation of IFN-Is and chemokines, ultimately driving the recruitment and activation of effector immune cells. This convergence suggests that STING functions as a signaling integration node, allowing structurally diverse natural products to elicit immune responses through a common pathway, regardless of their distinct initial mechanisms. From the perspective of the holistic, multi-component, and multi-target nature of TCM, the STING pathway is not a specific, exclusive target of any single compound but rather a common hub frequently engaged within the broader network of natural product actions. This mechanistic convergence also provides a molecular basis for the observation that different diseases—such as tumors, viral infections, and fibrotic conditions—can share similar therapeutic approaches mediated through STING activation.

### Traditional Chinese Medicine inhibits the STING pathway

In addition to promoting immune activation, sustained and excessive activation of the cGAS-STING pathway is a key driver of various inflammatory diseases and tissue damage. This pathological process usually involves more than STING itself. Persistent cGAS-STING activation often occurs together with NF-κB activation, NLRP3 inflammasome activation, MAPK signaling, oxidative stress, mitochondrial dysfunction, and inflammatory cell death. These pathways can amplify each other and aggravate tissue injury. In TCM theory, this pathological state may be interpreted as excessive pathogenic factors and disruption of yin–yang balance. Therefore, inhibition of aberrant cGAS-STING signaling by TCM-derived compounds or formulas may correspond to clearing excessive pathogenic factors, protecting healthy qi from inflammatory injury, and restoring immune homeostasis. Studies have shown that inhibiting abnormally activated STING signaling can attenuate inflammatory responses and improve disease outcomes [18, 77]. Building on this, certain bioactive components of TCM and natural products can directly inhibit the activation of key molecules within the cGAS-STING pathway, thereby mitigating pathological inflammation [31]. This inhibitory effect is particularly notable in acute and chronic inflammation, organ injury, autoimmune disorders, and metabolism-related diseases. Moreover, in specific tumor microenvironments or in contexts of treatment-related injury, moderate suppression of aberrant STING activation may help reduce chronic inflammation and enhance overall therapeutic outcomes [31].

#### Tumor diseases

In recent years, the role of STING signaling in tumorigenesis and tumor immune regulation has garnered considerable attention. Evidence, particularly in NSCLC (NSCLC), breast cancer, and melanoma, suggests that modulation of STING-related signaling axes may underlie the antitumor effects of natural products.

In NSCLC, berbamine inhibits tumor cell proliferation and migration while alleviating tumor-associated immunosuppression. It achieves this primarily by targeting STING to suppress the expression of p-STING, NF-κB, and CCL2, thereby reducing M-MDSC infiltration and slowing tumor progression [78]. Similarly, ginsenoside Re demonstrates promising therapeutic effects in NSCLC by inhibiting the polarization of tumor-associated macrophages toward the M2 phenotype, suppressing tumor growth and metastasis. Its mechanism is closely linked to the inhibition of the AMPKα1/STING positive feedback loop, thereby blocking both M2 macrophage polarization and epithelial–mesenchymal transition (EMT) [79].

In melanoma, icaritin has been shown to inhibit the formation of an inflammatory pre-metastatic microenvironment and reduce lung metastases [80]. Mechanistically, it suppresses STING activation and downstream signaling pathways—including TBK1, IRF3, and NF-κB—thereby decreasing the expression of inflammatory factors and molecules associated with the pre-metastatic niche [80]. These findings suggest that natural compounds can regulate STING signaling not only to directly influence tumor cell behavior but also to impede distant tumor dissemination by reshaping the host inflammatory response and microenvironment.

In breast cancer, formononetin inhibits tumor cell proliferation and invasion while inducing cell cycle arrest [81]. Its effects are primarily mediated through the inhibition of the STING–NF-κB pathway and the downregulation of PD-L1 expression, which together attenuate the malignant phenotype and reduce immune evasion [81]. This highlights that natural compounds can influence STING signaling to modulate not only inflammatory responses and the tumor microenvironment but also immune checkpoint expression, thereby enhancing antitumor activity.

Overall, natural compounds can modulate STING-related signaling networks through multiple mechanisms, including direct inhibition of STING activation, suppression of downstream inflammatory pathways, reduction of immunosuppressive cell recruitment, regulation of macrophage polarization, and downregulation of PD-L1 expression. Collectively, these actions contribute to anti-proliferative, anti-invasive, and anti-metastatic effects in tumor cells. However, the evidence that these compounds directly inhibit STING signaling is not equally strong. Studies showing direct binding to STING or reversal by pathway agonists provide stronger support. Studies reporting only reduced phosphorylation of STING, TBK1, IRF3, or NF-κB should be interpreted as indirect pathway-associated evidence unless direct target engagement is demonstrated.

#### Other diseases

In addition to its involvement in tumor-related pathological processes, aberrant activation of the STING pathway is also widely observed in a variety of non-neoplastic diseases. Bioactive components and compound formulations derived from traditional Chinese medicine can attenuate inflammatory responses, oxidative stress, and tissue injury by inhibiting cGAS, STING, and their associated signaling pathways. These effects highlight their considerable therapeutic potential across a broad spectrum of systemic disorders.

##### Respiratory diseases

In respiratory diseases, abnormal activation of the STING pathway is closely associated with the amplification of acute inflammation, mitochondrial dysfunction, pulmonary vascular remodeling, and the progression of fibrosis. Current research encompasses multiple conditions, including acute lung injury, sepsis-associated lung injury, viral pneumonia, pulmonary arterial hypertension, and pulmonary fibrosis. Although different compounds act through distinct mechanisms, they generally converge on suppressing the cGAS-STING-mediated inflammatory cascade. Perillaldehyde alleviates lipopolysaccharide (LPS)-induced acute lung injury by inhibiting mitochondrial ROS production and mtDNA release, thereby blocking cGAS-STING-mediated activation of the TBK1, IRF3, and NF-κB signaling pathways [82]. Apigenin reduces pulmonary inflammation and edema primarily by downregulating STING expression and inhibiting IRF3 activation, indicating that its effects are exerted at the midstream and downstream levels of the pathway [83]. In sepsis-induced lung injury, ursodeoxycholic acid not only suppresses STING, TBK1, IRF3, and NF-κB signaling but also inhibits ZBP1-mediated PANoptosis-like cell death [84]. Additionally, Tanreqing Injection mitigates LPS-induced lung injury by reducing mtDNA release and suppressing cGAS-STING-associated inflammatory signaling, and its protective effects can be partially reversed by STING agonists [85].

In virus-associated lung injury, rhamnocitrin binds simultaneously to cGAS and STING, inhibiting the activation of the cGAS, STING, TBK1, IRF3, and NF-κB pathways, and alleviating H3N2 infection-induced oxidative stress and apoptosis [86]. As an inhibitor of HIF-1α, panaxadiol attenuates H9N2-induced pulmonary inflammation by suppressing HIF-1α-dependent activation of cGAS and STING, thereby reducing cytosolic dsDNA accumulation and restoring mitochondrial respiratory homeostasis [87]. The evidence for saikosaponin B1 in influenza viral pneumonia is mainly prediction-based. The study used network pharmacology, transcriptomics, and metabolomics to suggest that cGAS and STING may be potential targets [88]. However, the study did not provide direct target validation, pathway rescue experiments, or genetic evidence. Therefore, this finding should be regarded as hypothesis-generating rather than confirmatory evidence.

In chronic pulmonary vascular and fibrotic diseases, ginsenoside Rg1 alleviates pulmonary vascular cell senescence and improves vascular remodeling associated with pulmonary arterial hypertension by inhibiting the activation of the cGAS-STING-TBK1 signaling pathway [89]. Similarly, 20(S)-protopanaxadiol mitigates lung inflammation, epithelial–mesenchymal transition, and collagen deposition by modulating the AMPK–STING signaling axis, thereby attenuating pulmonary fibrosis [90]. Additional studies indicate that 20(S)-protopanaxadiol may further enhance its antifibrotic effects by regulating the gut microbiota and associated metabolic reprogramming, suggesting a potential link between STING pathway modulation and gut–lung axis remodeling [91].

Overall, TCM-based interventions in respiratory diseases extend beyond simple anti-inflammatory effects to encompass multi-level regulation, including modulation of mtDNA release, cellular senescence, and tissue remodeling. Within this framework, the STING pathway serves as a central signaling hub linking these interconnected pathological processes.

##### Urinary system disorders

Among urinary system diseases, aberrant activation of the STING pathway is closely associated with conditions such as cisplatin-induced acute kidney injury and diabetic nephropathy. A common feature across these studies is that STING activation frequently co-occurs with DNA damage, mitochondrial dysfunction, and immune cell infiltration. Accordingly, traditional Chinese medicine interventions typically combine direct inhibition of the STING pathway with the restoration of upstream cellular damage.

In cisplatin-induced acute kidney injury, 6-shogaol directly targets STING, inhibiting the activation of the cGAS-STING-TBK1 signaling axis and reducing macrophage migration and infiltration, thereby alleviating renal injury [92]. The polydatin-based nanodelivery system exerts protective effects by simultaneously alleviating endoplasmic reticulum stress and DNA damage through dual-targeted delivery, effectively blocking the amplification of cGAS-STING signaling and demonstrating the integration of delivery optimization with mechanism-based intervention [93]. Similarly, naringenin-loaded silk fibroin peptide nanofibers mitigate cisplatin-induced kidney injury by enhancing mitochondrial targeting and promoting PINK1 and Parkin-mediated mitophagy, thereby inhibiting mtDNA release and subsequent activation of the cGAS-STING pathway [94]. In contrast, baicalein-loaded silk fibroin peptide nanofibers primarily suppress cGAS-STING signaling by reducing oxidative stress and DNA damage, leading to improvements in renal function and histopathological outcomes [95].

In diabetic nephropathy, sinomenine alleviates renal inflammatory injury by inhibiting the activation of cGAS, STING, and their downstream effectors, including p-TBK1, p-IRF3, and NF-κB [96]. Germacrone, on the other hand, indirectly suppresses cGAS–STING signaling by restoring PINK1/Parkin-dependent mitophagy, thereby reducing mitochondrial ROS accumulation and mtDNA leakage into the cytoplasm. This, in turn, attenuates ferroptosis and tubular injury [97].

Similarly, Nephropathy II Decoction attenuates renal fibrosis by preserving mitochondrial function and reducing mtDNA-dependent activation of the cGAS–STING pathway [98].

Overall, TCM-mediated regulation of the STING pathway in urinary system disorders reflects a dual strategy that integrates suppression of inflammatory signaling with restoration of organelle homeostasis. Moreover, the incorporation of advanced nanodelivery systems provides promising avenues for improving renal targeting and therapeutic efficacy.

##### Diseases of the digestive system

Diseases of the digestive system represent a primary focus in Traditional Chinese Medicine research on the regulation of the STING pathway. These conditions include intestinal inflammation, drug-induced intestinal injury, liver injury, liver fibrosis, fatty liver disease, and pancreatitis. Research in this area is extensive and provides a comprehensive understanding of the underlying mechanisms. These include not only direct targeting of cGAS and STING but also promoting DNA repair, reducing mtDNA release, improving mitochondrial homeostasis, and protecting barrier function.

In intestinal diseases, naringin alleviates inflammation, apoptosis, and barrier disruption caused by intestinal ischemia–reperfusion by inactivating the cGAS-STING pathway [99]. In models of ulcerative colitis and colitis-associated cancer, curcumol enhances intestinal barrier integrity and reduces colitis by activating PXR, which transcriptionally suppresses STING expression [100]. Toosendanin targets both LYN and STING, inhibiting kinase phosphorylation and blocking STING oligomerization, demonstrating the synergistic advantages of a dual-target approach [101]. Brusatol not only inhibits the cGAS and STING pathways but also ameliorates irinotecan-induced gut microbiota dysbiosis and barrier damage [102]. Andrographolide indirectly suppresses cGAS-STING activation by promoting homologous recombination repair in colonic epithelial cells and reducing dsDNA release [103].

 In liver diseases, naringenin directly targets cGAS, inhibiting activation of the cGAS-STING pathway and reducing hepatic stellate cell activation, thereby alleviating liver fibrosis [104]. Ginsenoside Rd links inhibition of cGAS and STING to the regulation of ferroptosis, suggesting a close association between STING signaling and lipid peroxidation in acute liver injury [105]. Jujuboside B exerts hepatoprotective effects by activating nuclear factor erythroid 2-related factor 2 (Nrf2) and suppressing STING signaling [106]. Similarly, emodin alleviates acetaminophen-induced liver injury by enhancing the Nrf2, HO-1, and NQO1 antioxidant pathways while inhibiting both the NLRP3 inflammasome and the cGAS–STING axis [107]. Magnolol simultaneously inhibits NLRP3 activation, pyroptosis, and the cGAS–STING pathway, thereby mitigating cholesterol-induced liver injury and fibrosis [108]. Puerarin primarily suppresses STING, IRF3, and NF-κB signaling in hepatic macrophages, suggesting that immune cells are key sites of action in metabolic fatty liver disease [109]. Curcumol alleviates iron overload by upregulating ceruloplasmin, reduces mtDNA release, and inhibits the cGAS-STING-NF-κB pathway, improving outcomes in alcohol- and high-fat diet-induced fatty liver [110]. Additionally, curcumol mitigates hepatocyte senescence in alcoholic fatty liver disease by blocking the interaction between LC3B and LaminB1, thereby preventing CCF formation and subsequent cGAS-STING activation [111]. Patchouli alcohol inhibits STING-mediated inflammatory signaling by restoring mitochondrial-associated endoplasmic reticulum membrane integrity and correcting metabolic imbalances [112]. Berberine improves lipid deposition and metabolic dysfunction in non-alcoholic fatty liver disease by activating PI3K/Akt signaling and inhibiting STING, TBK1, and IRF3 pathways [113]. Studies on combined nanomedicine formulations indicate that ivy saponin derivatives can synergistically enhance anti-fibrotic effects by inhibiting STING and NF-κB pathways in macrophages and, when combined with sorafenib, suppress hepatic stellate cell activation [114]. Notably, naringenin exhibits broad hepatoprotective potential that involves not only cGAS and STING but also multiple pathways, including oxidative stress, TGF-β, MAPK, TLR signaling, and hepatic stellate cell activation [115].

In pancreatic diseases, saikosaponin D alleviates oxidative stress and mitochondrial damage, inhibits mtDNA-mediated cGAS-STING activation, and suppresses NLRP3- and caspase-1-dependent pyroptosis [116]. Although studies in this area are limited, current evidence suggests that STING in pancreatitis primarily links mitochondrial damage to inflammatory cell death.

Recent evidence further indicates that classical TCM formulas participate in antifibrotic therapy through modulation of STING signaling. ErTao Decoction alleviates liver fibrosis by suppressing STING-mediated macrophage activation and subsequent NLRP3 inflammasome signaling [117].

In summary, research on digestive system diseases illustrates the multilevel regulatory characteristics of TCM on the STING pathway, encompassing direct targeting, control of danger signals, metabolic remodeling, and microenvironmental improvement.

##### Immune system diseases

In immune system disorders, abnormal activation of the STING pathway is often closely associated with autoinflammation, autoimmune responses, and persistent IFN-I signaling. Compared to other therapeutic areas, this field more clearly demonstrates the advantages of Traditional Chinese Medicine in terms of target specificity, with evidence of direct action on cGAS, STING, or IRF3 being more readily observable.

Salvianolic acid A directly binds to STING, inhibiting the downstream activation of TBK1 and IRF3, and reducing the expression of inflammatory cytokines such as IFN-β, IL-1β, IL-6, and TNF-α. This action alleviates systemic inflammation and multi-organ damage in sepsis [118]. Ginkgetin biloba biflavone binds to the C-terminal domain of STING, blocking its activation, preventing translocation to the Golgi apparatus, and inhibiting its interaction with TBK1 [119]. Similarly, Carnosic acid acts directly on STING’s C-terminal tail, blocking TBK1 recruitment, and is recognized as a typical direct STING antagonist [120]. Licochalcone D inhibits STING oligomerization by covalently modifying the Cys148 site, representing a more direct mechanism of action [121]. Kurarinone, a compound derived from the Sophora genus, also targets STING but more prominently interferes with the interaction between STING and IRF3 [122].

In contrast, dihydrotanshinone I primarily targets IRF3, inhibiting its nuclear translocation and suppressing IFN-I signaling by binding directly to IRF3 [123]. Licochalcone B exerts anti-inflammatory effects by binding to IRF3 and disrupting the STING-TBK1-IRF3 signaling axis [124]. Glabridin reduces abnormal inflammatory responses by inhibiting the interaction between STING and IRF3 [125]. Cordycepin suppresses DNA-stimulated activation of cGAS, STING, and TBK1 signaling by promoting the autophagic degradation of STING [126]. Perillaldehyde directly inhibits cGAS enzymatic activity, reducing cGAMP production and thereby attenuating STING activation at a more upstream level [127].

Thus, research on immune system diseases has delineated a clear spectrum of mechanisms, including cGAS inhibition, direct STING antagonism, promotion of STING degradation, and IRF3 blockade. Compared to other organ systems, this field has moved beyond merely demonstrating the ability to "inhibit the STING pathway" and is progressing toward developing a mechanistic classification with characteristics of modern targeted pharmacology.

##### Cardiovascular and metabolic diseases

In cardiovascular and metabolic diseases, abnormal activation of the STING pathway is closely associated with mtDNA leakage, macrophage inflammation, vascular remodeling, and metabolic imbalance. A key feature of research in this area is that STING functions not only as an amplifier of inflammation but also as a crucial hub linking mitochondrial damage, immune cell activation, and organ remodeling. Consequently, various Traditional Chinese Medicine components exert dual regulatory effects, addressing both inflammation and metabolic improvement.

Chlorogenic acid directly binds to STING, inhibiting its activation as well as the downstream signaling of p-TBK1, IFN-β, and NF-κB p65. This reduces macrophage recruitment and fibrotic remodeling, alleviating stress-induced heart failure and myocardial fibrosis [128]. Curcumol interferes with both acute inflammatory amplification and subsequent remodeling following myocardial infarction. It does so by inhibiting STING dimerization, oligomerization, and its transport to the ERGIC, thereby disrupting the STING–TBK1 interaction [129]. Ginsenoside Rb1 primarily inhibits STING-mediated macrophage activation, suggesting that immune cells are key targets in stress-induced myocardial injury [130]. Mangiferin acts as an upstream intervention, stabilizing Nrf2, reducing reactive oxygen species (ROS), and preventing mtDNA leakage. In doing so, it inhibits cGAS–STING-related signaling [131]. Quercetin promotes mitochondrial autophagy and inhibits STING-related pyroptosis signaling in drug-induced cardiotoxicity, suggesting that STING activation in drug-induced myocardial injury is closely linked to impaired mitochondrial quality control [132]. Beyond hepatic and renal fibrosis, Xin-Ji-Er-Kang improves chronic heart failure by suppressing mtDNA/cGAS-STING signaling through NR3C1-mediated MFN2 upregulation, suggesting that regulation of mitochondrial homeostasis represents an important strategy for controlling pathological STING activation during chronic tissue remodeling [133].

In metabolic and vascular inflammation, EGCG primarily exerts its effects by inhibiting the release of mtDNA and oxidized mtDNA (ox-mtDNA), as well as the activation of TBK1, cGAS, STING, and NLRP3, further emphasizing the triggering role of oxidized mitochondrial DNA in atherosclerosis [134]. Tetrandrine directly suppresses the STING, TBK1, and NF-κB pathways, with a particular focus on blocking inflammatory signaling [135]. Kaempferol acts on the STING–NLRP3 axis in adipose tissue, highlighting the immunometabolic remodeling associated with obesity-related chronic low-grade inflammation [136]. Aurantio-obtusin inhibits cGAS and STING activation in the liver by reducing mtDNA release via extracellular vesicles, suggesting that the STING pathway may also mediate the transmission of injury signals between distant organs [137]. In cardiovascular and metabolic diseases, STING functions not only as an amplifier of inflammation but also as a critical hub linking mitochondrial damage, immune cell activation, and organ remodeling. The therapeutic value of Traditional Chinese Medicine lies in its ability to disrupt this vicious cycle, rather than merely suppressing inflammation. This mechanism corresponds well with the TCM principles of activating blood circulation, resolving stasis, tonifying qi, and unblocking collaterals, which are commonly applied in cardiovascular and metabolic disorders.

##### Neurological disorders

In neurological disorders, abnormal activation of the STING pathway is often associated with excessive microglial activation, neuroinflammation, mitochondrial damage, and cognitive or emotional behavioral abnormalities. Compared to other organ systems, research in this area highlights the role of STING within the neuroimmune microenvironment and emphasizes the regulatory significance of autophagy, lysosomal function, and mitochondrial homeostasis in pathway activation.

Protocatechuic acid inhibits cGAS, STING, and downstream signaling by enhancing microglial autophagy and promoting the clearance of damaged DNA, thereby ameliorating neuroinflammation and motor deficits in Parkinson’s disease models [138]. In brain aging models, punicalin suppresses the cGAS-STING axis while also modulating oxidative stress and glial cell activation [139]. Silybin primarily inhibits STING expression and inflammatory responses in a subarachnoid hemorrhage model, and additionally promotes microglial polarization toward the M2 phenotype [140]. Artesunate inhibits cGAS and STING activation by enhancing lysosomal function, suggesting that impaired intracellular degradation contributes to sustained STING activation in chronic cerebral ischemia [141].

Platycodin D effectively restores mitochondrial function and mitigates mtDNA damage; its inhibition of cGAS and STING is closely linked to reduced Aβ accumulation and improved energy metabolism [142]. Tetrahydroxy stilbene glycoside exerts broad neuroinflammatory inhibition by simultaneously suppressing cGAS, STING, p-TBK1, p-NF-κB, and the NLRP3 inflammasome [143]. Silybin also alleviates hippocampal inflammatory damage in models of depression and anxiety-like behaviors associated with Parkinson’s disease by inhibiting the STING–IRF3–IFN-β signaling pathway [144]. Echinacoside mitigates neuroinflammation and cognitive deficits in sepsis-associated encephalopathy by repairing mitochondrial damage, reducing ROS, and inhibiting cGAS and STING signaling [145].

The distinctive feature of TCM regulation of STING in the nervous system lies in its multi-layered mechanism: interventions not only suppress inflammation but also reshape the neuroimmune microenvironment by maintaining autophagosome function, clearing cytoplasmic DNA, and restoring mitochondrial integrity.

##### Other diseases

Beyond the previously discussed systems, Traditional Chinese Medicine has also been shown to modulate the STING pathway in diseases affecting the musculoskeletal system, skin, reproductive system, and eyes.

Quercetin alleviates pain sensitization and cartilage damage in knee osteoarthritis by inhibiting the cGAS and STING pathways, as well as VEGF-A and VEGF-R1-related signaling [146]. Astilbin mitigates abnormal keratinocyte proliferation and inflammatory damage in psoriasis models by inhibiting ferroptosis and the mtDNA-cGAS-STING pathway [147]. Ginsenoside Rb3 reduces ischemia–reperfusion injury in skin flaps by inhibiting STING and IRF3 signaling, thereby improving microcirculation [148]. Cryptotanshinone binds to specific sites on STING, inhibiting mtDNA, STING, and NF-κB-related signaling while promoting STING degradation via the lysosomal pathway, thereby reducing microglial activation and pathological angiogenesis in ischemic retinopathy [149].

Chlorogenic acid inhibits the activation of oxidized mtDNA (ox-mtDNA), cGAS, and STING, as well as the NLRP3 inflammasome, thereby improving spermatogenic dysfunction associated with varicocele [150]. Astragaloside IV alleviates macrophage senescence and improves age-related osteoporosis by inhibiting the STING and NF-κB pathways [151]. Although these conditions span diverse organ systems, they collectively suggest that the STING pathway plays a pivotal role in chronic inflammation and tissue damage. These findings indicate that TCM interventions across various organs share certain common mechanisms.

Overall, research on the role of TCM in inhibiting the STING pathway spans a broad range of diseases, affecting multiple systems including the respiratory, digestive, urinary, cardiovascular, nervous, immune, and metabolic systems. The mechanisms of action encompass both direct targeting of STING, cGAS, and IRF3, as well as indirect inhibition through modulation of mitochondrial homeostasis, reduction of DNA leakage, enhancement of autophagy and lysosomal function, and suppression of oxidative stress, pyroptosis, and ferroptosis.

In contrast to pathway activation, TCM-mediated inhibition of cGAS–STING signaling also involves multiple molecularly distinct mechanisms. Some compounds directly inhibit cGAS activity, thereby preventing cGAMP synthesis and upstream signal initiation, as exemplified by perillaldehyde. Several compounds function as direct STING antagonists by binding to specific domains of STING. For instance, ginkgetin binds to the C-terminal domain of STING and prevents STING activation and Golgi translocation, whereas carnosic acid targets the STING C-terminal tail and blocks TBK1 recruitment. Licochalcone D represents a site-specific covalent inhibitor that modifies Cys148 of STING and suppresses STING oligomerization. Other compounds interfere with STING trafficking or degradation: curcumol blocks STING dimerization/oligomerization and ERGIC transport, while cordycepin promotes autophagic degradation of STING. In addition, several natural products act downstream by disrupting protein–protein interactions or transcription factor activation. Compound Danshen Dripping Pills block STING–TBK1 complex assembly; glabridin and kurarinone interfere with STING–IRF3 signaling; and dihydrotanshinone I directly target IRF3 to inhibit its nuclear translocation. These mechanisms demonstrate that TCM-derived inhibitors can act at different molecular layers, including ligand sensing, STING structural activation, intracellular trafficking, signalosome assembly, protein degradation, and transcriptional activation (Table 1).

**Table 1:** Traditional Chinese medicine can treat or alleviate diseases by regulating the cGAS-STING signaling pathway

Mechanism	Origins	Molecular formula	Cells/Animals	Mode of administration	Kinds of treatment	Functions	Mechanisms	R	Evidence category
Activate STING	Salvia miltiorrhiza	C₃₆H₃₀O₁₆	cells:4T1,RAW264. 7, A549, Jurkat Animals:BALB/c, C57BL/6J, Kunming mice	Endotracheal infusion	Lung Cancer with Lung Metastases	Inhibits lung metastasis in lung cancer, enhances the efficacy of anti-PD-1 immunotherapy, and alleviates Mn^2^⁺-induced inflammation	SalB acts as a Mn^2^⁺ carrier, promoting its uptake to synergistically activate the STING pathway, thereby recruiting immune cells to eliminate tumors, and alleviating inflammation through its antioxidant effects	[55]	Indirect pathway association
	Stephania tetrandra	C₃₈H₄₂N₂O₆	cells:H358, H2030, LLC1 Animals:C57BL/6J mice	Intraperitoneal injection	Non-small cell lung cancer	Inhibits the growth of non-small cell lung cancer and enhances the efficacy of anti-PD-1 immunotherapy	Inducing DNA damage leads to the accumulation of intracellular double-stranded DNA, which activates the STING pathway to recruit immune cells and enhance antitumor immunity	[47]	Indirect pathway association
	Nelumbinis Plumula	C₃₇H₄₂N₂O₆	Cells:Jurkat, Molt4, Hut102, EL4, NK-92MI Animals:BALB/c Naked mice, NOD/SCID mice, C57BL/6 mice	Gastric lavage	T-cell malignancies	Enhance the ability of NK cells to eliminate T-cell malignancies, inhibit tumor growth, and prolong survival	By targeting PRDX1, ROS-dependent mitochondrial damage is induced, inhibiting PINK1/Parkin-mediated mitochondrial autophagy, leading to the accumulation of mitochondrial DNA in the cytoplasm, thereby activating the cGAS-STING pathway	[50]	Indirect pathway association
	Panax ginseng	C₃₆H₆₂O₈	Cells:HT29, LoVo, T84､RKO, HCT116 etc Animals:BALB/c nude mice	Intratumoral injection	Colorectal cancer	Inhibits the proliferation of colorectal cancer cells and tumor growth, and enhances the anticancer effects of IFN-γ	It synergistically activates the STING pathway with IFN-γ, promoting the transcription of type I interferons and the expression of downstream genes, and induces tumor cell apoptosis via the JAK1/2 pathway	[57]	Indirect pathway association
	Astragalus membranaceus	C₄₁H₆₈O₁₄	Cells:4T1, MDA-MB-231､MCF-7 Animals:BALB/c mice	Oral administration and gastric lavage	Triple-negative breast cancer	To enhance the efficacy of cisplatin in the treatment of triple-negative breast cancer and to mitigate cisplatin-induced weight loss, nephrotoxicity, and immunosuppression	It induces mitochondrial damage, promoting the release of mtDNA, which activates the cGAS-STING pathway to upregulate IFN-β and chemokines, leading to cell cycle arrest and apoptosis in tumor cells	[46]	Indirect pathway association
	Cucurbitaceae	C₃₂H₄₆O₈	Cells:BT-549, HCC1954, MDA-MB-231, 4T1 etc Animals:M-NSG､BALB/c､MMTV-PyMT mice	Intraperitoneal injection	Malignant breast cancer	Inhibits the growth of breast cancer and its metastasis to the lungs, and enhances the efficacy of anti-PD-1 immunotherapy	Covalent targeting of MTCH2 induces mitochondrial damage, promoting the release of mtDNA, which activates the STING pathway to induce neutrophil differentiation and thereby activate T cells	[51]	Indirect pathway association
	Artemisia annua	C₁₅H₂₄O₅	Cells:Lewis, A549, RAW264. 7, HUVEC Animals:C57BL/6 mice	Intraperitoneal injection	Lung cancer	Inhibits the proliferation of lung cancer cells and lung metastasis, induces pyroptosis, and enhances tumor immunogenicity	Targeting TOM70 induces mitochondrial damage, promoting the release of mtDNA, which triggers pyroptosis and immunogenic cell death via the cGAS/STING/NLRP3 pathway	[52]	Indirect pathway association
	Paris formosana	C₅₁H₈₂O₂₀	Cells:H1299, A549, LLC Animals:C57BL/6 mice	Intraperitoneal injection	Non-small cell lung cancer	Inhibits the proliferation, migration, and lung metastasis of non-small cell lung cancer cells; reduces the infiltration of MDSCs and Tregs within tumors; and increases the infiltration of CD8⁺ T cells	Activation of the STING pathway downregulates CXCL1 to inhibit MDSC recruitment, thereby reshaping the immune microenvironment and enhancing antitumor immunity	[196]	Indirect pathway association
	Magnolia species	C₁₈H₁₈O₂	Cells:THP-, RAW264. 7, 293T, B16F10 etc Animals:STING WT/KO, humanized mice	Oral	Melanoma, colon cancer, breast cancer	Inhibit tumor growth, prolong survival, and enhance the efficacy of anti-PD-(L)1 immunotherapy	It directly binds to STING, activates the STING/TBK1/IRF3 pathway, induces the expression of type I interferons and chemokines, and promotes the tumor infiltration and activation of CD8⁺ T cells	[44]	Direct target verification
	Haematoxylum braziletto	C₁₆H₁₄O₅	Cells:A549, H358	–	Non-small cell lung cancer	Inhibits the proliferation, colony formation, and invasion of non-small cell lung cancer cells, and induces G2/M phase arrest and apoptosis	It induces mitochondrial dysfunction and ROS production, activates the STING/TBK1/IRF3 pathway, and upregulates the expression of the chemokine CXCL10,this activation contributes to its antiproliferative effects	[53]	Indirect pathway association
	Amaryllidaceae	C₁₆H₁₇NO₄	Cells:H358, A549, BEAS-2B, LLC1 Animals:BALB/c nude mice, C57BL/6 mice	Intraperitoneal injection	Non-small cell lung cancer	Inhibits the proliferation, invasion, and tumor growth of non-small cell lung cancer cells; induces apoptosis; and increases tumor infiltration by CD8⁺ T cells	Induces mitochondrial dysfunction, leading to STING activation, while also inhibiting p70S6K signaling, thereby promoting CD8^+^T-cell-mediated antitumor immunity	[197]	Indirect pathway association
	Cucurbitaceae	C₃₂H₄₆O₈	Cells:MG63, K7M2 Animals:C57BL/6 mice	Intraperitoneal injection	Osteosarcoma	Inhibits osteosarcoma cell proliferation and tumor growth, induces G2/M phase arrest and apoptosis, and enhances the efficacy of anti-PD-L1 immunotherapy	Induced DNA damage activates the STING pathway, upregulating IFN-γ/CCL5 to reshape the immune microenvironment (increasing effector T cells and reducing suppressor T cells)	[49]	Indirect pathway association
	Plants	C₈H₈O₄	Cells:THP-1/PBMC macrophage, 293T, SKBR3 etc Animals:BALB/c mice	Intraperitoneal injection	Breast cancer, lung cancer	Activates the STING pathway in macrophages, induces M1 macrophage polarization, enhances phagocytosis and induces tumor cell apoptosis, promotes CD8⁺ T cell infiltration, and inhibits tumor growth	Direct activation of STING induces IFN-β/CXCL10 expression and promotes M1 polarization, synergistically enhancing immune responses and inducing apoptosis	[45]	Indirect pathway association
	Aglaia species	C₂₉H₃₁NO₇	Cells:A549, H1299, H1975, LLC Animals:C57BL/6 mice	Intraperitoneal injection	Non-small cell lung cancer	Promotes NK cell infiltration, enhances antitumor immunity, and inhibits tumor growth	Inducing mtDNA damage promotes the release and activation of STING, which upregulates CCL5/CXCL10 to recruit NK cells	[198]	Indirect pathway association
	Stephania tetrandra	C₃₈H₄₂N₂O₆	Cells: HepG2, A549, L929, THP-1 Animals: C57BL/6J mice	Intraperitoneal injection	Viral infection	Inhibits the replication of various viruses (VSV, H1N1, EMCV, HSV-1) and alleviates the inflammatory response caused by viral infections	By targeting NPC1, it blocks viral entry and inhibits STING degradation, thereby enhancing antiviral immunity	[60]	Indirect pathway association
	Plant-based	C₁₅H₁₀O₆	Cells: HaCaT, Vero, BV2, RAW264. 7, SH-SY5Y Animals: BALB/c mice	Gastric lavage	Herpes Simplex Virus Type 1 Encephalitis	Inhibits HSV-1 infection and replication, and alleviates symptoms of HSV-1-induced encephalitis, neuroinflammation, and neuronal damage	Directly activates cGAS to promote its oligomerization, triggering the STING pathway to induce type I interferon and enhance antiviral immunity	[58]	Direct target verification
	Glycyrrhiza uralensis	C₃₀H₄₆O₄	Cells: Calu-3, Vero E6, MEF Animals: K18-hACE2 Transgenic mice, Balb/c mice	Oral administration via gastric tube	COVID-19	Inhibits the replication of the SARS-CoV-2 Omicron variant and alleviates virus-induced pulmonary inflammation and pathological damage	Targets specific serine/threonine sites on STING to activate the signaling pathway, inducing IFN-β and ISGs to enhance antiviral immunity	[59]	Direct target verification
	Schisandra chinensis	C₂₂H₂₄O₆	Cells: THP-1, L929, HEK-293T, AML12, Primary PBMCs Animals: C57BL/6 mice	Intraperitoneal injection	Hepatitis B	Inhibits hepatitis B virus replication and reduces serum levels of HBsAg, HBeAg, and HBV DNA	By promoting the interaction between STING and TBK1, it enhances the activation of the STING/TBK1/IRF3 pathway, upregulates the expression of type I interferon (IFN-β) and ISGs, and strengthens the antiviral immune response	[61]	Direct target verification
	Phyllanthus	C₂₇H₂₂O₁₈	Cells: HFF, MRC5, NIH3T3, THP-1; Animals: BALB/c mice	Gastric lavage	Cytomegalovirus infection	Inhibits the replication of human cytomegalovirus and mouse cytomegalovirus, and alleviates virus-induced pathological damage to liver and lung tissue	Upregulates STING expression and activates the STING/TBK1/IRF3/NF-κB pathway, thereby promoting type I interferon production and antiviral innate immunity	[62]	Indirect pathway association
	Stephania	C₃₇H₃₈N₂O₆	Cells: Vero, HeLa	–	Herpes simplex virus type 1 infection	Inhibits HSV-1 replication and reduces the expression of viral proteins and genes	By activating the STING/TBK1/P62 signaling pathway, it induces interferon-independent autophagy, promoting the autolytic degradation of viral particles and viral proteins, thereby inhibiting HSV-1 infection	[63]	Indirect pathway association
	Euphorbia fischeriana	–	Cells: Bone marrow-derived macrophages,iBMDM, HEK293T, HeLa, Vero Animals: C57BL/6 mice	intravenous injection	Viral infection	Inhibits VSV and HSV-1 replication, enhances antiviral immunity, and protects mice from lethal HSV-1 infection	It activates innate immunity via STING-dependent mechanisms, upregulating the expression of interferon-stimulated genes (ISGs) through the IRF3/IRF7/ELF4 transcription factor complex, thereby enhancing the antiviral immune response	[64]	Indirect pathway association
	Phytolacca americana	C₄₂H₆₆O₁₆	Cells: Human iPSC-derived kidney organoids Animals: C57BL/6J mice	Intraperitoneal injection	Acute Kidney Injury	Induces damage, inflammation, and fibrosis in renal tubular epithelial cells, leading to reduced kidney function	It induces mitochondrial dysfunction and the release of mtDNA into the cytoplasm, activates the cGAS-STING pathway, leading to the expression of downstream inflammatory factors, and promotes epithelial-mesenchymal transition (EMT) and renal fibrosis	[74]	Indirect pathway association
	Sophora flavescens	C₁₅H₂₄N₂O₂	Cells: BV2 Animals: C57BL/6 mice	Intraperitoneal injection	Experimental autoimmune encephalomyelitis (a model of multiple sclerosis)	Alleviates neurological dysfunction, demyelination, and inflammation in EAE mice; inhibits M1-type polarization and the expression of inflammatory factors in microglia; and promotes M2-type polarization and the expression of anti-inflammatory factors	By activating the STING/TBK1/IRF3 signaling pathway in microglia, it promotes the production of IFN-β, thereby suppressing neuroinflammation and autoimmune responses	[72]	Indirect pathway association
	Tripterygium wilfordii	C₂₀H₂₄O₆	Cells: Mouse oocytes Animals: mice	Intraperitoneal injection	Ovarian toxicity	It reduces ovarian function and fertility, impairs follicle development, induces ovarian inflammation and mitochondrial dysfunction, and compromises oocyte quality	This induces mitochondrial dysfunction and increased mitochondrial membrane permeability, leading to the release of mtDNA into the cytoplasm, which activates the cGAS-STING pathway, resulting in ovarian inflammation and impaired oocyte meiosis and maturation	[75]	Indirect pathway association
	Ampelopsis grossedentata	C₁₅H₁₂O₈	Cells: BV2 Animals: C57BL/6 mice	Gastric lavage	Spinal cord injury	Improves motor dysfunction in mice with spinal cord injury, reduces neuronal damage, and inhibits pyroptosis and oxidative stress	By inhibiting STING expression, blocking the PI3K/AKT signaling pathway, and promoting autophagy, this approach suppresses pyroptosis and oxidative stress in microglia, thereby alleviating secondary damage	[76]	Indirect pathway association
	Ligusticum chuanxiong/Angelica sinensis	C₁₂H₁₄O₂	Cells: LX-2, HSC-T6, Primary HSC Animals: C57BL/6J mice	Intraperitoneal injection/Gastric lavage	Liver fibrosis	Inhibits the activation of hepatic stellate cells, alleviates experimental liver fibrosis, and induces senescence in activated HSCs	Disruption of G-actin leads to nuclear membrane damage and the nuclear export of YAP, promoting the release of dsDNA, which activates STING and induces HSC senescence	[68]	Indirect pathway association
	Scutellaria baicalensis	C₁₆H₁₂O₅	Cells: LX-2, HSC-T6Animals: ICR mice	Gastric lavage	Liver fibrosis	Induce senescence in activated hepatic stellate cells to alleviate CCL₄-induced liver fibrosis	Inhibition of SIRT7 promotes PRMT5 degradation, activates the cGAS-STING pathway to induce HSC senescence, and promotes CRT eversion to enhance NK cell clearance	[69]	Indirect pathway association
	Scutellaria baicalensis	C₁₆H₁₂O₅	Cells: LX-2, HSC-T6, LO2Animals: ICR mice	Gastric lavage	Liver fibrosis	Induce senescence in activated hepatic stellate cells to alleviate CCL₄-induced liver fibrosis	Inhibition of MAT2A reduces SAM, which promotes cGAS demethylation, upregulates its expression, activates the STING pathway, and induces HSC senescence	[70]	Indirect pathway association
	Scutellaria baicalensis	C₁₆H₁₂O₅	Cells: LX-2, Primary HSC Animals: ICR mice	Gastric lavage	Liver fibrosis	Induce senescence in activated hepatic stellate cells to alleviate CCL₄-induced liver fibrosis	By activating the cGAS-STING pathway, it promotes the secretion of downstream cytokines, upregulates NCOA4—a key molecule in ferritin phagocytosis—induces ferritin phagocytosis, leading to the accumulation of iron ions and ROS, and ultimately induces HSC senescence	[71]	Indirect pathway association
Inhibition of STING in tumors	Berberis amurensis	–	Cells: H460, H2122, A549, H358, H23, H1975, PC9, BEAS-2B, CCD19-Lu, 293T; Animals: C57BL/6J mice, LLC model; Human: lung cancer samples	In vitro treatment, intraperitoneal injection, intratumoral injection of 2′,3′-cGAMP	Non-small cell lung cancer	Inhibits tumor cell proliferation and migration and alleviates the tumor-induced immunosuppressive microenvironment	By targeting STING, this approach inhibits the expression of p-STING/NF-κB and CCL2 and reduces M-MDSC infiltration, thereby suppressing tumor progression	[78]	Direct target verification
	Epimedium brevicornu Maxim	–	Cells: B16BL6, MLE-12, MH-S; Animals: C57BL/6 mice	In vitro treatment, intraperitoneal injection, MP tail vein injection, tumor cell tail vein injection	Melanoma	Inhibits the formation of the pre-metastatic inflammatory microenvironment and reduces lung metastasis	By inhibiting STING activation and its downstream TBK1/IRF3/NF-κB signaling pathway, it reduces the expression of inflammatory factors and PMN-associated molecules	[80]	Indirect pathway association
	Red Clover and Astragalus	–	Cells: MCF-7, MDA-MB-468	In vitro processing	Breast cancer	Inhibits the proliferation and invasion of breast cancer cells and induces cell cycle arrest	Inhibits the malignant phenotype of tumors by suppressing the activation of the STING-NF-κB pathway and downregulating PD-L1 expression	[81]	Indirect pathway association
	Panax spp.	–	Cells: THP1, A549, NCI-H1703, NCI-H460; Animals: C57 mice, LLC models	In vitro processing, intraperitoneal injection	Non-small cell lung cancer	Inhibits M2 polarization of tumor-associated macrophages and suppresses tumor growth and metastasis	Inhibiting tumor progression by blocking the polarization of M2 macrophages and the epithelial-mesenchymal transition (EMT) process through suppression of the AMPKα1/STING positive feedback loop	[79]	Indirect pathway association
STING Inhibition—Other Diseases	Salvia miltiorrhiza Bunge	C₂₆H₂₂O₁₀	Cells: RAW264. 7, THP-1; Animals: mice	Tail vein injection, in vitro pretreatment	Sepsis	Improves survival rates in mice with sepsis, alleviates lung, liver, and kidney damage, and reduces the release of inflammatory cytokines	SAA can directly bind to STING and inhibit the activation of its downstream TBK1/IRF3 signaling pathway, thereby reducing the release of inflammatory cytokines and alleviating sepsis-associated inflammatory responses, pulmonary neutrophil infiltration, and multi-organ damage	[118]	Direct target verification
	Cistanche deserticola Y. C. Ma	C₃₅H₄₆O₂₀	Cells: BV2; Animals: mice	Gastric lavage, Intranasal administration, subcutaneous microinjection	Sepsis-associated encephalopathy	Improve cognitive impairment, reduce neuroinflammation, and restore mitochondrial function	ECH alleviates neuroinflammation and cognitive dysfunction in sepsis-associated encephalopathy by improving mitochondrial damage, reducing ROS accumulation, and inhibiting the activation of the cGAS-STING signaling pathway, thereby reducing the expression of pro-inflammatory factors	[145]	Indirect pathway association
	Nervilia fordii and other medicinal plants	C₁₆H₁₂O₆	Cells: MDCK, A549	In vitro administration	Infection with the H3N2 influenza A virus	Inhibits viral replication, reduces cell apoptosis, enhances cell viability, and lowers the expression of inflammatory factors	RH inhibits the activation of the cGAS/STING pathway while alleviating mitochondrial damage, thereby suppressing apoptosis and inflammation	[86]	Indirect pathway association
	Panax ginseng	–	Cells: MH-S; Animals: mice	Intraperitoneal injection	H9N2 avian influenza virus infection	Reduce lung inflammation and damage, improve survival rates, and promote lung repair	Inhibition of HIF-1α blocks the cGAS-STING pathway and repairs mitochondria, thereby alleviating pulmonary inflammatory damage	[87]	Indirect pathway association
	Panax ginseng	C₃₀H₅₂O₃	Animals: mice	Gastric lavage	Pulmonary fibrosis	Alleviates bleomycin-induced pulmonary fibrosis, reduces collagen deposition and inflammation, and improves quality of life	PPD regulates AMPK/STING and metabolic reprogramming, and mediates gut microbiota-targeted gut-lung axis signaling to synergistically alleviate pulmonary fibrosis	[91]	Indirect pathway association
	Panax ginseng	C₄₂H₇₂O₁₄	Animals: rats	Gastric lavage	Pulmonary hypertension	Reduce right ventricular systolic pressure, improve pulmonary hemodynamics and right ventricular hypertrophy, and alleviate phenotypes associated with pulmonary vascular remodeling and cellular senescence	Inhibiting the cGAS/STING/TBK1 pathway downregulates p16/p21 and SASP, thereby alleviating pulmonary vascular senescence-induced remodeling	[89]	Indirect pathway association
	Perilla frutescens	C₁₀H₁₄O	Cells: RAW264. 7; Animals: mice	Intraperitoneal injection, in vitro pretreatment	Acute Lung Injury	Alleviates LPS-induced pulmonary histopathological damage, pulmonary edema, inflammatory cell infiltration, oxidative stress, and cytokine release	PAH inhibits mROS and mtDNA release, thereby blocking the cGAS-STING signaling pathway, reducing inflammatory factors, and alleviating acute lung injury	[82]	Indirect pathway association
	Flavonoid	C₁₅H₁₀O₅	Cells: THP-1, HEK293T; Animals: mice	Intraperitoneal injection, in vitro pretreatment	Acute Lung Injury	Inhibits STING agonist-induced expression of type I interferon and inflammatory genes, and alleviates LPS-induced lung tissue inflammation and edema	Apigenin downregulates STING and blocks its interaction with and activation of IRF3, thereby suppressing innate immune inflammation and alleviating acute lung injury	[83]	Indirect pathway association
	Natural bile acids	C₂₄H₄₀O₄	Animals: mice	Gastric lavage pretreatment	Lung Injury	Alleviates pulmonary edema, inflammatory cell infiltration, cytokine release, oxidative stress, and damage to the pulmonary barrier, and improves alveolar fluid clearance	UDCA alleviates sepsis-induced inflammation, oxidative stress, impaired lung barrier function, and lung injury by inhibiting the activation of the STING/TBK1/IRF3/NF-κB signaling pathway and blocking ZBP1-mediated PANoptosis-like cell death	[84]	Indirect pathway association
	Bupleurum spp.	C₄₂H₆₈O₁₃	Animals: mice	Gastric lavage	Influenza-associated pneumonia, acute lung injury	Improves lung tissue damage caused by influenza virus infection, reduces the inflammatory response, lowers levels of inflammatory cytokines in the lungs, and provides protection against infection-related lung injury	SSB1 exerts its anti-influenza viral pneumonia effects by regulating metabolic inflammation (involving cGAS-STING and other pathways), with the cGAS-STING pathway emerging as a potential mechanism based on multi-omics analysis	[88]	Network pharmacology prediction
	Compound preparations of traditional Chinese medicine	–	Cells: RAW264. 7, mouse bone marrow neutrophils; Animals: mice	Intraperitoneal injection, in vitro processing	Acute Lung Injury	Alleviates pathological damage to lung tissue, pulmonary edema, inflammatory cell infiltration, oxidative stress, and the release of inflammatory cytokines, and improves ALI-related lung injury	TRQ inhibits the release of mtDNA and the activation of the cGAS-STING pathway, downregulates the expression of key molecules, reduces inflammatory factors and ROS, and alleviates acute lung injury; this effect can be partially reversed by STING agonists	[85]	Indirect pathway association
	Panax ginseng Saponin metabolites	C₃₀H₅₂O₃	Cells: MLE-12; Animals: mice	Gastric lavage	Pulmonary fibrosis	Improve survival rates, reduce the lung index, alleviate pulmonary inflammation and collagen deposition, and suppress fibrosis-related phenotypes	PPD activates AMPK, which inhibits STING, thereby attenuating EMT and pulmonary fibrosis; this effect can be attenuated by AMPK inhibitors	[90]	Indirect pathway association
	Polygonum cuspidatum	C₂₀H₂₂O₈	Cells: HK-2, HUVEC; Animals: mice, rats	Gastric lavage, In vitro processing	Acute Kidney Injury	Increase cell survival, reduce ROS accumulation, mitochondrial membrane potential decline, Ca^2^⁺ release, apoptosis, and renal dysfunction; improve renal histopathological changes; and enhance the bioavailability of polydatin	Fu-4-PBA/Po dual-targeted inhibition of endoplasmic reticulum stress and the cGAS-STING pathway alleviates cisplatin-induced acute kidney injury	[93]	Indirect pathway association
	Zingiber officinale	C₁₇H₂₄O₃	Cells: HK-2, THP-1, RAW264. 7; Animals: mice	Intraperitoneal injection / In vivo administration, in vitro processing	Kidney Injury	Reduces serum creatinine and blood urea nitrogen levels, improves renal tissue damage, reduces macrophage infiltration in the kidneys, and decreases the expression of CCL2, CCL5, and inflammatory factors	6-Shogaol alleviates cisplatin-induced kidney injury by targeting STING and inhibiting the activation of the STING-TBK1 signaling pathway, thereby downregulating the expression of CCL2, CCL5, IL-1β, IL-6, and TNF-α and suppressing macrophage migration and infiltration; furthermore, its protective effect is significantly reduced following STING knockout	[92]	Direct target verification
	Sinomenium acutum	C₁₉H₂₃NO₄	Animals: db/db mice	Gastric lavage	Diabetic Nephropathy	Improve overall health, lower blood glucose and HbA1c levels, improve renal function markers and reduce renal pathological damage, and alleviate inflammation-related damage associated with diabetic nephropathy	SIN inhibits the cGAS-STING pathway and downstream inflammatory signaling, thereby reducing renal inflammatory damage; its efficacy is comparable to that of the STING inhibitor C-176	[96]	Indirect pathway association
	Curcuma spp	C₁₅H₂₂O	Cells: HK-2; Animals: db/db mice, C57BL/6J mice	In vitro processing, intraperitoneal injection	Diabetic Nephropathy	Reduces ferroptosis, ROS accumulation, and mitochondrial damage in renal tubular cells, and improves renal dysfunction, glycogen deposition, and fibrosis associated with diabetic nephropathy	6-Shogaol targets and inhibits the activation of the STING pathway, downregulates chemotactic and inflammatory cytokines to reduce macrophage recruitment, and alleviates kidney injury; this effect is STING-dependent	[97]	Indirect pathway association
	Flavanone	Naringenin: C₁₅H₁₂O₅	Cells: HK-2; Animals: mice	Gastric lavage, In vitro processing	Acute Kidney Injury	Enhances cellular vitality, reduces the accumulation of reactive oxygen species (ROS), mitigates damage to mitochondrial membrane potential and renal tissue pathology, lowers serum creatinine (SCr) and blood urea nitrogen (BUN) levels, and boosts antioxidant capacity	SFP-NGN nanofibers deliver naringenin to target specific sites, thereby enhancing mitochondrial autophagy, inhibiting mtDNA release and cGAS-STING activation, and alleviating kidney injury	[94]	Indirect pathway association
	Flavonoid	Baicalein: C₁₅H₁₀O₅	Cells: HK-2; Animals: mice	Gastric lavage, in vitro processing	Acute Kidney Injury	Enhance the water solubility, stability, and cellular uptake of BA; reduce damage to HK2 cells, ROS accumulation, and disruption of mitochondrial membrane potential; and improve renal function and pathological damage in mouse kidney tissue	SFP-BA nanofibers deliver baicalein to enhance mitochondrial targeting, inhibit oxidative stress and DNA damage, and improve antioxidant levels, thereby blocking the DNA damage–cGAS–STING pathway and alleviating kidney injury	[95]	Indirect pathway association
	Ginkgo biloba	C₃₂H₂₂O₁₀	Cells: MEFs, THP-1-derived macrophages, RAW264. 7, HEK293T, HeLa, BMDMs; Animals: mice, Trex1⁻/⁻ mice	Intraperitoneal injection, in vitro processing	Autoimmune diseases	Alleviates Dox- or radiation-induced cellular senescence, reduces the proportion of β-galactosidase-positive cells and the expression of p16, p21, IL-6, and IL-1β in senescent cells, improves multi-organ inflammation and physical decline in aged mice, and alleviates systemic inflammation associated with Trex1 deficiency	By binding to the carboxy-terminal end of STING, it inhibits its activation and translocation, thereby blocking the STING-TBK1 interaction and downstream signaling, and suppressing inflammatory aging	[119]	Direct target verification
	Rosmarinus officinalis	C₂₀H₂₈O₄	Cells: BMDMs, THP-1, hPBMCs, HEK293, HEK293T, L929, 3T3; Animals: mice, Trex1⁻/⁻ mice	Intraperitoneal injection, in vitro processing	Autoimmune diseases	Inhibits the expression of inflammatory cytokines and type I interferons induced by DNA stimulation or STING agonists, alleviates DMXAA-induced systemic inflammation, and improves Trex1-deficient-associated systemic inflammation	Carnosic acid binds to the carboxy-terminal end of STING, thereby blocking TBK1 recruitment and inhibiting downstream signaling phosphorylation and the expression of inflammatory cytokines	[120]	Direct target verification
	Chalcone natural products	C₂₁H₂₂O₅	Cells: THP-1-derived macrophages, RAW264. 7, BMDMs, THP1-Blue ISG, RAW-Lucia ISG, HeLa, 293 T; Animals: C57BL/6 J mice, Trex1⁻/⁻ mice	Intraperitoneal injection, in vitro processing	Inflammatory Bowel Disease	Inhibits cGAS- and STING-mediated expression of inflammatory cytokines and type I interferons, alleviates systemic inflammation associated with Trex1 deficiency, and reduces the risk of colon cancer associated with DSS-induced colitis and AOM/DSS-induced colitis	Licochalcone D inhibits STING oligomerization by covalently modifying the Cys148 site of STING, thereby blocking TBK1 recruitment and the nuclear translocation of IRF3 and NF-κB. This action suppresses downstream inflammatory signaling and alleviates STING-driven inflammatory diseases	[121]	Direct target verification
	Salvia miltiorrhiza	C₁₈H₁₂O₃	Cells: BMDMs, THP-1, hPBMCs, HEK-293, HEK-293T; Animals: C57BL/6 mice, Trex1⁻/⁻ mice	Intraperitoneal injection, in vitro processing	Autoimmune diseases	Inhibits the expression of IFN-β, ISGs, and inflammatory factors; alleviates systemic inflammation associated with Trex1 deficiency; mitigates obesity-related impaired glucose tolerance and insulin resistance; and improves high-fat diet-induced NASH and liver fibrosis	DHT binds to IRF3, interfering with its interaction with STING, thereby blocking the output of the cGAS-STING and RIG-I-MAVS pathways, reducing inflammation, and improving IRF3-driven diseases	[123]	Direct target verification
	Cordyceps spp.	C₁₀H₁₃N₅O₃	Cells: Hela, L929, BMDMs, PBMCs, HEK293; Animals: mice, Trex1 KO mice	Intraperitoneal injection, in vitro processing	Autoimmune diseases	Inhibits DNA-stimulation-induced expression of type I interferons and ISGs, alleviates systemic autoinflammation in Trex1 knockout mice, and reduces IFNB and ISG expression in PBMCs from SLE patients	Promotes STING autophagy-mediated degradation, thereby inhibiting the cGAS-STING signaling pathway and inflammation, and improving autoimmune/inflammation-related pathologies	[126]	Indirect pathway association
	Glycyrrhiza spp	C₂₀H₂₀O₄	Cells: BMDMs, human PBMCs, HEK-293, HEK-293 T; Animals: mice, Trex1⁻/⁻ mice	Intraperitoneal injection, in vitro processing	Autoimmune diseases	Inhibits the expression of inflammatory factors such as IFN-β, IL-6, and TNF-α induced by cGAS-mediated STING activation, alleviates DMXAA-induced inflammatory responses, and reduces multi-organ inflammatory damage in Trex1-deficient mice	Glabridin inhibits the interaction between STING and IRF3, thereby blocking cGAS-STING signaling and reducing the resolution of autoinflammatory responses mediated by interferons and inflammatory factors	[125]	Indirect pathway association
	Sophora tonkinensis Radix et Rhizoma	C₂₆H₃₀O₆	Cells: BMDMs, THP-1; Animals: C57BL/6J mice, BALB/c mice	In vitro treatment, gavage	Autoimmune hepatitis	Reduces the expression of IFN-β and inflammatory factors; alleviates weight loss, colonic shortening, splenomegaly, intestinal barrier damage, and inflammatory responses in DSS-induced colitis; and improves ConA-induced liver damage and inflammation	By targeting STING to interfere with its interaction with IRF3, this approach inhibits pathway activation and the release of inflammatory cytokines, thereby improving colitis and autoimmune hepatitis	[122]	Direct target verification
	Saxifraga and related plants in the Liliaceae family	C₂₁H₂₂O₁₁	HaCaT cells	In vitro treatment	Psoriatic inflammation	Alleviates IL-17-induced hyperproliferation and inflammation in keratinocytes, reduces oxidative stress and ferroptosis, and restores mitochondrial function and cytokine homeostasis	Inhibits ferroptosis and the mtDNA-cGAS-STING signaling pathway, reduces oxidative stress and iron accumulation, and regulates key molecules such as GPX4	[147]	Indirect pathway association
	Licorice	C₁₆H₁₄O₄	BMDMs, THP-1 cells, HEK-293 T cells, WT mice, Trex1⁻/⁻ mice	Intraperitoneal injection, in vitro processing	Autoimmune inflammation	Inhibits the cGAS-STING signaling pathway, reduces the production of type I interferons and inflammatory factors, and alleviates systemic inflammation in Trex1⁻/⁻ mice	By binding to IRF3 and disrupting the STING-TBK1-IRF3 signaling axis, it inhibits IRF3 phosphorylation and nuclear translocation, thereby reducing the expression of Ifnb, IFIT1, ISG15, CXCL10, IL-6, and TNF-α	[124]	Direct target verification
	Shiso	C₁₀H₁₄O	L929 cells, MEF cells, HFF cells, BMDMs, WT mice, Trex1⁻/⁻ mice	Gastric lavage、in vitro processing	Autoimmune inflammation	Inhibits the cytoplasmic DNA-induced interferon response, increases susceptibility to HSV-1, and alleviates autoinflammation triggered by endogenous DNA in Trex1⁻/⁻ mice	Directly inhibits cGAS enzymatic activity, reduces cGAMP production, and suppresses TBK1 and IRF3 phosphorylation, IRF3 dimerization, and nuclear translocation, thereby decreasing the expression of related interferon and inflammatory genes	[127]	Direct target verification
	Salvia miltiorrhiza	C₁₉H₂₀O₃	OIR mice, BV2 cells, HRECs, Sting⁻/⁻ mice	Gastric lavage, intraperitoneal injection, in vitro processing	Ischemic retinopathy	Reduces microglial activation and pathological retinal angiogenesis, alleviates vascular leakage, improves retinal structure and function, and acts synergistically with anti-VEGF agents	By binding to the Ser162 site of STING, it inhibits the STING-mediated NF-κB signaling pathway, reduces mtDNA release and mitochondrial damage, blocks STING translocation, and promotes lysosomal degradation	[149]	Direct target verification
	Four Wonders Eternal Peace Decoction	C₁₆H₁₈O₉	TAC mice, H9C2 cells	Gastric lavage, subcutaneous injection, in vitro processing	Myocardial fibrosis	Improves cardiac function, reduces myocardial fibrosis, decreases inflammatory cell infiltration, and reduces the recruitment of CCR2⁺ macrophages	Directly binds to STING, inhibits STING/pTBK1/IFN-β and NF-κB p65 signaling, suppresses pyroptosis, and reduces macrophage recruitment and fibrotic remodeling	[128]	Direct target verification
	Rhizoma Curcumae / Curcumae Rhizoma	–	Cells: RAW264. 7, MPMs, iBMDMs, BMDMs, HEK293T, H9C2, HL-60; Animals: WT mice, Sting − / − mice	Gastric lavage, intraperitoneal injection, in vitro processing	Myocardial infarction (MI)	Increases survival rates in MI mice, improves cardiac function, reduces infarct size, myocardial cell apoptosis, fibrosis, and adverse ventricular remodeling, promotes resolution of inflammation, and inhibits macrophage-mediated inflammation	By directly binding to STING, it inhibits its activation and transport, disrupts the STING-TBK1 interaction, and downregulates inflammatory factors, this protective effect is STING-dependent	[129]	Direct target verification
	Panax ginseng C. A. Mey	–	Cells: RAW264. 7 macrophages; Animals: OVX mice	Intraperitoneal injection, in vitro processing	Acute catecholamine surge-induced stress-induced cardiomyopathy (SCM)/myocardial injury	Alleviates ISO-induced myocardial injury, myocardial cell necrosis, and myocardial inflammation, and reduces macrophage infiltration	Inhibits cytoplasmic DNA recognition and STING-mediated macrophage activation, thereby reducing the production of inflammatory cytokines and interferons induced by ctDNA/DMXAA	[130]	Indirect pathway association
	Anemarrhena asphodeloides Bunge; It is also widely found in plants such as mangoes	C₁₉H₁₈O₁₁	Cells: H9c2 cardiomyocytes; Animals: mice	Gastric intubation, intraperitoneal injection, in vitro processing, AAV9 cardiac-specific knockdown	Sepsis-associated cardiomyopathy / LPS-induced cardiac injury	Increases survival rates in mice exposed to endotoxin, improves cardiac function, and reduces myocardial structural damage, oxidative stress, macrophage recruitment, and inflammatory responses	Promoting Keap1 degradation activates Nrf2, alleviates mitochondrial damage, and inhibits mtDNA leakage, thereby blocking the cGAS-STING and TLR9-NF-κB pathways	[131]	Indirect pathway association
	Astragalus membranaceus/A. mongholicus	C₄₁H₆₈O₁₄	Cells: RAW264. 7, BMSCs, BMDMs; Animals: mice	Gastric lavage, in vitro processing	Age-related osteoporosis	Inhibits macrophage senescence and M1 polarization, promotes M2 polarization, enhances osteogenic differentiation of BMSCs, and reduces bone loss	Inhibits H₂O₂-induced activation of the STING/NF-κB signaling pathway, reduces levels of STING, p65/p65, and pIκBα/IκBα, alleviates ROS accumulation and mitochondrial membrane potential decline, and downregulates aging and pro-inflammatory factors	[151]	Indirect pathway association
	Honeysuckle	C₁₆H₁₈O₉	Animals: rats	Gastric lavage	Male infertility model	Repair pathological damage to the seminiferous tubules, increase sperm count, restore the integrity of the blood-testicular barrier, and reduce oxidative stress and testicular inflammation	Reduces mitochondrial structural damage and excessive ROS levels, inhibits the OXMTDNA/cGAS-mediated STING pathway and NLRP3 inflammasome activation, downregulates inflammatory factors; and upregulates tight junction proteins to protect the blood-testis barrier	[150]	Indirect pathway association
	Tea polyphenols	C₂₂H₁₈O₁₁	Cells: HAECs, BMDMs; Animals: mice	Injection into the caudal vein, in vitro processing	Atherosclerosis (AS)	Improves cardiac hemodynamics, reduces atherosclerotic plaques and myocardial fibrosis, and maintains endothelial mitochondrial homeostasis	Inhibits mitochondrial oxidative stress and the release of mtDNA/oxmtDNA; inhibits the activation of TBK1/cGAS/STING and NLRP3; reduces ASC-spot formation and IL-1β release	[134]	Indirect pathway association
	Flavonoid	C₁₅H₁₀O₇	Cells: Primary DRG neurons, PC-12 cells; Animals: C57BL/6J mice	Gastric lavage, intraperitoneal injection, in vitro processing	Knee osteoarthritis	Alleviates KOA pain sensitization, reduces the expression of inflammatory factors and pain-related molecules, and improves cartilage pathology	Inhibits the cGAS/STING signaling pathway, reduces the expression of VEGF-A, VEGFR1, and pVEGFR1, and downregulates pain-related molecules, STING agonists can reverse these effects	[146]	Indirect pathway association
	Flavonoid	C₁₅H₁₀O₇	Cells: AC16 cardiomyocytes; Animals: nude mice	Intraperitoneal injection, in vitro treatment, shRNA transfection	Model of cardiomyocyte pyroptosis	Improves cardiac function, reduces ventricular fibrosis, inhibits pyroptosis in cardiomyocytes, and protects mitochondrial function	By regulating the SHP2/ROS/AMPK/XBP-1/DJ-1 axis, this approach promotes mitochondrial autophagy, reduces ROS, and inhibits p-STING/p-IRF3/NLRP3/GSDMD/IL-1β signaling	[132]	Indirect pathway association
	Flavonoid	C₁₅H₁₀O₆	Cells: RAW264. 7 macrophages; Animals: db/db mice	Gastric lavage, in vitro processing	Insulin resistance	Reduce body weight and body fat, improve glucose tolerance and insulin resistance, and reduce inflammation in adipose tissue	Inhibits the STING/NLRP3/caspase-1/IL-1β signaling pathway in adipose tissue, reduces M1 macrophage infiltration, and increases M2 markers	[136]	Indirect pathway association
	Panax spp.	C₅₃H₉₀O₂₂	Cells: HDMECs, THP-1 cells; Animals: rats	Abdominal cavity / Preoperative and postoperative administration, in vitro processing	Microcirculatory disorders in flap transplantation	Improve flap survival rates, enhance microcirculatory perfusion, and reduce inflammatory infiltration and microthrombosis	Inhibits the activation of the STING-IRF3 signaling pathway, reduces the expression of STING, pIRF3, and P-selectin, and suppresses IRF3 nuclear translocation, STING agonists can reverse these effects	[148]	Indirect pathway association
	Stephania tetrandra	–	Cells: mouse primary peritoneal macrophages (MPMs); Animals: ApoE⁻/⁻ mice	Gastric lavage, in vitro processing	Atherosclerosis (AS)	Reduces the area of aortic plaques, decreases macrophage/monocyte/neutrophil infiltration, alleviates inflammation and fibrosis, inhibits foam cell formation, and slows the progression of atherosclerosis	Inhibits the activation of the STING/TBK1/NF-κB pathway, downregulates key molecules and downstream inflammatory factors, and counteracts the effects of various inducers	[135]	Indirect pathway association
	Protocatechuic acid	C₇H₆O₄	Cells: primary microglia; Animals: MPTP-induced PD mice, A30P transgenic mice, Atg5 microglia conditional knockout mice	Intraperitoneal injection, in vitro processing	Parkinson's disease (PD)	Improves motor dysfunction, reduces the loss of dopaminergic neurons, suppresses neuroinflammation, reduces α-syn aggregation, and improves the disease course in A30P mice	Enhances autophagy to promote the clearance of damaged DNA, inhibits the cGAS-STING pathway and downstream inflammation, and exerts a neuroprotective effect that is Atg5-dependent	[138]	Indirect pathway association
	Punica granatum	C₃₄H₂₂O₂₂	Cells: BV2 microglia, N2a cells; Animals: D-gal-induced aging mice, naturally aged mice	Gastric lavage; intraperitoneal injection of D-galactose; in vitro treatment	Cognitive impairment	Improves learning, memory, and anxiety-like/exploratory behavioral abnormalities; reduces oxidative stress, neuroinflammation, neuronal damage, and synaptic impairment; and decreases glial cell activation	Inhibition of the cGAS-STING axis downregulates inflammatory factors and alleviates neuroinflammation and oxidative stress; this effect can be reversed by STING agonists	[139]	Indirect pathway association
	Flavonol	C₂₅H₂₂O₁₀	Cells: primary microglia, BV2 cells; Animals: experimental SAH mice; Human: SAH patients CSF	Gastric lavage; in vitro processing	Subarachnoid hemorrhage (SAH)	Improves neurological function and motor performance; reduces cerebral edema, neuronal damage, and apoptosis; inhibits inflammatory damage; and promotes the polarization of microglia toward the M2 phenotype	Inhibition of STING expression reduces inflammation and promotes M2 polarization; this effect can be reversed by STING agonists	[140]	Indirect pathway association
	Artemisia annua	C₁₉H₂₈O₈	Cells: HT22 cells, primary rat neurons; Animals: BCCAO rats	Gastric lavage; in vitro processing	Chronic cerebral ischemia	Improves learning, memory, and exploratory behavior; reduces brain tissue damage, cerebral infarction, and neuronal apoptosis; and protects neurons from OGD-induced damage	It activates lysosomal function, inhibits the cGAS-STING pathway, downregulates inflammatory factors, and upregulates IL-10; this effect can be reversed by lysosomal inhibitors	[141]	Indirect pathway association
	Triterpenoid Saponins	C₅₇H₉₂O₂₈	Cells: APPswe cells (SH-SY5Y stably transfected with APPswe gene); Animals: APP/PS1 mice	Intracerebral injection; in vitro processing	Alzheimer's disease (AD)	Improves spatial learning and memory deficits as well as anxiety/depression-like behaviors; reduces Aβ plaque and soluble Aβ levels; and alleviates mitochondrial structural and functional damage	By inhibiting the activation of the cGAS-STING pathway, reducing inflammation and mtDNA damage, and improving mitochondrial function, cGAMP can be reversed	[142]	Indirect pathway association
	Polygonum multiflorum	C₂₀H₂₂O₉	Cells: BV2 microglia; Animals: APP/PS1 mice, C57BL/6J mice	Gastric lavage; in vitro processing	Alzheimer's Disease (AD) / Neuroinflammation Models	Improves learning and memory, suppresses central and peripheral inflammation, reduces microglial activation, and improves cognitive impairment	Inhibits the cGAS-STING and downstream NLRP3 pathways, reduces pro-inflammatory factors, and promotes the transition of M1 microglia to a resting state	[143]	Indirect pathway association
	Flavonol	C₂₅H₂₂O₁₀	Animals: MPTP-induced PD mice	Gastric lavage; intraperitoneal injection	Parkinson's disease (PD)	Improves depression- and anxiety-like behaviors, reduces damage to hippocampal neurons, restores 5-HT and NA levels, and reduces neuroinflammation	Inhibits the activation of the STING-IRF3-IFN-β pathway, downregulates the expression of inflammatory factors and caspase-1, and alleviates inflammatory damage in the hippocampus	[144]	Indirect pathway association
	Magnolia officinalis	C₁₈H₁₈O₂	Cells: HepG2 cells, BMDMs; Animals: C57BL/6 mice	Gastric lavage; in vitro processing; high-cholesterol diet intervention	Liver fibrosis model	Improves liver dysfunction, inflammation, and fibrosis caused by high cholesterol; reverses lipid metabolism disorders; and reduces hepatocyte death	Inhibiting the NLRP3/pyroptosis/cGAS-STING pathways reduces damage and restores cholesterol metabolism	[108]	Indirect pathway association
	Brucea javanica	C₂₆H₃₂O₁₁	Animals: BALB/c mice	Gastric lavage; intraperitoneal injection	Delayed-onset diarrhea	Alleviates weight loss, reduces DAI scores, improves colonic shortening, alleviates increased intestinal permeability and colonic pathological damage, suppresses inflammation and repairs the intestinal barrier, and regulates dysbiosis	By inhibiting the abnormal activation of cGAS-STING, it downregulates inflammatory factors and upregulates IL-10 and tight junctions; this effect can be reversed by STING agonists	[102]	Indirect pathway association
	Hederagenin	–	Cells: JS-1, RAW264. 7, HepG2, AML12; Animals: CCl4-induced liver fibrosis mice	Injection into the tail vein; in vitro processing	Liver fibrosis	Improves liver function markers, reduces inflammatory cell infiltration, inhibits collagen deposition and α-SMA/COL1 expression, enhances liver-targeted delivery efficiency, and strengthens the anti-fibrotic effect	Sorafenib targets HSCs, while Hed inhibits STING in macrophages, synergistically blocking the fibrosis-inflammation feedback loop	[114]	Indirect pathway association
	Naringin	C27H32O14	Cells: IEC-6; Animals: intestinal I/R injury rats	Gastric lavage; in vitro pretreatment	Intestinal ischemia–reperfusion injury	Alleviates damage to the intestinal mucosa and damage to distant organs, reduces inflammation and oxidative stress, decreases cell apoptosis, and improves cell viability	Inactivation of the cGAS-STING pathway suppresses inflammation and apoptosis; multiple studies have demonstrated that it directly targets cGAS to mediate protective effects	[99]	Direct target verification
	Curcuma longa L	–	Cells: HCoEpiC, LS174T, BMDMs; Animals: DSS-induced colitis mice, AOM/DSS CAC mice, PXR−/−mic, STING−/−mice	Gastric lavage; intraperitoneal injection of partial agonists/antagonists; in vitro treatment	Ulcerative colitis (UC)	Reduces colonic inflammation and tissue damage, improves body weight, DAI, and histopathological scores, restores barrier integrity, and inhibits inflammation-related tumor progression	It activates PXR to suppress STING, thereby blocking the NF-κB inflammatory pathway; multiple studies have demonstrated that this mechanism mediates its anti-inflammatory effects	[100]	Indirect pathway association
	Cassia obtusifolia/Senna obtusifolia	–	Cells: primary brown adipocytes, primary white adipocytes, AML12; Animals: HFHS-induced obese mice, BAT/WAT transplantation mice, Sting−/−mice	Gastric lavage; tail vein injection of EVs; transplantation model; in vitro treatment	Obesity	Improves systemic lipid deposition, enhances thermogenesis in brown adipose tissue (BAT), reduces liver inflammation and metabolic abnormalities, and blocks the transmission of damage signals from BAT to the liver	Restores BAT mitochondrial function and enhances autophagy, thereby preventing the release of mtDNA and inhibiting the cGAS/STING inflammatory pathway in the liver	[137]	Indirect pathway association
	Melia toosendan	C30H38O11	Cells: RAW264. 7, THP-1-derived macrophages, BMDMs; Animals: DSS-induced acute colitis mice	Intraperitoneal injection, in vitro processing	Ulcerative colitis (UC)	Alleviates DSS-induced colitis, improves weight loss, DAI, colonic shortening, pathological damage, and goblet cell loss, restores the intestinal barrier, and inhibits macrophage inflammatory infiltration	Dual-target inhibition: It directly binds to LYN to block its phosphorylation, while simultaneously binding to STING to inhibit its oligomerization, thereby downregulating signaling pathways and inflammatory factors	[101]	Direct target verification
	Pueraria montana var. lobata (Willd.)	–	Cells:BMDMs, AML12, HepG2 Animals:C57BL/6J	Gastric lavage, intraperitoneal injection, in vitro processing	Non-alcoholic fatty liver disease	Reduces high-fat diet-induced hepatic steatosis, inflammation, and metabolic disorders, and improves liver function and glucose and lipid metabolism	Inhibits the activation of the STING-IRF3/NF-κB pathway in hepatic macrophages, reduces the release of TNF-α and IFN-β, and improves lipid metabolism abnormalities in hepatocytes	[109]	Indirect pathway association
	Curcuma longa	–	Cells:BNL CL. 2, Primary HSCs Animals:C57BL/6J mice	Gastric lavage, in vitro treatment, plasmid transfection	Fatty liver	Improves liver damage, lipid accumulation, inflammation, and HSC activation	Increasing ceruloplasmin alleviates iron overload, reduces mtDNA release, and inhibits the cGAS-STING and NF-κB pathways as well as hepatic stellate cell activation	[110]	Indirect pathway association
	Rhizoma Curcumae	—	Cells:LO2 Animals:C57BL/6J mice	Gastric lavage, in vitro treatment, plasmid transfection, lentiviral tail vein injection	Alcoholic fatty liver disease	Alleviates ethanol-induced hepatic lipid accumulation, liver damage, and hepatocyte senescence, and reduces SASP inflammatory factors	Blocking the LC3B–Lamin B1 interaction inhibits CCF formation and cGAS-mediated STING activation, thereby alleviating hepatocyte senescence	[111]	Indirect pathway association
	Pogostemon cablin/Pogostemonis Herba		Animals:SD rats	Gastric lavage	Steatohepatitis	Reduce body weight and liver fat accumulation, improve fatty liver, inflammation, and liver damage	Upregulation of Mfn2 suppresses MAM dysregulation, alleviates endoplasmic reticulum stress and mitochondrial dysfunction, inhibits STING-mediated inflammatory signaling, and improves SREBP1c/PPARα-mediated lipid metabolism disorders	[112]	Indirect pathway association
	Rhizoma Coptidis	–	Cells:HepG2 Animals:C57BL/6J mice	Gastric lavage, in vitro processing	Fatty liver disease	Alleviates fatty liver, inflammation, and impaired glucose tolerance; improves disorders of glucose and lipid metabolism	Activates the PI3K/Akt pathway, inhibits the STING-TBK1-IRF3 pathway, and reduces lipid accumulation; AKT inhibitors can reverse this effect	[113]	Indirect pathway association
	Bupleurum falcatum	–	Cells:AR42J	In vitro processing	Acute pancreatitis	Alleviates cerulein-induced damage to pancreatic acinar cells, cytokine release, and pyroptosis	By reducing oxidative stress and mitochondrial damage, it inhibits cGAS-STING activation mediated by mtDNA release, thereby suppressing NLRP3/caspase-1-dependent pyroptosis	[116]	Indirect pathway association
	Andrographis paniculata	–	Cells:HCT116､HT29､CT26;Animals:BALB/cmice､C57BL/6mice	Intraperitoneal injection, gastric lavage, in vitro processing	Intestinal mucositis	Alleviates CPT-11-induced intestinal mucosal damage, weight loss, increased intestinal permeability, and inflammatory response, without affecting antitumor activity	By promoting homologous recombination-mediated DNA repair in colonic epithelial cells, it reduces the release of double-stranded DNA, thereby inhibiting the activation of the cGAS-STING pathway	[103]	Indirect pathway association
	Panax ginseng	–	Animals:C57BL/6mice	Intraperitoneal injection	Acute Liver Injury	Alleviates CCl₄-induced acute liver injury, ferroptosis, oxidative damage, and abnormal liver function	By inhibiting ferroptosis and downregulating the activation of the cGAS-STING pathway, it reduces oxidative DNA damage and inflammatory responses	[105]	Indirect pathway association
	Ziziphi Spinosae Semen	–	Animals:C57BL/6Jmice	Intraperitoneal injection, gastric lavage	Liver Injury	Alleviates liver damage, oxidative stress, inflammatory responses, and hepatocyte apoptosis caused by acetaminophen overdose	By activating Nrf2 and promoting its nuclear translocation, it upregulates HO-1 and NQO-1 while simultaneously inhibiting the STING signaling pathway to exert a protective effect	[106]	Indirect pathway association
	Rheum palmatum	–	Animals:C57BL/6mice	Gastric lavage, intraperitoneal injection	Induced liver damage	Alleviates APAP-induced liver tissue damage, oxidative stress, inflammation, and hepatocyte apoptosis	By upregulating the Nrf2/HO-1/NQO1 antioxidant pathway while simultaneously inhibiting the NLRP3 inflammasome and cGAS-STING signaling pathways	[107]	Indirect pathway association
	Citrus plant components	C₁₅H₁₂O₅	Cells:LX2､L02;Animals:C57BL/6Jmice;Human Tissue: Liver Cancer and Peritumoral Tissue	Gastric lavage, intraperitoneal injection, in vitro processing	Liver fibrosis	Alleviated CCl₄-induced liver damage, collagen deposition, inflammatory response, and the degree of liver fibrosis	By directly targeting cGAS, inhibiting the activation of the cGAS-STING pathway, and reducing the secretion and activation of inflammatory factors by hepatic stellate cells (HSCs)	[104]	Direct target verification

**Table 2:** Traditional Chinese medicine can treat or alleviate diseases by regulating the STING signaling pathway as a complex structure

Mechanism	Compounds	Origins	Cells/ Animals	Mode of administration	Kinds of treatment	Functions	Mechanisms	R	Evidence category
Tumor	Ba Zi Bu Shen Capsules (BZBS)	Traditional Chinese herbal formula	Cells: Hepa1-6, RAW264. 7, THP-1; Animals: C57BL/6mice, STING knockout mice	Infected Hepa1-6 cells	Liver cancer	Enhancing the immune clearance of liver cancer cells that have aged due to chemotherapy or oncogene induction	By inducing mitochondrial damage and dysfunction in macrophages, this process leads to the leakage of mitochondrial DNA into the cytoplasm, thereby activating the cGAS-STING signaling pathway, recruiting CD8⁺ T cells, promoting antitumor immunity, and eliminating senescent tumor cells	[153]	Indirect pathway association
	Qianlie Xiaozheng formula(QLXZF)	Traditional Chinese herbal formula	Cells: RM-1, Primary CD8+ T cells from mice; Animals: BALB/cmice	Gastric lavage	Prostate cancer	Inhibits prostate cancer growth, induces DNA damage and apoptosis in tumor cells, and increases the infiltration of CD4+, CD8+ T cells, and NK cells	By suppressing CHK1 expression, DNA damage accumulates, thereby activating the cGAS-STING-TBK1-IRF3 signaling pathway, increasing the expression of IFN-β and its downstream chemokines CCL5 and CXCL10, and promoting an antitumor immune response. Overexpression of CHK1 reverses this antitumor effect	[154]	Indirect pathway association
	Feiwei Mixture(FWHJ)	Traditional Chinese herbal formula	Cells: LLC, Primary CD8+ T cells from mice; Animals: BALB/cmice	subcutaneous injection,Gastric lavage	Non-small cell lung cancer	Inhibits tumor growth in non-small cell lung cancer (NSCLC), promotes tumor cell apoptosis, and enhances CD4+ /CD8+ T-cell infiltration	By upregulating the expression of the nuclear receptor NR1D1, the cGAS-STING pathway is activated (increasing p-STING, p-TBK1, and p-IRF3), and the SOCS3-JAK-STAT3 signaling axis is inhibited, ultimately leading to increased release of immune-related factors such as CCL5, CXCL10, and IFN-α	[155]	Indirect pathway association
	Schisandrin C (SC) & Luteolin (Lut) Combination therapy	Liuwei Wuling Tablets (LWWL)	HepG2. 2. 15 cells, THP-1 cells, C57BL/6J mice	intravenously injection	Hepatitis B-related liver cancer	Inhibit HBV replication	Lut: Downregulates hepatic nuclear factor 4α (HNF4α) via the MAPK/ERK pathway, thereby inhibiting HBV replication; SC: Promotes activation of the cGAS-STING pathway in macrophages, increasing IFN-β production, which indirectly inhibits HBV replication in hepatocytes	[156]	Indirect pathway association
	Epimedium brevicornu Maxim. extract (EPE)	Epimedium brevicornu Maxim	Cells: NK-92, Human primary NK cells, K562, Hepa1-6; Animals: C57BL/6Jmice	Gastric lavage	Liver cancer	Inhibiting the growth of hepatocellular carcinoma (HCC) by activating NK cells	It directly activates the cGAS-STING signaling pathway in NK cells, promotes the release of IFN-β, and upregulates the phosphorylation levels of STING and IRF3, thereby enhancing NK cell activity and their ability to kill tumor cells	[183]	Indirect pathway association
	Sophora tonkinensis extract (STE)	Sophora tonkinensis Gagnep	Cells: BMDMs, THP-1, HepG2. 2. 15; Animals: C57BL/6Jmice	Gastric lavage	Hepatitis B-related liver cancer	Inhibits HBV replication and improves liver pathology	It promotes the synthesis of 2′3'-cGAMP by cGAS, thereby enhancing the activation of the cGAS-STING pathway; it stimulates the production of type I interferons and inflammatory cytokines in macrophages; it inhibits HBV replication in hepatocytes via paracrine mechanisms; and it enhances the T-cell immune response in vivo	[66]	Indirect pathway association
	ADNVs (Artemisia-Derived Nanovesicles)	Artemisia annua	Cells:LLC, RAW264. 7, BMDMs; Animals:C57BL/6mice	Intraperitoneal injection	Lung cancer	Inhibiting lung cancer growth and enhancing the efficacy of immune checkpoint inhibitor therapy by reprogramming tumor-associated macrophages (TAMs)	ADNVs are preferentially taken up by TAMs; the plant-derived mtDNA they contain acts as an effector molecule, activating the cGAS-STING signaling pathway within TAMs, driving the transformation of TAMs from a pro-tumor M2 phenotype to an anti-tumor M1 phenotype, thereby enhancing CTL responses and suppressing tumor growth	[184]	Indirect pathway association
	Quercetin-Mn2⁺ nanoparticles (AHA@MnP/QCT NPs)	It can be derived from various traditional Chinese medicines	Cells: Lewis, A549; Animals: C57BL/6mice	intravenous injection	Non-Small Cell Lung Cancer	Implementing MRI Imaging and Collaborative Treatment for Non-Small Cell Lung Cancer	QCT induces apoptosis; Mn^2^⁺ depletes GSH and generates ROS via a Fenton-like reaction, thereby inducing ferroptosis; Mn^2^⁺ also activates the cGAS-STING pathway, inducing immunogenic cell death (ICD), and promotes DC maturation and T cell activation	[186]	Indirect pathway association
	Dendrobium polysaccharide hydrogel (MnP@DOP-Gel)	Dendrobium	Cells:B16-F10, BMDMs, BMDCs; Animals:C57BL/6mice	Postoperative tumor cavity implantation	Melanoma	As a postoperative implant, it responds to the ROS environment at the surgical site by continuously releasing Mn^2^⁺, thereby inhibiting tumor recurrence and metastasis	Through the sustained release of MnP microspheres from ROS-responsive hydrogels, Mn^2^⁺ exerts a dual effect: 1) inducing immunogenic cell death (ICD) in residual tumor cells; and 2) activating the cGAS-STING pathway in dendritic cells (DCs) and macrophages, promoting M1 polarization and DC maturation, thereby initiating a cascade of antitumor immune responses and enhancing the efficacy of aPD1	[187]	Indirect pathway association
	DNAzyme-Glycyrrhizic Acid Nanosystem	Licorice	Cells:B16F10, 4T1, RAW264. 7; Animals:C57BL/6Jmice	intravenous injection	Cancer Immunotherapy	Inhibits the growth of primary tumors, prevents distant metastasis, and activates multiple anti-tumor immune responses	Mn^2^⁺ activates the cGAS-STING pathway, promoting type I interferon production and dendritic cell maturation. The DNAzyme cleaves PD-L1 mRNA, inducing gene silencing and reversing immune evasion. Photodynamic therapy induces immunogenic cell death (ICD)	[188]	Indirect pathway association
	Pian Zai Huang Nanoparticle Formulation (PZH/Zn@CaNA)	Traditional Chinese herbal formula	Cells: Hepa1-6; Animals: C57BL/6J mice	intravenous injection	Liver cancer	Restoring the immunosuppressive microenvironment following incomplete microwave ablation to inhibit the rapid growth of residual tumors	PZH inhibits the JAK2-STAT3 signaling pathway, directly suppressing the proliferation of residual tumors. In an acidic TME, it releases Ca^2^⁺/Zn^2^⁺, leading to mitochondrial calcium overload and oxidative stress, which generates large amounts of ROS, induces pyroptosis, and releases DAMPs. Pyroptosis and ROS jointly activate the cGAS-STING pathway, thereby reshaping the immunosuppressive microenvironment	[189]	Indirect pathway association
	Glycyrrhizic acid-copper nanoparticles (cLipG/CuET)	Licorice	Cells:HuCCT1, THP-1; Animals:C57BL/6J mice	intravenous injection	Bile duct cancer	Targets mitochondria, induces copper-mediated cell death and mitochondrial damage, activates the cGAS-STING pathway, and enhances the efficacy of immunotherapy for cholangiocarcinoma	Through mitochondrial-targeted delivery of GA and CuET, GA activates MPTP to facilitate the entry of Cu^2^⁺ into mitochondria, inducing copper-mediated cell death and mitochondrial dysfunction, which leads to the release of mtDNA into the cytoplasm. This, in turn, activates the cGAS-STING pathway in macrophages, promotes M1 polarization, transforms “cold” tumors into “hot” tumors, and exerts a synergistic effect with αCTLA-4	[190]	Indirect pathway association
	Paridis Saponin II Nanoplatform (PZ@M-T)	Paridis	Cells:HepG2, Hepa1-6; Animals: C57BL/6J mice	intravenous injection	Liver cancer	Specifically activates the cGAS-STING pathway, induces ferroptosis in hepatocellular carcinoma cells, reshapes the immune microenvironment, and inhibits the progression of hepatocellular carcinoma	Mitochondria-targeted delivery of PPII induces intense ferrocytosis, leading to the release of mitochondrial DNA (mtDNA). The released mtDNA acts in concert with Zn^2^⁺ generated from vector degradation to specifically activate the cGAS-STING pathway, thereby driving DC maturation, M1 polarization of TAMs, enhanced CTL infiltration, and suppression of Tregs	[191]	Indirect pathway association
Infectious diseases	Nano-adjuvant CFCP (containing curcumin)	Turmeric	DC2. 4, neutrophils; Animals:ICR mice	Local injection	Implant-related infections	Eliminate biofilm infections on implants and prevent recurrence and reinfection	Under ultrasound, CFCP enhances the SDT effect, disrupts the bacterial biofilm, and exposes bacterial dsDNA. Iron ions interfere with bacterial energy metabolism, accelerating bacterial death. The dsDNA released upon bacterial death activates the cGAS-STING pathway in DCs, promoting DC maturation and IFN-β secretion. IFN-β activates neutrophils, enhancing their NET formation, degranulation, and phagocytic functions. Activated DCs promote antibody production by B cells and the formation of memory B cells, providing long-term protection	[192]	Indirect pathway association
Respiratory system	Shuangdan Jiedu Decoction (SJD)	Traditional Chinese herbal formula	Cells:BMDMs,THP-1; Animals:C57BL/6mice	Gastric lavage	Acute Lung Injury	Alleviates LPS-induced pulmonary pathological damage and reduces levels of neutrophils and inflammatory cytokines	Through the synergistic action of multiple Chinese herbal medicines, it simultaneously inhibits the abnormal activation of the cGAS-STING pathway and various inflammasomes (NLRP3, AIM2, NLRC4)	[157]	Indirect pathway association
	Yinqiao San (YQP)	Traditional Chinese herbal formula	Cells:BMDMs, THP-1, Human PBMCs; Animals:C57BL/6J mice	Gastric lavage	Acute Lung Injury	Alleviates LPS-induced pulmonary pathological damage, pulmonary edema, and inflammatory cytokine levels	It specifically inhibits the cGAS-STING signaling pathway. Mechanistically, it does not affect STING oligomerization or the STING-TBK1 interaction, but rather inhibits the phosphorylation and nuclear translocation of IRF3 by blocking the interaction between TBK1 and IRF3, thereby reducing the production of type I interferons and inflammatory cytokines	[158]	Indirect pathway association
	Tanreqing injection (TRQ)	Traditional Chinese herbal formula	C57BL/6 mice	Intraperitoneal injection	Pulmonary fibrosis	Alleviates BLM-induced pulmonary edema, inflammatory cell infiltration, and collagen deposition, and improves lung function	By inhibiting the expression of the STING protein and the endoplasmic reticulum stress (ERS) signaling pathway it mediates, while simultaneously inhibiting the phosphorylation of NF-κB p65, this approach alleviates inflammatory responses and the fibrotic process	[159]	Indirect pathway association
	Qingfei Xieding Formula (QF)	Traditional Chinese herbal formula	Cells:MLE-12; Animals:C57BL/6 mice	Gastric lavage	Pulmonary fibrosis	Alleviates bleomycin-induced lung tissue damage, collagen deposition, and inflammatory cell infiltration, and reduces mortality	By activating autophagy, it reduces mitochondrial damage and mtDNA leakage, thereby inhibiting the mtDNA-cGAS-STING pathway and the subsequent nuclear translocation of NF-κB p65 and the expression of inflammatory cytokines (IL-6, TNF-α)	[160]	Indirect pathway association
	BuFeiDecoction(BFD)	Traditional Chinese herbal formula	Cells: Jurkat T cells, human primary T cells; Animals:C57BL/6J mice	Gastric lavage	Age-related COPD	Improves lung function in aged mice with COPD, reduces lung inflammation, restores the number of naive T cells, and delays T-cell senescence	Increases C1qbp expression in CD4⁺ T cells, inhibits the abnormal activation of the cGAS-STING signaling pathway, and reduces the expression of downstream type I interferons, inflammatory factors, and aging-related factors	[161]	Indirect pathway association
Urinary System Disorders	Yi-Shen-Xie-Zhuo Formula (YSXZF)	Traditional Chinese herbal formula	HKC-8 human renal tubular epithelial cells C57BL/6 mice	Gastric lavage	Acute Kidney Injury (AKI)	Improves renal function and reduces tubular damage; inhibits apoptosis in renal tubular epithelial cells; reduces renal inflammatory responses; improves mitochondrial function	Downregulates the expression of cGAS and STING proteins and their mRNAs, thereby inhibiting the phosphorylation of downstream TBK1 and IRF3	[162]	Indirect pathway association
	Shenqi Fuzheng Injection (SQFZ)	Traditional Chinese herbal formula	Cells:HK-2, 4T1; Animals:BALB/c mice	Intraperitoneal injection	Adjuvant chemotherapy	To reduce cisplatin-induced kidney damage, oxidative stress, and apoptosis without compromising the antitumor efficacy of cisplatin	By alleviating mitochondrial dysfunction and reducing mtDNA leakage, it inhibits the activation of the cGAS-STING signaling pathway, thereby reducing the production of downstream inflammatory cytokines (such as IL-6, IL-8, and CCL5) and cell apoptosis	[163]	Indirect pathway association
	Fu-4-PBA/Po NPs (Fucoidan-based Nanoparticles)	Fucoidan is derived from brown algae; polydatin is derived from Japanese knotweed	Cells:HK-2, HUVEC; Animals:Kunming mice, SD rats	Oral、Gastric lavage	Acute Kidney Injury (AKI)	Alleviates cisplatin-induced kidney damage, oxidative stress, and apoptosis, and improves kidney function	Dual-targeted delivery: 1. Fucoidan targets P-selectin, which is highly expressed at sites of inflammation; 2. 4-PBA targets the endoplasmic reticulum and, upon release, inhibits endoplasmic reticulum stress. By alleviating endoplasmic reticulum stress, it reduces mitochondrial dysfunction and DNA damage, thereby inhibiting the activation of the cGAS-STING pathway and alleviating inflammation	[93]	Indirect pathway association
	Yi-Qi-Jian-Pi-Xiao-Yu formula (YQJPXY)	Traditional Chinese herbal formula	Cells:HK-2, TCMK1; Animals:C57BL/6 mice	Gastric lavage	Acute Kidney Injury (AKI)	Reduce tissue damage, inflammation, and fibrosis in the kidneys, and inhibit ferroptosis in renal tubular epithelial cells	By inhibiting STING protein expression and its interaction with NCOA4, STING-NCOA4-mediated ferritin phagocytosis is blocked, thereby reducing the release of free iron and lipid peroxidation, inhibiting ferroptosis, and protecting renal function	[164]	Indirect pathway association
	Nephropathy II Decoction (NED)	Traditional Chinese herbal formula	Cells:TCMK1,HK-2; Animals:C57BL/6J mice	Gastric lavage	Renal fibrosis	Improves kidney function, reduces pathological damage to the kidneys and collagen deposition, and inhibits renal tubular cell apoptosis	By improving mitochondrial oxidative phosphorylation, maintaining mitochondrial homeostasis, and inhibiting mtDNA leakage, it reduces the activation of the cGAS-STING signaling pathway. Its active ingredient binds directly to STING and inhibits its activity, thereby further blocking the downstream TBK1/IRF3/IFN-β pathway	[98]	Direct target verification
	Zhen Wu Decoction (ZWD)	Traditional Chinese herbal formula	Cells:HK-2, HEK293T; Animals:C57BL/6J mice	Gastric lavage	Renal fibrosis	Reduces renal interstitial fibrosis, improves kidney function, and protects mitochondrial structure and function	By activating NRF2, it upregulates the expression of mitochondrial transcription factor A (TFAM), maintains mitochondrial membrane integrity and biosynthesis, thereby reducing the leakage of mitochondrial DNA (mtDNA) into the cytoplasm, inhibiting the abnormal activation of the STING pathway, and alleviating inflammation and fibrosis	[165]	Indirect pathway association
	Qihuang Yishen formula (QHYS)	Traditional Chinese herbal formula	Cells:HK-2; Animals:KK-Ay mice	Gastric lavage	Diabetic Kidney Disease (DKD)	Reduce proteinuria, improve kidney function, and alleviate renal tissue damage and tubular inflammation	By protecting mitochondrial structure and function, it reduces HG/PA-induced mtDNA leakage, thereby inhibiting the activation of the cGAS-STING pathway and the production of downstream inflammatory factors	[166]	Indirect pathway association
	Huangqi-Danshen decoction (HDD)	Astragalus, Salvia	Cells:Primary mouse proximal renal tubular epithelial cells; Animals:C57BL/6 mice	Gastric lavage	Renal fibrosis	Improves kidney function, reduces pathological damage to the kidneys and collagen deposition, and alleviates renal fibrosis	By upregulating the expression of stearoyl-CoA desaturase 1 (SCD1), renal lipid composition is modulated, thereby inhibiting the activation of the cGAS-STING signaling pathway and reducing the production of downstream inflammatory factors	[167]	Indirect pathway association
Autoimmune and Inflammatory Diseases	Macrophage targeted triptolide micelles(FDL@TP)	Triptolide is derived from Tripterygium wilfordii	Cells:RAW264. 7;Animals:ICRmice	intravenous injection	Rheumatoid Arthritis (RA)	Targets M1 macrophages, inhibits the secretion of inflammatory factors, reduces joint swelling and bone destruction, and reduces the systemic toxicity of triptolide	1.Targeted delivery: Folic acid-modified micelles recognize the folate receptor β overexpressed on M1 macrophages, thereby achieving targeted accumulation at joint sites and enhanced cellular uptake. 2. Inhibition of the cGAS-STING pathway: The released triptolepine downregulates the expression of cGAS and STING proteins in macrophages, thereby inhibiting the release of downstream inflammatory cytokines such as TNF-α, IL-1β, and IL-6	[193]	Indirect pathway association
	Baitong Decoction (BTD)	Traditional Chinese herbal formula	C57BL/6 mice	Gastric lavage	Ulcerative colitis (UC)	Alleviates DSS-induced weight loss, elevated DAI, and colonic shortening; improves colonic pathological damage and goblet cell loss; and reduces levels of inflammatory factors and myeloperoxidase	By inhibiting the STING signaling pathway and the activation of its downstream NF-κB, while simultaneously inhibiting the JAK/STAT signaling pathway, this approach alleviates the intestinal inflammatory response	[168]	Indirect pathway association
	PC-FA nanoparticles	Ferulic acid is derived from various plants; pectin is derived from citrus fruits	Cells:NCM460; Animals:Kunming mice, SD rats	Oral、Gastric lavage	Ulcerative colitis (UC)	Alleviates DSS-induced weight loss, increased disease activity index, and colonic shortening; improves colonic pathological damage; and reduces levels of inflammatory cytokines	By targeting ferulic acid to the colon, its bioavailability is enhanced. The nanoparticles scavenge ROS and mitigate DNA damage (reducing γH2AX), thereby inhibiting the activation of the cGAS-STING pathway and reducing the production of downstream inflammatory factors (TNF-α, IL-1β)	[194]	Indirect pathway association
	American Ginseng & Achyranthes (AG&A)	Pairs of Traditional Chinese Medicines	Animals:NOD mice	Gastric lavage	Primary Sjögren's Syndrome (PSS)	Increase saliva flow, alleviate pathological damage to the submandibular glands, and reduce levels of inflammatory factors	By reducing mitochondrial dysfunction and inhibiting mtDNA leakage, this approach downregulates the expression of proteins associated with the cGAS-STING pathway, thereby attenuating the type I interferon-mediated inflammatory response	[169]	Indirect pathway association
	Maidong Dishao Decoction (MDDST)	Traditional Chinese herbal formula	Cells:A253, Primary submandibular gland epithelial cells from mice; Animals:NOD/Ltj mice, ICR mice	Gastric lavage	Sjögren's syndrome (Sjögren's syndrome)	Reduced water intake in NOD mice, increased salivary flow rate, decreased serum SSA and IgG levels, and alleviated pathological damage to the submandibular glands	By reducing the accumulation of reactive oxygen species (ROS), maintaining mitochondrial membrane potential and mitochondrial homeostasis, it inhibits excessive mitochondrial autophagy. By maintaining mitochondrial homeostasis and reducing mtDNA leakage, it further inhibits the activation of the cGAS-STING signaling pathway, thereby alleviating downstream inflammation	[170]	Indirect pathway association
	Compound Danshen Dripping Pill (CDDP)	Traditional Chinese herbal formula	Cells:BMDMs, THP-1, HEK293T, L929; Animals:C57BL/6J mice	Gastric lavage	Trex1 deficiency and obesity-induced insulin resistance	Reduces multi-organ inflammation in mice and improves insulin resistance and glucose tolerance in obese mice	It specifically inhibits the cGAS-STING signaling pathway. Mechanistically, it does not affect STING oligomerization but instead disrupts the formation of the STING signaling complex by blocking the interaction between STING and TBK1, thereby inhibiting the activation of downstream IRF3 and NF-κB as well as the production of type I interferons and inflammatory cytokines	[171]	Indirect pathway association
	Shaoyao Gancao decoction (SGD)	Traditional Chinese herbal formula	Cells:Hepatic stellate cells (HSCs), splenic monocytes; Animals:C57BL/6 mice	Gastric lavage	Autoimmune hepatitis (AIH)	Reduces pathological damage to the liver and spleen, improves liver function	By promoting splenic immune homeostasis, it inhibits the activation of the cGAS-STING signaling pathway in the liver, as well as the downstream activation of NF-κB and TGF-β1, thereby ultimately suppressing the activation and proliferation of hepatic stellate cells (HSCs) and delaying the progression of liver fibrosis	[73]	Indirect pathway association
	Zixue Powder (ZXP)	Traditional Chinese herbal formula	Animals:SD rat	Gastric lavage	Sepsis-associated thrombosis	Reduces the formation of microthrombi in the liver, improves thrombocytopenia, and lowers levels of coagulation-related factors	By inhibiting the platelet STING signaling pathway, it reduces STING-mediated SNARE complex assembly, thereby suppressing platelet P-selectin secretion, which in turn reduces the formation of neutrophil extracellular traps (NETs) and ultimately resolves thrombi. The STING agonist DMXAA can reverse the protective effects of ZXP	[172]	Indirect pathway association
	Fufang Epimedium formula (FEF)	Traditional Chinese herbal formula	Animals:C57BL/6J mice	Gastric lavage	Treatment of Acute Liver Injury (ALI)	Reduces transaminase levels, alleviates oxidative stress and inflammation, inhibits hepatocyte apoptosis, and improves liver pathology	1. Activates the PI3K/AKT/Bcl-2 pathway, promotes the phosphorylation of PI3K and AKT, upregulates the Bcl-2/Bax ratio, and inhibits caspase-3 and PARP cleavage, thereby exerting an anti-apoptotic effect. Inhibits the cGAS-STING-IRF3 pathway, downregulates the expression of cGAS and STING, and inhibits the phosphorylation of TBK1 and IRF3, thereby reducing the production of inflammatory cytokines	[173]	Indirect pathway association
Digestive System Disorders	ErTao decoction (ETD)	Traditional Chinese herbal formula	Cells:RAW264. 7, HSC-T6; Animals:C57BL/6 mice	Gastric lavage	Liver fibrosis (LF)	Alleviates CCl₄-induced liver damage and collagen deposition, improves liver function, and inhibits hepatic stellate cell (HSC) activation	By inhibiting the activation of the STING signaling pathway in macrophages, it reduces M1 macrophage polarization while simultaneously suppressing NLRP3 inflammasome activation	[117]	Indirect pathway association
	Lingguizhugan decoction (LGZG)	Traditional Chinese herbal formula	Cells:BMDMs, Primary hepatic macrophages, AML-12, primary hepatocytes; Animals:C57BL/6J mice	Gastric lavage	Non-alcoholic fatty liver disease (NAFLD)	Alleviates HFD-induced hepatic lipid accumulation, liver damage, and oxidative stress, and improves insulin resistance	By inhibiting the activation of the STING-TBK1-NF-κB signaling pathway in hepatic macrophages, the release of inflammatory factors is reduced, thereby alleviating macrophage-mediated lipid deposition in hepatocytes. The active ingredient can inhibit the activation of the STING pathway	[35]	Indirect pathway association
Cardiovascular disease	Tanshinone IIA (Ta-IIA) & Astragaloside IV (As-IV) Joint Application	Ta-IIA is derived from Salvia miltiorrhiza; As-IV is derived from Astragalus membranaceus	Cells:HL1; Animals:C57BL/6 mice	Gastric lavage	Myocardial ischemia–reperfusion injury (MIRI)	Reduce the area of myocardial infarction, lower cardiac enzyme levels, improve cardiac function, and alleviate myocardial cell apoptosis, oxidative stress, and inflammation	The combined use of these two agents is more effective than either alone in inhibiting STING phosphorylation and the subsequent phosphorylation of TBK1 and IRF3, thereby blocking the activation of the STING signaling pathway and exerting anti-apoptotic, antioxidant, and anti-inflammatory effects	[185]	Indirect pathway association
	Xin-Ji-Er-Kang (XJEK)	Traditional Chinese herbal formula	Cells:AC16;Animals:C57BL/6 mice	Gastric lavage	Chronic heart failure (CHF)	Reduce myocardial hypertrophy, collagen deposition, and ventricular remodeling; improve cardiac function; and lower serum NT-proBNP and cTnI levels	By promoting the nuclear translocation of the nuclear receptor NR3C1, it enhances its binding to the promoter of mitochondrial fusion protein 2 (MFN2), thereby upregulating MFN2 transcription and expression. MFN2 restores mitochondrial homeostasis and reduces mtDNA leakage, thereby inhibiting the activation of the cGAS-STING signaling pathway and downstream inflammatory responses	[133]	Indirect pathway association
	Tao Hong Si Wu decoction (TSD)	Traditional Chinese herbal formula	Cells:HL-1;Animals:db/db mice	Gastric lavage	Diabetic Cardiomyopathy (DCM)	Improves cardiac structure and function, reduces myocardial fibrosis and mitochondrial damage, and lowers levels of oxidative stress and inflammation	Reducing pyroptosis in cardiomyocytes by inhibiting the cGAS-STING signaling pathway and the activation of the downstream NLRP3 inflammasome	[174]	Indirect pathway association
	Kuanxiong Aerosol (KX)	Traditional Chinese herbal formula	Cells:THP-1;Animals:ApoE^−/−^ mice	Gastric lavage	Atherosclerosis (AS)	Inhibits the progression of AS and promotes the regression of existing plaques, reduces plaque inflammation, and improves vascular function	By inhibiting the cGAS-STING-P65 signaling pathway, it reduces the differentiation and apoptosis of the CX3CR1 + pro-inflammatory macrophage subset and attenuates the inflammatory response within plaques	[175]	Indirect pathway association
	Gualou-Xiebai herb pair (GLXB)	Pairs of Traditional Chinese Medicines	Cells:HUVEC; Animals:ApoE^−/−^mice	Gastric lavage	Atherosclerosis (AS)	Reduce the area of aortic plaques, alleviate endothelial cell damage and inflammatory responses, and inhibit ferroptosis	By inhibiting the scavenger receptor LOX-1, the activation of the cGAS-STING signaling pathway it mediates is blocked, thereby downregulating NCOA4 expression, inhibiting NCOA4-mediated ferritin phagocytosis, reducing the release of free iron and lipid peroxidation, and ultimately suppressing ferroptosis in endothelial cells	[176]	Indirect pathway association
Neurodegenerative Diseases and Nervous System Injuries	Dingzhen Pills (DZP)	Traditional Chinese herbal formula	SH-SY5Y Cells; C57BL/6J mice	Gastric lavage	Parkinson's disease (PD)	Improves motor dysfunction; protects dopaminergic neurons in the substantia nigra; inhibits neuroinflammation and ferroptosis	By targeting multiple sites through various active ingredients, it inhibits the cGAS-STING pathway, thereby suppressing neuronal ferroptosis and neuroinflammation	[177]	Indirect pathway association
	Bu Yang Huan Wu Decoction (BYHWD)	Traditional Chinese herbal formula	BV2 microglia with OGD injury; (2VO)SD rats	Gastric lavage	Vascular cognitive impairment	Improves learning and memory function; increases cerebral blood flow; reduces neuronal and white matter damage; inhibits neuroinflammation	Inhibiting p53 and restoring TREX1 expression thereby suppresses neuroinflammation mediated by the cGAS-STING pathway	[178]	Indirect pathway association
	ZhiZiHouPo Decoction(ZZHP)	Traditional Chinese herbal formula	PC12 cells with CORT-induced damage; C57BL/6J mice	Gastric lavage	Depression	Improves depression-like behavior; reduces damage to hippocampal neurons and oxidative stress; inhibits neuroinflammation	Upregulates neuronal TFAM, enhances TFAM-LC3-mediated mitochondrial autophagy, clears cytoplasmic mtDNA, and thereby inhibits the cGAS-STING inflammatory pathway	[179]	Indirect pathway association
	Jiao Tai Wan (JTW)	Traditional Chinese herbal formula	LPS-stimulated BV2 microglia; C57BL/6J mice	Gastric lavage	Depression	Improves depression-like behavior; reduces damage to hippocampal neurons; inhibits neuroinflammation	Inhibiting the STING pathway in microglia reduces M1 polarization and the release of pro-inflammatory cytokines, thereby alleviating neuroinflammation	[180]	Indirect pathway association
Other diseases	JingTangTiaoZhiFormula(JTTZF)	Traditional Chinese herbal formula	HepG2Cells; C57BL/6 mice and zebrafish models;	Gastric lavage	Obesity-related type 2 diabetes (T2D)	Improve glucose and lipid metabolism; reduce insulin resistance; reduce hepatic steatosis and inflammation	Inhibiting the cGAS-STING/TBK1/NF-κB signaling pathway in the liver to reduce inflammatory responses and lipid deposition	[181]	Indirect pathway association
	Naringenin (NRN)-loaded SBMA/GelMA hydrogels	Nanoparticle formulations	macrophage; BMDMs C57BL/6 mice;	Local application or implantation	Diabetic wounds	Promotes wound healing in diabetes; Regulates immune balance; Promotes M2 macrophage polarization and angiogenesis	Activates PPARα, inhibits the STING pathway, thereby promoting M2 macrophage polarization, reducing inflammation, and promoting angiogenesis	[195]	Indirect pathway association
	Sanhuang Decoction (SHD)	Traditional Chinese herbal formula	HUVECs; C57BL/6 mice	For topical use	Diabetic Foot Ulcers (DFUs)	Promotes wound healing in diabetes; reduces high-glucose-induced apoptosis and senescence in endothelial cells; promotes angiogenesis	Inhibits the STING/TBK1/IRF3 signaling pathway in endothelial cells, reduces inflammation, and promotes cell proliferation and angiogenesis	[182]	Indirect pathway association
	WenShenTongLuoZhiTongDecoction (WSTLZTD)	Traditional Chinese herbal formula	Bone marrow-derived macrophages (BMDMs); BMSCs; C57BL/6J mice	Gastric lavage	Senile Osteoporosis (SOP)	Reduce bone loss; Inhibit macrophage senescence; Promote osteogenic differentiation of BMSCs	Upregulates LONP1 in macrophages, thereby inhibiting their senescence; consequently, it suppresses the activation of the cGAS/STING pathway in BMSCs and promotes their osteogenic differentiation	[36]	Indirect pathway association

### The relationship between Traditional Chinese Medicine as a complex system and the STING pathway

TCM has a long history of application in the prevention and treatment of immune-related diseases. Its holistic concepts of “reinforcing healthy qi and eliminating pathogenic factors” and “maintaining the balance of yin and yang” are highly consistent with the modern immunological concept of preserving immune homeostasis. With the growing recognition of the central role of the cGAS-STING signaling pathway in innate immune regulation, accumulating evidence has demonstrated that TCM herbal formulas, natural extracts, and their bioactive constituents can modulate this pathway and thereby participate in the dynamic regulation of immune responses. Depending on the pathological context, TCM may either activate cGAS-STING signaling to enhance antitumor and antimicrobial immunity or suppress its aberrant activation to alleviate inflammation, tissue injury, and immune dysregulation. These findings not only provide new mechanistic insights into the multi-component and multi-target characteristics of TCM, but also offer valuable perspectives for the development of novel therapeutic strategies targeting the cGAS-STING pathway.

Emerging evidence indicates that the cGAS-STING pathway plays a pivotal role in a broad spectrum of pathological conditions, including cancer, infectious diseases, autoimmune disorders, tissue fibrosis, metabolic diseases, and neurodegenerative diseases. However, the biological functions of this pathway are highly disease-dependent. In cancer and infectious diseases, appropriate activation of STING signaling enhances host defense by promoting both innate and adaptive immune responses. In contrast, persistent or excessive STING activation in inflammatory, autoimmune, and fibrotic diseases frequently exacerbates tissue injury and disease progression. Therefore, the regulation of the cGAS-STING pathway by TCM exhibits a distinctive feature of bidirectional immunomodulation, enabling context-dependent adjustment of pathway activity to restore immune homeostasis and achieve therapeutic benefits. This feature is highly consistent with the three advantages of TCM in tumor prevention and treatment, namely “reinforcing healthy qi and eliminating pathogenic factors, reducing toxicity and enhancing efficacy, and holistic regulation” [152].

**Fig. 4:**
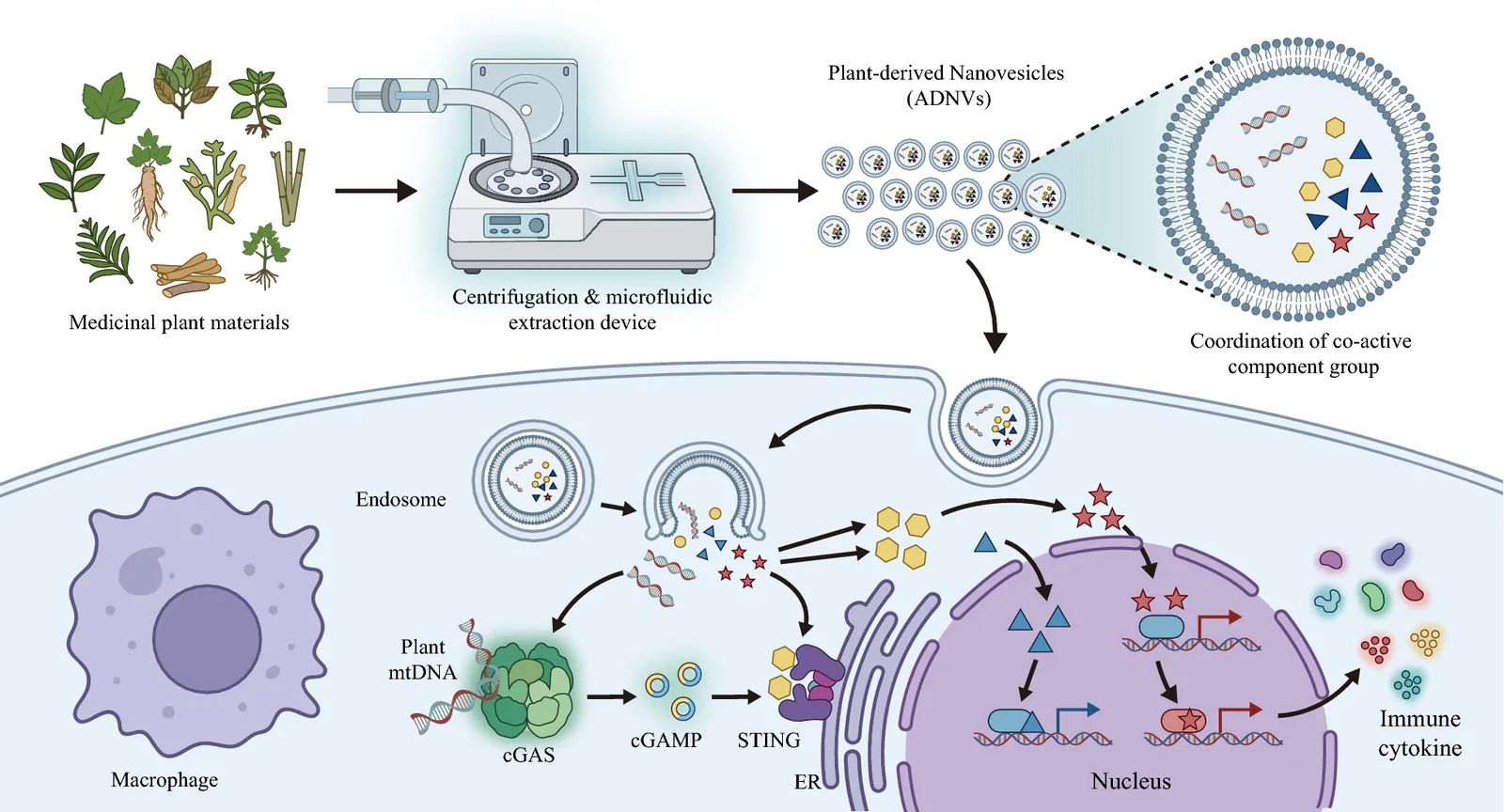
Schematic illustration of a TCM complex structurally based on plant-derived nanovesicles in the regulation of the cGAS-STING pathway

According to the mode of intervention, current studies can be broadly categorized into three groups: TCM compound preparations, TCM extracts, and TCM nanomedicines. TCM compound preparations reflect the characteristics of multi-component synergy and holistic regulation; TCM extracts focus on defined groups of bioactive constituents; whereas TCM nanomedicines integrate modern drug-delivery technologies to improve bioavailability, tissue targeting, and therapeutic efficacy (Fig. 4).

Compared with single compounds, TCM complexes better embody the theoretical features of TCM, including syndrome differentiation, holistic regulation, and the compatibility principle of “monarch, minister, assistant, and guide.” The monarch–minister–assistant–guide principle may also provide a hierarchical framework for interpreting how compound prescriptions regulate the cGAS–STING pathway. The monarch herb is intended to address the principal pathological process and may provide the major constituents responsible for regulating upstream cytosolic DNA accumulation, mitochondrial injury, or the core cGAS–STING signaling axis. Minister herbs may reinforce the principal effect by acting on complementary pathway nodes or by regulating associated processes such as oxidative stress, autophagy, NF-κB signaling, inflammasome activation, and immune-cell function. Assistant herbs may either strengthen the therapeutic effect or restrict excessive inflammatory activation and treatment-related toxicity, which is particularly relevant because both excessive activation and excessive inhibition of cGAS–STING signaling may be detrimental. Guide herbs may contribute by harmonizing the formula or modifying the dissolution, absorption, metabolism, and tissue distribution of other constituents. However, these traditional compatibility roles should not be regarded as direct evidence that individual herbs or compounds engage specific molecular targets. Their respective contributions to cGAS–STING regulation require validation through chemical characterization, herb-subtraction, constituent-removal and add-back experiments, pharmacokinetic studies, and pathway-specific functional assays. These concepts are reflected mechanistically in the coordinated regulation of multiple upstream danger signals, core STING activation or inhibition, and downstream inflammatory or interferon responses. Because TCM complexes contain multiple constituents, evidence from formulas, extracts, and nanomedicines should be interpreted with particular caution. Changes in cGAS-STING-related markers may reflect the combined effects of multiple components rather than direct regulation by a single active molecule. Therefore, studies on TCM complexes require stricter validation of active constituents, target engagement, and causal pathway involvement.

This figure depicts the formation of a TCM complex and its potential mechanism in regulating the cGAS-STING pathway. Plant-derived nanovesicles from medicinal plants may serve as natural composite carriers that co-encapsulate plant mitochondrial DNA (mtDNA), lipids, proteins, and small bioactive molecules, thereby forming a TCM complex with structural integration and functional synergy. After uptake by macrophages, the nucleic acid components carried by the complex can be recognized by cGAS and activate STING signaling, while other co-delivered active constituents may further synergistically modulate downstream transcriptional activation and immune factor release. This process suggests that the TCM complex is not a simple additive combination of individual components, but rather functions through the co-existence, co-delivery, and coordinated regulation of multiple constituents, providing a new mechanistic framework for understanding the holistic effects of multicomponent traditional Chinese medicine.

#### Traditional Chinese Medicine compound preparations

TCM compound preparations include classical prescriptions, modern compound formulas, injections, capsules, pills, aerosols, and other clinically used dosage forms. Owing to their multi-component composition and formula-based compatibility, these preparations possess unique advantages in regulating the STING pathway through multi-target and multi-level interventions [153].

Similarly, Qianlie Xiaozheng Formula (QLXZF) inhibits CHK1 expression, induces DNA damage accumulation, activates the STING/TBK1/IRF3 pathway, and promotes the production of chemokines such as CCL5 and CXCL10, which recruit CD8^+^ T cells and inhibit prostate cancer progression [154]. Similarly, the Feiwei Mixture (FWHJ), a hospital-prepared traditional Chinese medicine formulation, inhibits the growth of NSCLC by upregulating NR1D1 expression, activating the cGAS-STING pathway, and inhibiting the JAK-STAT3 signaling axis. This promotes the production and secretion of chemokines and enhances T-cell infiltration [155].

In addition to antitumor activity, activation of the STING pathway by TCM formulas has also been implicated in antiviral immunity. Studies have shown that luteolin (Lut) and schisandrin C (Sc), two active constituents of Liuwei Wuling Tablets, inhibit hepatitis B virus (HBV) replication through activation of the cGAS-STING pathway in macrophages [156]. These findings exemplify the synergistic and complementary characteristics of TCM and provide new insights into the prevention of HBV-related hepatocellular carcinoma.

Collectively, these studies indicate that, despite variations in the composition and functional focus of TCM formulas, most ultimately activate the STING pathway. They achieve this through the regulation of either a single pathway or a multi-node regulatory network, thereby promoting CD8+ T-cell infiltration and further enhancing antitumor immunity. Consistent with its name and traditional use, Bazi Bushen Capsules reflect the TCM principle of tonifying the kidney and strengthening healthy qi; the activation of macrophage cGAS–STING signaling may explain how such tonifying therapy enhances antitumor immune surveillance.

In respiratory diseases such as acute lung injury (ALI) and pulmonary fibrosis, excessive activation of the STING pathway contributes to uncontrolled inflammatory responses and progressive tissue damage. Consequently, inhibition of this pathway has emerged as an important mechanism underlying the therapeutic effects of several TCM herbal formulas.

Yao et al. reported that Shuangdan Jiedu Decoction (SJD), a multi-herb formula composed of medicinal plants including *Lonicera japonica* and *Forsythia suspensa*, markedly attenuated lipopolysaccharide (LPS)-induced ALI. SJD not only suppressed aberrant activation of the cGAS-STING pathway but also inhibited multiple inflammasome pathways, including NLRP3, AIM2, and NLRC4. Notably, at equivalent doses, individual herbs showed no significant inhibitory effects on these pathways, whereas the full six-herb combination produced a pronounced effect, suggesting that SJD exerts its anti-inflammatory activity through synergistic interactions among multiple herbal components [157]. This synergistic inhibition of cGAS-STING and inflammasome pathways is consistent with the heat-clearing and detoxifying rationale of SJD, emphasizing the TCM concept that combined herbs may remove excessive pathogenic factors more effectively than single herbs.

Yinqiao Powder (YQP) a classical prescription widely used for respiratory infections, also exhibits protective effects against LPS-induced ALI. YQP significantly alleviates pulmonary pathological injury, pulmonary edema, and inflammatory cytokine production. Unlike SJD, however, YQP does not interfere with STING oligomerization or STING–TBK1 complex formation. Instead, it selectively targets the TBK1–IRF3 signaling axis by inhibiting IRF3 phosphorylation and nuclear translocation, thereby reducing type I interferon production and downstream inflammatory responses [158]. These findings indicate that TCM formulas may achieve precise immunomodulation by selectively targeting distinct nodes within the STING signaling network.

Pulmonary fibrosis is a progressive interstitial lung disease. Studies investigating Tanreqing Injection (TRQ) have shown protective effects against bleomycin-induced pulmonary fibrosis. TRQ inhibits STING expression and suppresses endoplasmic reticulum stress (ERS)-related signaling, downregulating BIP, p-PERK, p-eIF2α, and ATF4, while also inhibiting NF-κB p65 phosphorylation. These effects alleviate inflammatory responses and fibrotic progression, significantly reducing lung tissue damage, collagen deposition, and inflammatory cell infiltration, thereby improving pulmonary function [159].

Similarly, Qingfei Xieding prescription (QF) significantly reduces lung injury, collagen deposition, inflammatory infiltration, and mortality in a bleomycin-induced pulmonary fibrosis mouse model. Its therapeutic effects are closely associated with autophagy activation. By enhancing autophagic activity, QF alleviates mitochondrial damage and reduces mtDNA release, thereby suppressing activation of the mtDNA–cGAS-STING pathway and downstream NF-κB p65 nuclear translocation and inflammatory factor expression. Importantly, the autophagy inhibitor chloroquine reverses the protective effects of QF, confirming that autophagy is a key mechanism underlying its therapeutic action [160].

Bu-fei Decoction (BFD) has also shown therapeutic potential in age-related chronic obstructive pulmonary disease (COPD). Using network pharmacology, the study predicted that several active components in BFD may directly target cGAS. It was also found that in CD4⁺ naive T cells from elderly COPD patients and animal models, the mitochondrial protein C1qbp was downregulated, triggering activation of the cGAS-STING pathway and increased markers of cellular senescence. Interestingly, downregulation of C1qbp can spontaneously activate the cGAS-STING pathway in Jurkat T cells, leading to cellular senescence. However, BFD treatment upregulates C1qbp expression, acting at the source of the pathway to inhibit cGAS-STING activation, reduce the expression of senescence-associated and inflammatory markers, restore the number of CD4⁺ naive T cells, and improve lung function [161]. However, the direct interaction between the predicted BFD components and cGAS still requires experimental confirmation. Therefore, the network pharmacology findings should be interpreted as predicted pathway involvement, whereas the cell and animal experiments provide indirect support for cGAS-STING pathway modulation.

Taken together, TCM herbal formulas regulate the STING pathway at multiple levels in respiratory diseases. Although different formulas target distinct molecular events according to disease-specific pathological characteristics, most ultimately exert their therapeutic effects by suppressing aberrant STING activation. These findings highlight the holistic therapeutic philosophy of TCM, whereby coordinated interactions among multiple herbal constituents converge on common pathogenic pathways. Future studies integrating transcriptomics, proteomics, metabolomics, and network pharmacology may facilitate systematic characterization of the “component–target–pathway” regulatory networks underlying TCM formulas. Furthermore, patient stratification based on STING pathway activation profiles may provide a scientific basis for precision medicine approaches in integrative Chinese and Western medicine.

Cisplatin-induced acute kidney injury (CIAKI) is a common dose-limiting adverse effect of chemotherapy and is frequently associated with mitochondrial DNA (mtDNA) leakage and subsequent activation of the cGAS-STING signaling pathway. Increasing evidence suggests that several TCM herbal formulas exert renoprotective effects by targeting this pathway and its upstream mitochondrial regulatory mechanisms.

Yi-Shen-Xie-Zhuo formula (YSXZF) has been reported to protect against CIAKI by suppressing cGAS-STING-mediated inflammatory responses and apoptosis. Both in vitro and in vivo studies demonstrated that YSXZF significantly reduced serum creatinine and blood urea nitrogen levels, alleviated renal tubular injury, and downregulated the mRNA and protein expression of cGAS and STING. Furthermore, treatment with YSXZF markedly decreased the phosphorylation levels of TBK1 and IRF3, indicating effective suppression of downstream STING signaling [162].

Similarly, studies indicate that Shenqi Fuzheng Injection (SQFZ) has been shown to reverse cisplatin-induced activation of the cGAS–STING signaling pathway while simultaneously ameliorating oxidative stress and mtDNA damage. Network pharmacology analysis identified apoptosis-related, oxidative stress-related, and interferon-related pathways as potential therapeutic targets of SQFZ. Experimental studies further demonstrated that co-administration of SQFZ with cisplatin alleviated renal tubular injury and apoptosis without compromising the antitumor efficacy of cisplatin. Mechanistically, these protective effects were attributed to improved mitochondrial function, reduced mtDNA leakage, inhibition of cGAS–STING activation, and decreased production of downstream inflammatory mediators [163].

The Yi-Qi-Jian-Pi-Xiao-Yu Formula (YQJPXY), a modified prescription derived from Buyang Huanwu Decoction, contains multiple medicinal herbs including *Astragalus membranaceus* and *Rheum palmatum*. Network pharmacology analysis identified ferroptosis as a major regulatory process involved in its therapeutic activity. Subsequent investigations revealed that YQJPXY promotes STING ubiquitination and degradation, thereby disrupting the interaction between STING and nuclear receptor coactivator 4 (NCOA4). This effect inhibits STING–NCOA4-mediated ferritinophagy, reduces intracellular iron release and lipid peroxidation, and ultimately suppresses ferroptotic cell death. Notably, pharmacological inhibition of STING reproduced the protective effects of YQJPXY, whereas administration of the STING agonist DMXAA exacerbated renal injury, further confirming the central role of STING in ferroptosis regulation [164].

Nephropathy II Decoction (NED) protects mitochondrial function by specifically inhibiting the cGAS-STING pathway, alleviating renal fibrosis in various mouse models of chronic kidney disease. Its mechanism involves maintaining mitochondrial oxidative phosphorylation, reducing mtDNA leakage and apoptosis, thus inhibiting STING pathway activation. In models of folic acid-induced nephropathy, unilateral ureteral obstruction, and bilateral renal ischemia–reperfusion injury, NED significantly improved renal function and reduced pathological damage [98].

Zhen Wu Decoction (ZWD) also inhibits abnormal STING pathway activation by activating NrF2, promoting mitochondrial transcription factor A (TFAM) expression, maintaining mitochondrial membrane integrity, and reducing mtDNA release into the cytoplasm. The NRF2 inhibitor ML385 attenuates the protective effects of ZWD [165].

Similarly, the Qihuang Yishen formula (QHYS) alleviates mitochondrial structural damage, upregulates TFAM and TOM20 expression, and reduces HG/PA-induced mtDNA leakage. It inhibits cGAS-STING pathway activation and the production of downstream inflammatory factors, thereby improving tubular damage in diabetic nephropathy [166]. These studies collectively indicate that mitochondrial quality control represents a major therapeutic target of TCM interventions. From the perspective of TCM theory, this is consistent with the principle of treating the root cause of disease and supporting organ function, such as tonifying the kidney, strengthening the spleen, removing dampness, and resolving stasis in chronic renal disorders.

In addition to the above formulas, TCM herbal combinations may also provide therapeutic benefits in urinary system disorders through metabolic regulation. Huangqi-Danshen Decoction (HDD) regulates lipid metabolism by upregulating stearoyl-CoA desaturase 1 (SCD1), thereby inhibiting the cGAS/STING signaling pathway and alleviating renal fibrosis. The active components in HDD-isopapaverine, baicalin, and danshenone-can reverse cGAS-STING activation induced by SCD1 inhibition, providing theoretical support for the synergistic effects of the compound's multiple components [167].

Although these studies differ in their therapeutic strategies and molecular targets, most converge on a common pathogenic axis involving mitochondrial dysfunction, mtDNA leakage, and STING activation. This observation suggests that the mitochondrial–STING pathway may represent a shared downstream mechanism underlying diverse kidney diseases and a promising therapeutic target for TCM-based interventions.

Inflammatory bowel disease (IBD), particularly ulcerative colitis, is a chronic, recurrent inflammatory condition of the intestines. Studies have shown that Bai Tong Tang (BTD) has therapeutic effects on dextran sulfate sodium (DSS)-induced colitis. Network pharmacology predicts that its mechanism involves multiple pathways, including TNF, JAK/STAT, and STING. These network pharmacology results should be considered hypothesis-generating. The subsequent animal experiments support an association between BTD treatment and reduced STING/JAK-STAT pathway activation, but they do not by themselves prove direct target engagement. In a mouse model of colitis, BTD dose-dependently alleviated weight loss, elevated disease activity indices, and colonic shortening. It also improved colonic histopathological damage, goblet cell loss, and reduced serum inflammatory factor and myeloperoxidase levels. The results indicate that BTD exerts its anti-inflammatory effects by simultaneously regulating the STING and JAK/STAT pathways [168].

Sjögren’s syndrome is a chronic autoimmune disorder primarily characterized by xerostomia and xerophthalmia. Emerging evidence suggests that mitochondrial dysfunction in salivary gland epithelial cells and aberrant activation of the cGAS–STING signaling axis are critically involved in its pathogenesis. Through integrated network pharmacology, molecular docking, and in vivo validation, Li et al. systematically elucidated the therapeutic mechanism of the American ginseng–Achyranthes root (AG&A) combination in Sjögren’s syndrome. In NOD mouse models, AG&A significantly enhanced salivary secretion, alleviated mitochondrial ultrastructural damage in submandibular glands, and reduced cytosolic mtDNA release. Concurrently, it suppressed the expression of cGAS–STING pathway components, including cGAS, STING, and phosphorylated STING, indicating that its anti-inflammatory effects are mediated through inhibition of the mtDNA–cGAS–STING axis [169].

Similarly, Maidong Dishao Decoction (MDDST) has demonstrated therapeutic efficacy in Sjögren’s syndrome. In NOD mice, MDDST administration significantly reduced water intake, restored salivary flow, decreased serum SSA and IgG levels, and ameliorated histopathological damage in submandibular glands. Mechanistically, MDDST attenuates excessive mitophagy and suppresses cGAS–STING pathway activation by reducing ROS accumulation in salivary epithelial cells, thereby preserving mitochondrial membrane potential and maintaining mitochondrial homeostasis. Notably, pharmacological inhibition of mitophagy partially abolished the protective effects of MDDST, whereas ROS scavenging recapitulated its efficacy, further substantiating a ROS–mitochondria–cGAS/STING regulatory axis [170].

Compound Danshen Dripping Pills (CDDP) have been identified as functional inhibitors of the cGAS–STING pathway. In macrophages stimulated with STING agonists, CDDP dose-dependently suppressed STING and IRF3 phosphorylation, thereby reducing downstream IFN-β and ISG expression, while sparing the RIG-I pathway. Mechanistically, CDDP does not interfere with STING oligomerization but disrupts STING–TBK1 complex assembly. In Trex1-deficient mice, CDDP markedly attenuated systemic inflammatory responses across multiple organs. In diet-induced obese models, it further improved insulin sensitivity and glucose tolerance, underscoring its pleiotropic regulatory effects [171]. From the perspective of formula compatibility, Salvia miltiorrhiza is generally regarded as the monarch herb in CDDP, Panax notoginseng serves as the minister herb, and borneol performs assistant and guide functions. The lipophilic tanshinones and hydrophilic phenolic acids derived from Salvia miltiorrhiza are likely to constitute the principal pharmacological basis of the formula and may contribute to the observed inhibition of STING–TBK1 complex assembly and downstream inflammatory signaling. Notoginseng saponins may complement this effect by regulating macrophage activation, mitochondrial homeostasis, metabolic inflammation, and related immune pathways, thereby reinforcing the overall inhibition of pathological cGAS–STING signaling. Borneol is less likely to represent a major direct regulator of cGAS or STING; its more plausible contribution is to facilitate the dissolution, membrane transport, and tissue exposure of co-administered constituents and to coordinate the overall action of the formula.

Nevertheless, the existing study examined CDDP as an intact preparation and did not determine which herb or constituent was responsible for disrupting STING–TBK1 assembly. It also remains unclear whether the observed activity is dominated by one constituent, produced by several co-active constituents, or enhanced by pharmacokinetic interactions among the three components. Thus, the current evidence supports inhibition of cGAS–STING-related signaling at the formula level, whereas the molecular correspondence between the monarch, minister, assistant, and guide components and individual pathway nodes remains to be experimentally established.

Through integrated network pharmacology and experimental validation, Shaoyao Gancao Decoction (SGD) was shown to attenuate liver fibrosis in autoimmune hepatitis via regulation of the cGAS–STING pathway along the spleen–liver axis. IL-1β was identified as a key target, with molecular docking confirming stable binding of active compounds to IL-1β/IL-12. In vivo studies demonstrated that SGD significantly ameliorates liver and spleen pathology and improves liver function. This effect is mediated by restoring splenic immune homeostasis and suppressing hepatic expression of cGAS, STING, NF-κB, and TGF-β1, thereby inhibiting hepatic stellate cell activation and proliferation [73].

Zixue San (ZXP), a classical prescription traditionally used for febrile encephalopathy and pediatric convulsions, has recently been implicated in the regulation of sepsis-associated thrombosis. In LPS-induced septic models, ZXP significantly reduced hepatic microthrombosis and corrected thrombocytopenia. Mechanistically, it inhibited platelet STING signaling, impaired STING-dependent SNARE complex assembly, and reduced P-selectin secretion, thereby suppressing neutrophil extracellular trap (NETs) formation. These effects were abolished by STING agonism, confirming STING as a critical molecular target [172].

The Compound Epimedium Formula (FEF) has been clinically applied in liver disease and demonstrated protective effects in acute liver injury (ALI) mouse models. FEF reduced serum ALT and AST levels, alleviated hepatic injury, oxidative stress, inflammation, and hepatocyte apoptosis. Mechanistically, FEF exhibits dual regulatory effects: activating the PI3K/AKT/Bcl-2 anti-apoptotic axis while concurrently suppressing the cGAS–STING–IRF3 inflammatory cascade, thereby reducing the production of TNF-α, IL-1β, and IL-6 [173]. In TCM theory, autoimmune and inflammatory diseases are often interpreted in relation to pathogenic heat, dampness, toxin accumulation, wind-damp obstruction, and deficiency-excess imbalance. Therefore, formulas or compounds that inhibit aberrant cGAS–STING activation may be understood as clearing heat and toxins, dispelling wind and dampness, unblocking collaterals, and restoring yin–yang balance.

Collectively, these studies highlight STING signaling as a key molecular hub in autoimmune and inflammatory disorders, representing an important target for TCM interventions. Herbal formulas exert coordinated multi-component, multi-target, and multi-pathway effects to modulate complex pathological processes via the cGAS–STING pathway.

Liver fibrosis is a common pathological process that underpins the progression of various chronic liver diseases toward cirrhosis. Central to this process is the activation of hepatic macrophages. ErTao Decoction (ETD), a modified formulation combining ErChen Decoction and TaoHongSiWu Decoction, has been shown to dose-dependently reduce liver damage and collagen deposition while improving liver function in a CCl₄-induced mouse model of liver fibrosis. Mechanistic studies indicate that ETD attenuates M1 macrophage polarization by inhibiting the activation of the STING signaling pathway in macrophages, while also suppressing NLRP3 inflammasome activation. This is evidenced by decreased levels of NLRP3, Caspase-1, IL-1β, and GSDMD. Further experiments in STING-deficient mice confirmed that STING deficiency mimics the hepatoprotective effects of ETD, underscoring the importance of the STING pathway in liver fibrosis progression [117].

Similarly, non-alcoholic fatty liver disease (NAFLD), another chronic liver disorder closely associated with macrophage-mediated inflammation, has also been linked to STING activation. Lingguizhugan Decoction (LGZG) significantly reduced hepatic lipid accumulation, liver injury, oxidative stress, and insulin resistance in high-fat diet-induced NAFLD models. These beneficial effects were associated with inhibition of the STING–TBK1–NF-κB signaling pathway in hepatic macrophages, resulting in reduced production of inflammatory cytokines such as IFN-β and TNF-α. Further investigations identified cinnamaldehyde and glycyrrhizic acid as major active constituents responsible for these effects. Both compounds directly suppressed STING pathway activation and decreased the expression of phosphorylated TBK1 and NF-κB, further supporting their role in regulating STING signaling [35].

Taken together, these findings suggest that activation of the STING pathway in hepatic macrophages represents a common downstream mechanism driving chronic liver disease progression. Regardless of whether the initiating insult is chemical injury, such as CCl₄ exposure, or metabolic stress induced by a high-fat diet, aberrant STING activation appears to be a key contributor to hepatic inflammation and fibrosis.

Cardiovascular diseases (CVDs) remain the leading cause of mortality worldwide and encompass a broad range of pathological conditions, including myocardial ischemia–reperfusion injury, chronic heart failure, diabetic cardiomyopathy, and atherosclerosis. Despite their diverse etiologies, mitochondrial dysfunction and chronic inflammation are common pathological hallmarks. Increasing evidence has identified the cGAS–STING pathway as a central signaling hub linking cellular stress to inflammatory responses during cardiovascular disease progression. This pathological framework is compatible with the TCM understanding of qi deficiency, blood stasis, and collateral obstruction in cardiovascular disorders.

Chronic heart failure (CHF), the terminal stage of many cardiovascular diseases, is closely associated with mitochondrial dysfunction and the activation of the cGAS/STING pathway, driven by mtDNA leakage. Xin-Ji-Er-Kang (XJEK) has been shown to significantly alleviate myocardial hypertrophy, collagen deposition, and ventricular remodeling in ischemia–reperfusion-induced CHF mouse models, thereby improving cardiac function. XJEK’s protective effects are mediated through the promotion of NR3C1 nuclear translocation, which enhances its binding to the mitofusin 2 (MFN2) promoter, leading to upregulation of MFN2 expression. This restoration of mitochondrial homeostasis reduces mtDNA leakage, thereby inhibiting the activation of the cGAS-STING pathway and downstream inflammatory responses [133].

Diabetic cardiomyopathy (DCM) is a major cardiovascular complication in diabetes, with myocytic pyroptosis playing a key role in its pathogenesis. Tao Hong Si Wu decoction (TSD) has been shown to significantly improve cardiac structure and function in a db/db diabetic cardiomyopathy mouse model, alleviating myocardial fibrosis and mitochondrial damage while reducing oxidative stress and inflammation. In a palmitic acid-induced HL-1 cardiomyocyte injury model, TSD similarly enhanced cell viability, reduced MDA levels, and restored SOD activity and mitochondrial membrane potential. Mechanistically, TSD inhibits the cGAS-STING signaling pathway and downstream NLRP3 inflammasome activation, thereby reducing cardiomyocyte pyroptosis. This is evidenced by decreased expression of Cleaved-caspase-1, GSDMD-N, IL-1β, IL-6, and TNF-α [174].

Atherosclerosis (AS) is a chronic inflammatory vascular disease where macrophage heterogeneity and functional plasticity play central roles in its pathogenesis. Kuanxiong Aerosol (KX), a traditional Chinese medicine used clinically for treating angina pectoris, inhibits AS progression in a high-fat diet-induced ApoE-/- mouse model and promotes regression of established plaques. Molecular docking studies indicate that KX’s active components can directly bind to cGAS, STING, TBK1, and p65. KX exerts its therapeutic effects on AS by inhibiting the cGAS-STING-P65 signaling pathway, thereby reducing the differentiation and apoptosis of CX3CR1+ macrophages [175].

Similarly, the Trichosanthes-Allium compound dose-dependently reduces aortic plaque area in an ApoE-/- high-fat diet-induced AS model, while alleviating endothelial cell damage and inflammatory responses. In an ox-LDL-induced injury model using human umbilical vascular endothelial cells (HUVECs), this compound enhances cell viability, reduces MDA and ROS levels, and restores SOD activity. Studies show that it inhibits the expression of the scavenger receptor LOX-1, blocking cGAS-STING pathway activation mediated by LOX-1. This downregulates NCOA4 expression, inhibits NCOA4-mediated ferritin phagocytosis, reduces free iron release and lipid peroxidation, and ultimately suppresses ferroptosis in endothelial cells [176].

Collectively, these studies indicate that mitochondrial dysfunction serves as a major upstream trigger of STING activation in cardiovascular diseases. By preserving mitochondrial homeostasis and suppressing aberrant STING signaling, TCM herbal formulas exert significant cardioprotective effects across a variety of cardiovascular disorders.

Neurodegenerative diseases and neurological injuries comprise a complex group of disorders characterized by progressive neuronal loss and persistent neuroinflammation. Their pathogenesis involves multiple factors, including mitochondrial dysfunction, abnormal protein aggregation, oxidative stress, and dysregulated innate immune responses. Both mtDNA released from damaged mitochondria and nuclear DNA fragments leaked under cellular stress can act as danger-associated molecular patterns (DAMPs), thereby activating the cGAS–STING pathway. Subsequent activation of microglia-mediated inflammatory responses exacerbates neuronal injury and accelerates disease progression. Consequently, targeting the cGAS–STING pathway has emerged as a promising therapeutic strategy for neurological disorders.

Dingzhen pills (DZP) have been shown to significantly improve motor deficits, protect dopaminergic neurons in the substantia nigra, and reduce α-synuclein aggregation in an MPTP-induced mouse model of Parkinson’s disease. Mechanistically, DZP inhibits the cGAS-STING pathway. Proteomic and network pharmacology analyses indicate that multiple active components in DZP act on several targets that converge on the cGAS-STING pathway, thereby suppressing downstream neuroinflammation and ferroptosis, ultimately producing neuroprotective effects [177]. Because this conclusion is partly based on proteomic and network pharmacology analyses, it should be interpreted as pathway-level evidence. Further target-validation experiments are needed to determine which DZP constituents directly regulate cGAS, STING, or downstream signaling proteins.

Buyang Huanwu Decoction (BYHWD) markedly improves learning and memory in a rat model of vascular cognitive impairment induced by bilateral common carotid artery ligation. BYHWD exerts its effects by modulating the p53/cGAS/STING signaling pathway: it downregulates p53, upregulates the DNA exonuclease TREX1 to promote cytoplasmic DNA degradation, thereby inhibiting cGAS-STING activation and its downstream IRF3/NF-κB signaling. This leads to the alleviation of microglial-mediated neuroinflammation and preservation of cognitive function [178].

Depression, one of the most prevalent mental disorders worldwide, poses a significant threat to public mental health. Zhi-Zi-Hou-Po decoction (ZZHP) has been shown to exert antidepressant effects in a mouse model of depression induced by chronic, unpredictable, mild stress, improving sucrose preference and reducing the duration of behavioral despair. The core mechanism involves upregulation of mitochondrial transcription factor A (TFAM) in hippocampal neurons, which enhances the TFAM-LC3 interaction to promote mitochondrial autophagy. This process effectively clears damaged mitochondria and cytoplasmic mtDNA, thereby inhibiting cGAS-STING-mediated neuroinflammation and reducing neuronal apoptosis [179].

Similarly, Jiao-tai-wan (JTW) and its active component jatrorrhizine alleviate hippocampal neuronal damage and neuroinflammation and improve depressive-like behaviors in a chronic restraint stress-induced depression mouse model. Mechanistically, they inhibit the STING signaling pathway in microglia. In vitro studies further demonstrate that jatrorrhizine suppresses STING and downstream TBK1/IRF3/NF-κB phosphorylation in LPS-induced BV2 microglia, decreases expression of M1 polarization markers, and enhances M2 marker expression, thereby exerting anti-inflammatory and neuroprotective effects [180].

Collectively, these studies demonstrate that modulation of the cGAS–STING pathway represents a common mechanism through which TCM formulas alleviate neurodegenerative diseases and neurological injuries. Although the herbal formulations and disease models differ, their underlying therapeutic logic is highly consistent: suppression of aberrant cGAS–STING activation in innate immune cells of the central nervous system. Furthermore, the integration of network pharmacology, molecular docking, and experimental validation provides increasing mechanistic support for STING as a promising therapeutic target in neurological disorders.

Aberrant activation of the STING pathway contributes not only to classical inflammatory disorders but also to the development of metabolic diseases and their associated complications. Although type 2 diabetes mellitus (T2DM), diabetic wound-healing impairment, and age-related osteoporosis exhibit distinct clinical manifestations, increasing evidence suggests that activation of the cGAS–STING pathway represents a shared pathological mechanism linking metabolic stress to tissue injury.

In a mouse model of obesity-associated type 2 diabetes induced by a high-fat diet combined with STZ, Jiangtang Tiaozhi Formula (JTTZF) significantly reduced body weight, blood glucose levels, and insulin resistance. It also improved dyslipidemia, alleviated hepatic steatosis, and mitigated inflammatory responses by inhibiting the hepatic cGAS-STING pathway [181].

Delayed wound healing is a major complication of diabetes and is closely associated with endothelial dysfunction. In diabetic wound models, San Huang Decoction (SHD) alleviates endothelial cell senescence and apoptosis by inhibiting the STING/TBK1-IRF3 signaling pathway in endothelial cells. This inhibition restores endothelial cell migration and tubule formation capabilities, thereby accelerating wound healing. Network pharmacology and molecular docking studies further confirm that SHD’s active components exhibit strong binding affinity for key proteins in the STING pathway [182].

Age-related osteoporosis is characterized by progressive bone loss and impaired bone regeneration. Wen-Shen-Tong-Luo-Zhi-Tong Decoction (WSTLZTD) improves age-related osteoporosis in a natural aging mouse model by regulating LONP1-mediated macrophage senescence. This formula upregulates the expression of LONP1, a mitochondrial protease in macrophages, inhibiting macrophage senescence and mitigating the negative impact on bone marrow mesenchymal stem cells (BMSCs). It also suppresses cGAS/STING pathway activation in BMSCs, improves mitochondrial function, and promotes osteogenic differentiation of BMSCs, leading to increased bone density and improved bone microstructure [36].

Collectively, these findings suggest that metabolic stress, STING activation, and tissue injury may constitute a common pathological cascade underlying a variety of metabolic disorders. By targeting different stages of this cascade, TCM formulas may exert broad therapeutic effects across multiple metabolic disease settings.

Taken together, TCM compound preparations regulate the STING pathway through diverse mechanisms across a wide range of disease contexts. Although their intervention modes and pathological targets vary considerably, a clear pattern emerges. Activation of STING signaling is generally associated with enhanced antitumor and antimicrobial immunity, whereas suppression of aberrant STING activation predominates in inflammatory, fibrotic, autoimmune, metabolic, and degenerative disorders. Through coordinated actions on multiple components, targets, and signaling pathways, TCM compound preparations establish complex immunoregulatory networks that contribute to the restoration of immune homeostasis.

Taken together, the monarch–minister–assistant–guide principle provides a useful conceptual framework for interpreting the hierarchical and complementary actions of TCM compound prescriptions. At the molecular level, monarch herbs may provide the principal constituents acting on dominant pathological events or central signaling nodes; minister herbs may reinforce these effects through complementary regulation of upstream stress signals, downstream immune responses, or parallel inflammatory pathways; assistant herbs may enhance efficacy or restrain excessive pathway activation and toxicity; and guide herbs may influence formula compatibility, pharmacokinetics, and tissue exposure. However, this correspondence remains provisional for most formulas reviewed in this article.

Most available studies demonstrate changes in cGAS, STING, TBK1, IRF3, or related inflammatory responses only after administration of the complete prescription. Such findings do not establish the respective contributions of the monarch, minister, assistant, and guide herbs, nor do they identify the constituents responsible for direct or indirect pathway regulation. A more rigorous analysis should compare the complete formula with individual herbs, herb-deleted formulas, active fractions, and reconstructed combinations. Constituent-removal and add-back experiments, together with pharmacokinetic analysis and cGAS- or STING-dependent rescue experiments, are required to determine whether individual components regulate upstream danger signals, the core cGAS–STING complex, downstream inflammatory networks, tissue distribution, or treatment-related toxicity. Therefore, traditional compatibility theory should guide the formulation of mechanistic hypotheses, but component-level molecular functions should ultimately be defined through experimental evidence.

The regulation of cGAS–STING signaling by compound prescriptions may involve cooperation among multiple constituents. Some components may act on different nodes of the same pathway, including mitochondrial damage, mtDNA release, STING activation, and downstream TBK1–IRF3 or NF-κB signaling. Others may regulate related processes such as oxidative stress, autophagy, inflammasome activation, and immune-cell function, thereby modifying the overall pathway response.

Multicomponent interactions may also occur at the pharmacokinetic and safety levels. Certain constituents may improve the solubility, absorption, stability, or tissue distribution of pathway-active compounds, whereas others may limit excessive inflammation or reduce treatment-related toxicity. Such restrictive effects may help maintain an appropriate therapeutic window rather than simply weaken the principal effect of the formula.

Conversely, functional antagonism may occur when different constituents exert opposing effects on STING signaling or compete for metabolic enzymes and transporters. However, most current studies evaluate complete formulas and cannot distinguish true synergy or antagonism from additive or dominant-component effects. Formula-decomposition, component-removal and add-back experiments, pharmacokinetic analyses, and pathway-specific validation are therefore required to clarify these interactions.

#### Traditional Chinese Medicine extracts

Unlike purified compounds, TCM extracts contain multiple chemical constituents and should not be treated as single molecular entities. Their effects may be driven by one dominant constituent, several co-active constituents, or indirect changes in cellular stress and immune function. Therefore, unless component-depletion or combination experiments have been performed, the available evidence should be interpreted as an extract-level effect rather than experimentally confirmed multicomponent synergy. Epimedium brevicornu Maxim. extract (EPE) and Sophora tonkinensis extract (STE) illustrate two distinct patterns of extract-mediated regulation of cGAS–STING signaling.EPE primarily acts downstream of the STING pathway, activating STING signaling to enhance NK cell effector functions, as evidenced by the increased release of IFN-γ, granzyme B, and perforin [183]. In contrast, STE acts upstream of the pathway, initiating STING activation by promoting cGAS-mediated synthesis of the second messenger cGAMP, which inhibits viral replication in a mouse model of HBV [66]. This complementary mechanism suggests that different TCM extracts may achieve multi-level, multidimensional regulation of immune responses by modulating various nodes of the STING pathway, thereby the reflecting systemic characteristic of multi-target intervention in TCM. Mechanistically, EPE mainly enhances downstream NK-cell effector responses, but the increased expression of IFN-γ, granzyme B, and perforin does not establish which constituent activates STING or whether the effect occurs directly in NK cells. STE-induced cGAMP production provides stronger evidence for upstream cGAS involvement, although the responsible constituents and their direct interaction with cGAS remain unknown. Fractionation-guided activity testing, chemical characterization of active fractions, constituent-depletion and reconstitution experiments, and cGAS/STING loss-of-function models are needed to distinguish dominant active constituents from genuine multicomponent cooperation.

Research on Artemisia-derived nanovesicles (ADNVs) represents a breakthrough in the study of TCM extracts. These naturally derived nanoscale vesicular structures can deliver plant mitochondrial DNA to tumor-associated macrophages, where it is recognized as an “exogenous danger signal,” activating the cGAS–STING pathway. This reprograms pro-tumor alternatively activated M2 macrophages (M2) macrophages into the anti-tumor classically activated M1 macrophages (M1) phenotype, effectively inhibiting tumor growth [184]. This finding suggests that naturally occurring nanovesicles may be widely distributed among medicinal plants and that plant-derived nucleic acids can directly participate in the regulation of mammalian innate immunity. Since Artemisia annua is traditionally associated with clearing heat and eliminating pathogenic factors, ADNV-mediated activation of innate immune sensing may represent a modern nanoscale interpretation of this classical TCM function. Unlike conventional herbal extracts, the activity of ADNVs depends not only on their molecular cargo but also on vesicle uptake, intracellular trafficking, and cytosolic cargo release. Current evidence supports plant-derived mtDNA as an immunostimulatory cargo that activates cGAS after delivery into tumor-associated macrophages. However, it remains unclear whether intact vesicles are required for efficient cytosolic delivery and whether vesicular lipids, proteins, or small molecules contribute to macrophage targeting, endosomal escape, or downstream signal amplification. Further studies using disrupted or cargo-depleted vesicles, purified-cargo reconstitution, and cGAS- or STING-deficient macrophages are needed to clarify these mechanisms.

Collectively, these studies demonstrate that TCM extracts possess diverse molecular mechanisms for regulating the STING pathway. Their regulatory patterns have evolved from investigations of single compounds toward analyses of bioactive constituent groups, and from single-target regulation toward coordinated multi-node modulation. Future studies should focus on elucidating the receptor-level mechanisms through which extract-derived constituents interact with immune cells, thereby providing a stronger theoretical foundation for the development of novel immunomodulatory agents.

Myocardial ischemia–reperfusion injury (MIRI) is a severe complication that frequently occurs following revascularization therapy for acute myocardial infarction. Mitochondrial dysfunction and aberrant activation of the STING pathway are recognized as major contributors to its pathogenesis.

Tanshinone IIA (Ta-IIA), a major bioactive constituent of *Salvia miltiorrhiza* (Danshen), and Astragaloside IV (As-IV), a principal active compound derived from *Astragalus membranaceus* (Huangqi), have both demonstrated cardioprotective properties against MIRI. In murine models of myocardial ischemia–reperfusion injury, combined administration of Ta-IIA and As-IV exhibited superior therapeutic efficacy compared with either compound alone, resulting in reduced infarct size, decreased serum cardiac enzyme levels, improved cardiac function, and attenuated histopathological damage.

Similarly, in cardiomyocyte hypoxia–reoxygenation models, the combination exhibited potent anti-apoptotic, antioxidant, and anti-inflammatory effects. Mechanistically, both Ta-IIA and As-IV bind directly to the STING protein, inhibiting its phosphorylation as well as the subsequent phosphorylation of TBK1 and IRF3, thereby preventing the activation of the STING pathway. Notably, the protective effects of this combination were reversed by STING agonists, underscoring STING as the key molecular target mediating their synergistic cardioprotective action [185].

Collectively, current studies support extract- or vesicle-level regulation of cGAS–STING signaling, but component-level mechanisms remain incompletely resolved. Future work should distinguish direct target engagement from indirect pathway activation and determine whether the observed effects are mediated by dominant constituents, multicomponent cooperation, or vesicle-dependent intracellular delivery.

#### Traditional Chinese Medicine nanomedicines

The integration of nanotechnology with Traditional Chinese Medicine has substantially improved the bioavailability, stability, and tissue-targeting capacity of TCM-derived therapeutics. Current studies frequently combine bioactive TCM constituents with functional nanomaterials or metal ions to construct multifunctional nanosystems capable of synergistically activating antitumor immune responses through modulation of the cGAS–STING pathway.

As a direct activator of the STING pathway, Mn^2^⁺ demonstrates excellent synergistic effects when combined with TCM components. For example, a study developed nanoparticles loaded with quercetin and Mn^2^⁺ (AHA@MnP/QCT NPs). Through Mn^2^⁺-mediated activation of the cGAS–STING pathway, these nanoparticles promote dendritic cell maturation and T-cell infiltration, while quercetin induces ferroptosis, resulting in a combination of diagnosis and treatment and demonstrating dual synergy with lung cancer immunotherapy [186].

Similarly, a study embedded Dendrobium polysaccharide hydrogels (MnP@DOP-Gel) into manganese microspheres and implanted them into melanoma-bearing mice post-surgery. The results showed that they activated the STING pathway and initiated an antitumor immune cascade [187].

In another example, a pH-responsive nanosystem co-loaded with a DNAzyme and glycyrrhizic acid releases Mn^2^⁺ in the tumor microenvironment to activate STING, while glycyrrhizic acid induces immunogenic cell death, synergistically activating antitumor immunity [188].

In addition to Mn^2^⁺, other metal ions have also been used to construct TCM-based nanomedicines to regulate the STING pathway. A bio-inspired nanoparticle formulation loaded with Pizihuang (PZH/Zn@CaNA) activates STING by releasing DAMPs through the induction of pyroptosis following microwave ablation of liver cancer, thereby reshaping the immunosuppressive microenvironment [189].

The nanoparticle cLipG/CuET, based on a glycyrrhizic acid-copper complex, targets mitochondria to induce mitochondrial damage and the release of mtDNA, thereby activating STING in macrophages, promoting M1 polarization, and enhancing the efficacy of immunotherapy for cholangiocarcinoma [190].

Furthermore, a biomimetic nanoplatform (PZ@M-T) loaded with Paridis saponin II, under the synergy of Zn^2^⁺, reshapes the immune microenvironment of liver cancer by inducing ferroptosis and mtDNA release, thereby heterologously activating STING [191].

Collectively, these studies illustrate how modern nanotechnology can be integrated with TCM-derived bioactive compounds to amplify STING-mediated antitumor immunity. Such approaches substantially enhance therapeutic efficacy and highlight the considerable translational potential of TCM-based nanomedicines in cancer treatment.

Implant-related infections (IRIs) are severe complications following orthopedic surgery. Bacterial biofilms shield bacterial-associated antigens (BAAs) and create an immunosuppressive microenvironment resembling that of “cold tumors,” making infection eradication particularly challenging.

To address this problem, Xu et al. developed an iron-based covalent organic framework nano-adjuvant (CFCP) centered on curcumin, a bioactive constituent derived from TCM. Under ultrasound irradiation, platinum single-atom catalytic sites enhanced the sonodynamic therapeutic effect of curcumin, generating abundant reactive oxygen species (ROS) that disrupted biofilm structures and effectively eliminated bacteria. Simultaneously, iron ions released from CFCP interfered with bacterial energy metabolism, including glycolysis, thereby further accelerating bacterial death.

As the biofilm is disrupted, immunogenic bacterial-derived double-stranded DNA (dsDNA) is released and recognized by cGAS in dendritic cells (DCs), thereby activating the STING signaling pathway. This promotes DC maturation and IFN-β secretion. IFN-β not only enhances innate immune activity of neutrophils (e.g., promoting neutrophil extracellular trap formation, degranulation, and phagocytosis), but also induces adaptive immune responses through DC-mediated activation of B and T cells, including antibody production and memory B-cell formation. Together, these effects provide long-term anti-infective protection for implants and effectively prevent infection recurrence and reinfection [192].

This work established a multi-step immune activation cascade driven by bacterial dsDNA-mediated STING activation and represents an excellent example of the integration of modern nanotechnology with TCM-derived active compounds.

Nanotechnology-based delivery systems have also demonstrated therapeutic potential in cisplatin-induced acute kidney injury (CIAKI). A dual-targeting nanosystem (Fu-4-PBA/Po NPs) was developed by co-encapsulating polydatin within a nanocarrier composed of fucoidan and 4-phenylbutyric acid (4-PBA). Fucoidan targets P-selectin, which is highly expressed at inflammatory sites, whereas 4-PBA preferentially accumulates in the endoplasmic reticulum.

This nanosystem significantly improved the bioavailability and renal accumulation of polydatin. Both in vitro and in vivo studies demonstrated that Fu-4-PBA/Po NPs alleviated CIAKI, oxidative stress, and apoptosis by suppressing endoplasmic reticulum stress, improving mitochondrial function, and reducing DNA damage. Consequently, activation of the cGAS–STING pathway was effectively inhibited [93].

These findings illustrate how the therapeutic principles of TCM can be combined with advanced nanodelivery technologies to improve pharmacokinetics and therapeutic efficacy.

Rheumatoid arthritis is an autoimmune disease characterized by chronic inflammation of the synovial joints, with progression closely linked to the massive infiltration of M1-type macrophages into the synovial membrane and their secretion of inflammatory factors. Targeting this pathological feature, Xu et al. developed folate-modified dextran-lauric acid amphiphilic polymer micelles for the targeted delivery of triptolide (FDL@TP).

These nanomicelles exhibit excellent stability and biocompatibility, enabling active targeting and cellular uptake at the joint site. In an adjuvant-induced arthritis mouse model, FDL@TP significantly reduced joint swelling and bone destruction, showing superior efficacy compared to an equal dose of free TP, while also reducing its systemic toxicity. Studies have shown that this compound exerts anti-inflammatory and joint-protective effects by downregulating the expression of cGAS and STING proteins in M1 macrophages, thus inhibiting the secretion of downstream inflammatory factors such as TNF-α, IL-1β, and IL-6 [193].

Similarly, Xiao et al. developed pectin-ferulic acid nanoparticles (PC-FA NPs) to enhance the solubility and colon-targeted delivery of ferulic acid. In a DSS-induced colitis mouse model, PC-FA NPs significantly alleviated weight loss, elevated disease activity index (DAI) scores, and colonic shortening, while also improving colonic histopathological damage. Their mechanism of action involves scavenging ROS, reducing DSS-induced DNA damage in colonic epithelial cells, inhibiting the activation of the cGAS-STING pathway, and decreasing the production of downstream inflammatory factors such as TNF-α and IL-1β. Additionally, the study found that PC-FA NPs are primarily distributed in the gut and exhibit good colon-targeting, further demonstrating their significant potential in the treatment of ulcerative colitis [194].

Delayed wound healing is a common and debilitating complication of diabetes mellitus, often characterized by persistent inflammation, impaired angiogenesis, and dysregulated macrophage polarization. Recent studies have demonstrated that nanotechnology-based TCM formulations can effectively modulate the local immune microenvironment through regulation of the STING pathway.

In a diabetic mouse wound model, naringin-loaded SBMA/GelMA hydrogels significantly accelerated wound healing by activating PPARα and inhibiting the STING pathway. These hydrogels promoted M2 macrophage polarization, increased CD31-positive blood vessels and local blood perfusion, while simultaneously suppressing the release of pro-inflammatory cytokines factors IL-1β and TNF-α. This dual mechanism of regulating the immune microenvironment and promoting angiogenesis contributes to the enhanced healing process [195].

Collectively, these studies demonstrate that TCM nanomedicines regulate the STING pathway through diverse mechanisms and exhibit broad therapeutic potential across multiple disease settings. Although the design strategies and pathological contexts vary considerably, modulation of STING signaling remains a common mechanistic feature underlying their biological activities.

Compared with conventional herbal preparations, nanoformulations integrate TCM-derived bioactive compounds with functional materials such as metal ions, polymers, and biomimetic carriers, thereby enhancing bioavailability, tissue targeting, and therapeutic efficacy. In oncology, many nanoformulations exploit STING activation to amplify antitumor immunity; in inflammatory and degenerative disorders, they predominantly suppress aberrant STING signaling to alleviate pathological inflammation and tissue injury.

Despite these promising advances, most studies remain limited to in vitro and preclinical animal models, and robust clinical evidence is still lacking. Future investigations should prioritize the clinical translation of nanoformulations based on well-characterized and clinically validated TCM compounds or formulas. Furthermore, integration of artificial intelligence-assisted drug delivery systems may enable real-time monitoring, intelligent responsiveness, and precision targeting, thereby further improving therapeutic outcomes. Systematic evaluation of combination strategies involving immunotherapy, chemotherapy, or other treatment modalities will also be essential for identifying clinically translatable candidates and advancing the development of integrative medicine based on STING-targeted interventions（Table 2).

## Summary and outlook

This article systematically reviews recent advances in how TCM modulates the cGAS-STING signaling pathway to intervene in tumors, infections, inflammation, autoimmune diseases, organ damage, and metabolic disorders. Current evidence indicates that TCM regulates this pathway at multiple levels, including upstream danger signal generation, activation of the core signaling complex, and downstream inflammatory and interferon-mediated responses.

Despite the rapidly growing body of evidence showing that TCM can regulate the cGAS-STING pathway across diverse disease settings, a critical synthesis of the available literature reveals three major conclusions.

TCM-mediated cGAS-STING modulation is inherently bidirectional and context-dependent, aligning with the holistic principles of balancing healthy *qi* and eliminating pathogens—operating not as a binary switch but as a dynamic rebalancing of immune homeostasis via multi-component, multi-node coordination. The intervention modalities span herbal formulas, purified compounds/extracts, and nanoformulations; among these, purified agents offer the most mechanistically tractable path for translational development, whereas formulas require material-base deconvolution and nanoformulations await maturation beyond proof-of-concept. Currently, the most robust evidence resides in oncology (diverse activation mechanisms and synergy with checkpoint inhibitors) and autoimmune/inflammatory diseases (consistent validation across colitis, arthritis, Sjögren’s syndrome, and hepatitis). In contrast, cardiovascular, metabolic, and neurodegenerative conditions remain nascent, with evidence largely restricted to pathway-level correlates and lacking rigorous direct target validation—highlighting priority areas for future mechanistic and therapeutically oriented inquiry. In general, TCM regulates cGAS-STING signaling through multi-component and multi-node networks to achieve context-dependent bidirectional immunomodulation. Purified compounds and well-defined extracts currently possess the strongest mechanistic support, whereas cancer and autoimmune/inflammatory diseases represent the best-supported therapeutic applications. This framework may help shift the field from descriptive literature accumulation toward mechanism-driven and clinically translatable research.

Several TCM-derived compounds, such as quercetin and curcumin, have been reported to produce opposite effects on cGAS-STING signaling in different experimental models. Rather than simply classifying these compounds as either “activators” or “inhibitors,” these findings should be interpreted in light of the specific disease context and experimental conditions. For example, in tumor or viral infection models, enhancement of cGAS-STING signaling may help restore antitumor or antiviral immune responses. In contrast, in autoimmune disease, tissue injury, fibrosis, or other settings with excessive inflammation, suppression of overactivated cGAS-STING signaling may be protective.

Such divergent observations may also be related to differences in cell type, disease stage, basal inflammatory status, dose, treatment duration, and the readouts used to define pathway activation. Moreover, many studies infer cGAS-STING regulation mainly from changes in downstream markers such as STING, TBK1, IRF3, IFN-β, or inflammatory cytokines. These changes do not necessarily indicate direct regulation of cGAS or STING, since they may also result from upstream alterations in mitochondrial function, cytosolic DNA accumulation, oxidative stress, autophagy, or broader inflammatory signaling.

Therefore, compounds such as quercetin and curcumin are better viewed as context-dependent modulators of cGAS-STING-related responses, rather than as fixed activators or inhibitors. This perspective is also consistent with the broader concept of TCM in restoring pathological imbalance. However, the mechanistic basis of such bidirectional regulation remains insufficiently defined. Future studies should use more rigorous designs, including dose- and time-dependent analyses, pathway-specific loss- or gain-of-function experiments, and rescue approaches, to determine whether these compounds act directly on the cGAS-STING axis or indirectly through upstream stress and inflammatory networks.

Notably, mitochondrial damage, mtDNA release, and STING activation are widely observed across respiratory, digestive, urinary, cardiovascular, and neurological diseases, suggesting that this axis may represent a shared pathological basis for multiple chronic disorders and a key therapeutic entry point for TCM. From the perspective of TCM theory, this context-dependent regulation reflects the principles of strengthening healthy qi and eliminating pathogenic factors, balancing yin and yang, and treating deficiency and excess differentially. Activation of cGAS-STING signaling may be beneficial when host defense or immune surveillance is insufficient, whereas inhibition of aberrant activation may be necessary when inflammatory injury or immune imbalance predominates. In this context, cGAS-STING-mediated immune regulation involves broad pathway crosstalk. The cGAS-STING pathway is closely connected with NF-κB, NLRP3 inflammasome, MAPK, oxidative stress, autophagy, ferroptosis, pyroptosis, apoptosis, and senescence-related signaling. TCM regulation of cGAS-STING should therefore be viewed as remodeling of an immune-inflammatory network. It should not be viewed as regulation of one pathway alone. Future studies should clarify how TCM compounds and formulas coordinate these related pathways in different disease settings.

Beyond individual compounds, the use of herbal formulas and novel delivery systems has further expanded research on TCM modulation of the cGAS-STING pathway. Different formulas can achieve differential regulation by targeting STING, TBK1, IRF3, and their interaction interfaces. Some formulations demonstrate synergistic effects, even when individual herbs show limited activity, highlighting the advantages of multi-component coordination in TCM. Meanwhile, emerging delivery technologies—such as plant-derived nanovesicles and metal-ion co-loaded nanosystems—enhance the stability, bioavailability, and targeted accumulation of active compounds, providing a technical foundation for optimizing TCM-based interventions targeting the cGAS-STING pathway. Overall, TCM intervention in this pathway is gradually evolving from empirical approaches toward a modern framework integrating targets, mechanisms, and delivery systems.

However, several limitations still hinder progress in this field. Most existing studies on TCM-mediated regulation of the cGAS-STING pathway remain at the preclinical stage, including in vitro experiments, animal models, network pharmacology, molecular docking, and nanomedicine-based studies. To our knowledge, there are currently no well-designed clinical trials directly demonstrating that TCM compounds, herbal formulas, or TCM-derived nanomedicines regulate the cGAS-STING pathway in patients. Therefore, the clinical efficacy, safety, optimal indications, and suitable patient populations for TCM interventions targeting this pathway remain to be established. Although molecular docking has suggested potential binding modes for several active constituents, only a limited number of studies have further used biophysical binding assays, site-directed mutagenesis, target-engagement experiments, or structural approaches to support direct interactions with cGAS or STING. Most studies therefore remain at the level of pathway-associated molecular changes rather than experimentally confirmed target engagement, most studies remain at the level of correlational observations of pathway-related molecular changes. It remains challenging to clearly distinguish direct targeting from indirect regulation, limiting mechanistic clarity. Another important limitation is the uneven quality of evidence across the included studies. Some studies provide relatively strong mechanistic support through direct binding assays, genetic knockdown or knockout, pathway rescue experiments, or pharmacological agonist and inhibitor validation. However, many other studies only report changes in pathway-related proteins or inflammatory markers. Some studies rely mainly on network pharmacology, molecular docking, or multi-omics prediction. These methods are useful for generating hypotheses, but they cannot by themselves confirm that TCM directly regulates the cGAS-STING pathway. Future studies should clearly distinguish direct target engagement, indirect pathway modulation, and predicted pathway involvement. Another major translational obstacle is the insufficient standardization and quality control of TCM interventions. The chemical composition and biological activity of herbal medicines may vary according to botanical species, geographical origin, cultivation and harvesting conditions, processing procedures, extraction solvents, herb ratios, and manufacturing parameters. Such variability is particularly important for cGAS-STING modulation because changes in the abundance or proportion of active constituents may alter not only the magnitude but potentially also the direction of pathway regulation. Quantification of one or two marker compounds alone may therefore be insufficient to ensure reproducible pharmacological activity. Future studies should integrate botanical authentication, standardized processing and extraction procedures, chromatographic or mass-spectrometric fingerprinting, multi-component quantitative analysis, stability testing, and batch-to-batch consistency assessment. In addition to chemical quality control, pathway-related potency assays based on cGAMP production, STING phosphorylation or trafficking, TBK1/IRF3 activation, and IFN-I responses should be developed to confirm the biological equivalence of different batches. For TCM-derived nanomedicines and plant-derived vesicles, particle size distribution, surface charge, encapsulation efficiency, release kinetics, storage stability, sterility, endotoxin levels, and reproducibility of tissue targeting should also be systematically characterized.Dose selection and safety evaluation represent another insufficiently addressed barrier. Many available studies report effective concentrations in cultured cells or administered doses in animals but provide limited information on systemic exposure, bioavailability, tissue distribution, pharmacokinetics, maximum tolerated dose, repeated-dose toxicity, or human-equivalent dose. These issues are especially relevant to the cGAS-STING pathway because its therapeutic window is likely to be context dependent. Insufficient pathway activation may fail to induce effective antiviral or antitumor immunity, whereas excessive or prolonged activation may cause systemic inflammation, autoimmune-like responses, and tissue injury. Conversely, excessive inhibition may impair host defense against infection and weaken tumor immune surveillance. Therefore, future studies should establish integrated dose–exposure–pathway response–efficacy–toxicity relationships rather than evaluating efficacy and pathway-related protein expression at a single dose. Pharmacokinetic and pharmacodynamic analyses should be combined with dose-ranging studies, repeated-dose toxicity testing, and assessments of hepatic, renal, hematological, reproductive, and immune toxicity. Potential herb–drug interactions, accumulation of active constituents in target and non-target tissues, and additional toxicity associated with nanocarriers or metal-ion-assisted delivery systems should also be evaluated before clinical translation.

An additional translational barrier is the lack of validated, pathway-specific biomarkers for cGAS-STING modulation in clinical settings. Current studies frequently infer pathway engagement from downstream cytokines or transcriptional changes, but these readouts are not specific to the cGAS-STING axis and may also reflect parallel inflammatory, metabolic, or cellular stress responses. The absence of validated and clinically accessible target-engagement biomarkers makes it difficult to stratify patients according to basal pathway activity, distinguish direct pathway modulation from nonspecific anti-inflammatory effects, and interpret therapeutic outcomes in mechanism-driven clinical trials.

Although compound prescriptions may regulate cGAS-STING signaling through pharmacodynamic, pharmacokinetic, and toxicity-attenuating interactions, most proposed synergistic or antagonistic relationships remain insufficiently validated. Future studies should combine formula-decomposition, constituent-removal and add-back experiments, pharmacokinetic analyses, quantitative interaction models, and pathway-specific validation to define the contribution of individual components. Taken together, the major translational barriers in this field can be summarized into five interconnected dimensions: product standardization and quality control, dose–exposure-toxicity relationships, pathway-specific biomarker development, mechanistic deconvolution of multicomponent interactions, and the limited relevance of current experimental models to human disease. These barriers should not be addressed independently, because batch composition influences systemic exposure, exposure determines the magnitude and direction of pathway modulation, biomarkers are required to define target engagement and therapeutic windows, and multicomponent interactions may simultaneously affect efficacy, pharmacokinetics, and toxicity. Variability in experimental models, evaluation criteria, and mechanistic interpretations further reduces comparability and reproducibility across studies. More importantly, the cGAS-STING pathway exhibits a pronounced dual role depending on disease context, yet there is still a lack of stratification criteria based on disease stage, inflammatory status, and tissue microenvironment to guide when the pathway should be activated or inhibited. Furthermore, structural and ligand recognition differences between human and mouse STING pose significant challenges, as findings from animal models may not be directly translatable to humans, representing a major barrier to clinical application.

Future research should be advanced along several key directions. First, direct target validation needs to be strengthened. For active components that modulate the cGAS-STING pathway, integrated approaches—including surface plasmon resonance, hydrogen–deuterium exchange mass spectrometry, cryo-electron microscopy, and site-directed mutagenesis—should be employed to define their binding sites, interaction modes, and structure–activity relationships with cGAS, STING, or IRF3. This will promote the transition from pathway-level observations to precise target-based mechanistic studies.

Second, the material basis and synergistic or antagonistic mechanisms of compound formulations should be systematically elucidated. High-resolution mass spectrometry, network pharmacology, single-cell transcriptomics, and spatial transcriptomics may be used to identify candidate constituents, responsive cell populations, and potentially regulated pathway nodes. However, these hypothesis-generating approaches should be combined with formula-decomposition, herb-subtraction, constituent-removal and add-back experiments, pairwise or multicomponent dose matrices, quantitative synergy analyses, pharmacokinetic interaction studies, and pathway-specific rescue experiments. This integrated strategy is necessary to distinguish true pharmacodynamic synergy from additive effects, pharmacokinetic enhancement, toxicity attenuation, or functional antagonism.

Third, greater attention should be paid to the subcellular dynamic regulation of the cGAS-STING pathway. This pathway involves finely tuned processes, including cGAS phase separation, STING trafficking from the endoplasmic reticulum to the Golgi via the ERGIC, and lysosome-dependent degradation. Emerging evidence—such as cordycepin promoting STING autophagic degradation and paeoniflorin inhibiting STING lysosomal degradation—highlights the relevance of this layer of regulation. Future studies should further investigate how small-molecule TCM compounds modulate these events with spatiotemporal precision, which may represent a critical breakthrough beyond conventional pathway analysis.

Fourth, priority should be given to candidates with a history of clinical use, well-characterized chemical composition, reproducible batch quality, acceptable pharmacokinetic and safety profiles, and replicated efficacy across multiple experimental models. For compound formulas, extracts, and nanomedicines, a clearly defined manufacturing process and release criteria should be established before early-phase clinical trials.

Fifth, a stepwise pathway for clinical translation should be established in future studies. (1) Strict target validation should be performed for candidate TCM compounds, herbal formulas, and TCM-derived nanomedicines by integrating biochemical binding assays, gene knockdown or knockout models, and rescue experiments. These approaches are essential for distinguishing direct regulation of the cGAS-STING pathway from indirect anti-inflammatory or cytoprotective effects. (2) Mechanistic findings should be further verified in human primary cells, organoids, and humanized animal models, because human and mouse STING differ in ligand recognition and drug responsiveness. (3) Translational biomarkers should be developed for patient selection, confirmation of pathway engagement, monitoring of therapeutic responses, and early detection of immune-related toxicity. Baseline predictive biomarkers may include tissue cGAS/STING expression, cytosolic dsDNA or mtDNA accumulation, basal STING activation, IFN-I-related transcriptional signatures, and the immune-cell composition of the affected tissue. Pharmacodynamic biomarkers should preferentially include target-pMroximal indicators, such as cGAP production, STING phosphorylation and intracellular trafficking, TBK1/IRF3 activation, and IRF3 nuclear translocation, together with downstream markers such as IFN-β, CXCL10, CCL5, and interferon-stimulated gene signatures. Safety monitoring should include systemic inflammatory cytokines, organ-function indices, autoimmune manifestations, and evidence of infection or excessive immunosuppression. Because inflammatory cytokines such as IL-6 and TNF-α are not specific to cGAS-STING signaling, changes in a single downstream cytokine should not be considered sufficient evidence of pathway modulation. Composite biomarker panels combining target-proximal markers, transcriptional signatures, and disease-specific immune readouts are therefore required. Moreover, biomarker thresholds should be adapted to different diseases, tissues, and therapeutic directions rather than uniformly applied to cancer, infection, autoimmune disease, and organ injury.

At present, clinical translation of TCM interventions targeting the cGAS-STING pathway remains unproven, and no available clinical study has directly demonstrated pathway engagement using prespecified mechanistic endpoints. Early-phase clinical studies should therefore combine conventional safety and efficacy outcomes with baseline biomarker-based patient stratification, serial pharmacokinetic and pharmacodynamic measurements, and predefined criteria for target engagement. Such designs would help determine whether the observed clinical benefits are genuinely mediated by cGAS-STING regulation rather than by nonspecific anti-inflammatory, antioxidant, or immunomodulatory effects.

In summary, the cGAS-STING pathway provides a coherent molecular framework for modern research on TCM and serves as a key entry point for understanding its roles in immune regulation, anti-inflammation, anti-infection, and anticancer activity. Owing to its multi-component, multi-target, multi-pathway, and bidirectional regulatory nature, TCM offers abundant resources for the development of cGAS-STING-targeted strategies. Future breakthroughs will depend not on accumulating descriptive evidence, but on translating mechanistic insights into therapeutic strategies with defined targets, clear mechanisms, rational stratification, and clinical validation. With continued advances in target identification, human-relevant validation, multi-omics integration, and delivery technologies, TCM-based modulation of the cGAS-STING pathway may gradually evolve from a focus of basic research toward a more mechanism-defined and translationally testable therapeutic strategy.

## Data Availability

No datasets were generated or analysed during the current study.

## Abbreviations

cGAS: Cyclic GMP-AMP synthase
STING: Stimulator of interferon genes
TCM: Traditional Chinese Medicine
CHM: Chinese Herbal Medicine
IFN-γ: Interferon-γ
EMT: Epithelial-mesenchymal transition
IFN-I: Type I interferons
IL-6: Interleukin-6
dsDNA: Double-stranded DNA
ER: Endoplasmic reticulum
ERGIC: Endoplasmic reticulum-Golgi intermediate compartment
TBK1: TANK-binding kinase 1
IRF3: Interferon regulatory factor 3
IKK: Inhibitor of nuclear factor κB kinase
NF-κB: Nuclear factor κB
TNF-α: Tumor necrosis factor-α
IL-1β: Interleukin-1β
ISGs: Interferon-stimulated genes
SARS-CoV-2: Severe acute respiratory syndrome coronavirus 2
SLE: Systemic lupus erythematosus
NK: Natural killer
TREX1: Three prime repair exonuclease 1
LLF: Ligustri Lucidi Fructus
LONP1: Lon peptidase 1
HNK: Honokiol
TET: Tetrandrine
Cuc B: Cucurbitacin B
Lico A: Licochalcone A
NSCLC: Non-small cell lung cancer
γ-H2AX: Phosphorylated histone H2AX
Tregs: Regulatory T cells
MDSC: Myeloid-derived suppressor cell
PRDX1: Peroxiredoxin 1
PINK1: PTEN-induced kinase 1
mtDNA: Mitochondrial DNA
HSV-1: Herpes simplex virus type 1
IFN-β: Interferon-β
NPC1: Niemann-Pick disease type C1
VSV: Vesicular stomatitis virus
H1N1: Influenza A virus subtype H1N1
HBsAg: Hepatitis B surface antigen
HBV: Hepatitis B virus
p62: Sequestosome 1
iNOS: Inducible nitric oxide synthase
IL-10: Interleukin-10
IL-27: Interleukin-27
SIRT7: Sirtuin 7
PRMT5: Protein arginine methyltransferase 5
MAT2A: Methionine adenosyltransferase 2A
SAM: S-adenosylmethionine
NCOA4: Nuclear receptor coactivator 4
PI3K: Phosphoinositide 3-kinase
AKT: Protein kinase B
BZBS: Bazi Bushen Capsules
QLXZF: Qianlie Xiaozheng Formula
FWHJ: Feiwei Mixture
NR1D1: Nuclear receptor subfamily 1 group D member 1
JAK-STAT3: Janus kinase-signal transducer and activator of transcription 3
Lut: Luteolin
Sc: Schisandrin C
LWLT: Liuwei Wuling Tablet
EPE: Epimedium brevicornu Maxim. extract
STE: Sophora tonkinensis extract
cGAMP: Cyclic GMP-AMP
ADNVs: Artemisia-derived nanovesicles
M2: Alternatively activated macrophages
M1: Classically activated macrophages
QCT: Quercetin
MRI: Magnetic resonance imaging
DAMPs: Damage-associated molecular patterns
TME: Tumor microenvironment
CTL: Cytotoxic T lymphocyte
DCs: Dendritic cells
SDT: Sonodynamic therapy
IRIs: Implant-related infections
SJD: Shuangdan Jiedu Decoction
NLRP3: NOD-like receptor family pyrin domain containing 3
AIM2: Absent in melanoma 2
NLRC4: NLR family CARD domain-containing protein 4
YQP: Yinqiao Powder
TRQ: Tanreqing Injection
BIP: Binding immunoglobulin protein
PERK: Protein kinase RNA-like endoplasmic reticulum kinase
eIF2α: Eukaryotic initiation factor 2 alpha
ATF4: Activating transcription factor 4
QF: Qingfei Xieding prescription
BFD: Bu-fei Decoction
COPD: Chronic obstructive pulmonary disease
C1qbp: Complement component 1 q subcomponent-binding protein
YSXZF: Yi-Shen-Xie-Zhuo formula
AKI: Acute kidney injury
SQFZ: Shenqi Fuzheng Injection
YQJPXY: Yi-Qi-Jian-Pi-Xiao-Yu formula
NED: Nephropathy II Decoction
ZWD: Zhen Wu Decoction
NRF2: Nuclear factor erythroid 2-related factor 2
TFAM: Mitochondrial transcription factor A
QHYS: Qihuang Yishen formula
HG/PA: High glucose/palmitic acid
HDD: Huangqi-Danshen Decoction
SCD1: Stearoyl-CoA desaturase 1
PXR: Pregnane X receptor
LYN: LYN proto-oncogene, Src family tyrosine kinase
Nrf2: Nuclear factor erythroid 2-related factor 2
HO-1: Heme oxygenase-1
NQO1: NAD(P)H quinone dehydrogenase 1
MAFLD: Metabolic dysfunction-associated fatty liver disease
MASLD: Metabolic dysfunction-associated steatotic liver disease
RA: Rheumatoid arthritis
IBD: Inflammatory bowel disease
BTD: Bai Tong Tang
DSS: Dextran sulfate sodium
ROS: Reactive oxygen species
DAI: Disease activity index
AG&A: American ginseng-Achyranthes root
NOD: Non-obese diabetic
MDDST: Maidong Dishao Decoction
SSA: Sjögren's syndrome-related antigen A
IgG: Immunoglobulin G
CDDP: Compound Danshen Dripping Pills
SGD: Shaoyao Gancao Decoction
IL-12: Interleukin-12
ZXP: Zixue San
SNARE: Soluble N-ethylmaleimide-sensitive factor attachment protein receptor
NETs: Neutrophil extracellular traps
FEF: Fufang Epimedium Formula
ALT: Alanine aminotransferase
AST: Aspartate aminotransferase
ETD: ErTao Decoction
CCl₄: Carbon tetrachloride
LGZG: Lingguizhugan Decoction
NAFLD: Non-alcoholic fatty liver disease
CVDs: Cardiovascular diseases
MIRI: Myocardial ischemia–reperfusion injury
Ta-IIA: Tanshinone IIA
As-IV: Astragaloside IV
CHF: Chronic heart failure
XJEK: Xin-Ji-Er-Kang
NR3C1: Nuclear receptor subfamily 3 group C member 1
MFN2: Mitofusin 2
NT-proBNP: N-terminal pro-B-type natriuretic peptide
cTnI: Cardiac troponin I
DCM: Diabetic cardiomyopathy
TSD: Tao Hong Si Wu decoction
MDA: Malondialdehyde
SOD: Superoxide dismutase
AS: Atherosclerosis
KX: Kuanxiong Aerosol
CX3CR1: C-X3-C motif chemokine receptor 1
HUVECs: Human umbilical vein endothelial cells
LOX-1: Lectin-like oxidized low-density lipoprotein receptor-1
DZP: Dingzhen pills
PD: Parkinson's disease
BYHWD: Buyang Huanwu Decoction
OGD: Oxygen–glucose deprivation
2VO: Two-vessel occlusion
ZZHP: Zhi-Zi-Hou-Po decoction
JTW: Jiao-tai-wan
STZ: Streptozotocin
JTTZF: Jiangtang Tiaozhi Formula
SBMA: Sulfobetaine methacrylate
GelMA: Gelatin methacryloyl
PPARα: Peroxisome proliferator-activated receptor alpha
SHD: San Huang Decoction
DFUs: Diabetic foot ulcers
BMSCs: Bone marrow mesenchymal stem cells
HCC: Hepatocellular carcinoma

## References

[1] Zhang N, Wang M, Gao L, Zhang C, Tang X, Liu X, et al. Anti-HIV activity in traditional Chinese medicine: clinical implications of monomeric herbal remedies and compound decoctions. Front Med (Lausanne). 2024;11:1322870. 39175814 10.3389/fmed.2024.1322870 PMC11340536

[2] Jia J, Chen J, Wang G, Li M, Zheng Q, Li D. Progress of research into the pharmacological effect and clinical application of the traditional Chinese medicine Rehmanniae Radix. Biomed Pharmacother. 2023;168(2023):29. 10.1016/j.biopha.2023.115809 37907043

[3] Zhi H, Fu H, Zhang Y, Fan N, Zhao C, Li Y, et al. Progress of cGAS-STING signaling pathway-based modulation of immune response by traditional Chinese medicine in clinical diseases. Front Immunol. 2024;15:1510628. 39737190 10.3389/fimmu.2024.1510628 PMC11683013

[4] Xiang Y, Guo Z, Zhu P, Chen J, Huang Y. Traditional Chinese medicine as a cancer treatment: modern perspectives of ancient but advanced science. Cancer Med. 2019. 10.1002/cam4.2108. 30945475 PMC6536969

[5] Zhou J, Zhuang Z, Li J, Feng Z. Significance of the cGAS-STING pathway in health and disease. Int J Mol Sci. 2023. 10.3390/ijms241713316. 37686127 PMC10487967

[6] Shen M, Jiang X, Peng Q, Oyang L, Ren Z, Wang J, et al. The cGAS-STING pathway in cancer immunity: mechanisms, challenges, and therapeutic implications. J Hematol Oncol. 2025;18(1):40. 40188340 10.1186/s13045-025-01691-5 PMC11972543

[7] Lu C, Wang W, Fu YX. Opportunities and challenges of targeting cGAS-STING in cancer. Nat Rev Cancer. 2026;26(3):200–16. 41486397 10.1038/s41568-025-00894-9

[8] Wang Q, Yu Y, Zhuang J, Liu R, Sun C. Demystifying the cGAS-STING pathway: precision regulation in the tumor immune microenvironment. Mol Cancer. 2025;24(1):178. 40506729 10.1186/s12943-025-02380-0 PMC12160120

[9] Zhou J, Yang Y, Fang Y, Du X, Ying Z, Rao C. Targeting cGAS-STING signaling and cell death modes in cancer and autoimmune diseases. Cytokine Growth Factor Rev. 2025;85(2025):18. 10.1016/j.cytogfr.2025.07.004 40707310

[10] Zhang J, Zhang L, Chen Y, Fang X, Li B, Mo C. The role of cGAS-STING signaling in pulmonary fibrosis and its therapeutic potential. Front Immunol. 2023;14:1273248. 37965345 10.3389/fimmu.2023.1273248 PMC10642193

[11] Ablasser A, Chen ZJ. cGAS in action: Expanding roles in immunity and inflammation. Science. 2019;363(6431):eaat8657. 30846571 10.1126/science.aat8657

[12] Decout A, Katz JD, Venkatraman S, Ablasser A. The cGAS-STING pathway as a therapeutic target in inflammatory diseases. Nat Rev Immunol. 2021;21(9):548–69. 33833439 10.1038/s41577-021-00524-z PMC8029610

[13] Yu X, Cai L, Yao J, Li C, Wang X. Agonists and inhibitors of the cGAS-STING pathway. Molecules. 2024. 10.3390/molecules29133121. 38999073 PMC11243509

[14] Chen C, Xu P. Cellular functions of cGAS-STING signaling. Trends Cell Biol. 2023;33(8):630–48. 36437149 10.1016/j.tcb.2022.11.001

[15] Jiang M, Chen P, Wang L, Li W, Chen B, Liu Y, et al. cGAS-STING, an important pathway in cancer immunotherapy. J Hematol Oncol. 2020;13(1):81. 32571374 10.1186/s13045-020-00916-z PMC7310007

[16] Amouzegar A, Chelvanambi M, Filderman JN, Storkus WJ, Luke JJ. STING agonists as cancer therapeutics. Cancers (Basel). 2021. 10.3390/cancers13112695. 34070756 PMC8198217

[17] Domizio JD, Gulen MF, Saidoune F, Thacker VV, Yatim A, Sharma K, et al. The cGAS-STING pathway drives type I IFN immunopathology in COVID-19. Nature. 2022;603(7899):145–51. 35045565 10.1038/s41586-022-04421-w PMC8891013

[18] Haag SM, Gulen MF, Reymond L, Gibelin A, Abrami L, Decout A, et al. Targeting STING with covalent small-molecule inhibitors. Nature. 2018;559(7713):269–73. 29973723 10.1038/s41586-018-0287-8

[19] Li S, Mirlekar B, Johnson BM, Brickey WJ, Wrobel JA, Yang N, et al. STING-induced regulatory B cells compromise NK function in cancer immunity. Nature. 2022;610(7931):373–80. 36198789 10.1038/s41586-022-05254-3 PMC9875944

[20] Skopelja-Gardner S, An J, Elkon KB. Role of the cGAS-STING pathway in systemic and organ-specific diseases. Nat Rev Nephrol. 2022;18(9):558–72. 35732833 10.1038/s41581-022-00589-6 PMC9214686

[21] Hu J, Sánchez-Rivera FJ, Wang Z, Johnson GN, Ho YJ, Ganesh K, et al. STING inhibits the reactivation of dormant metastasis in lung adenocarcinoma. Nature. 2023;616(7958):806–13. 36991128 10.1038/s41586-023-05880-5 PMC10569211

[22] Tani T, Mathsyaraja H, Campisi M, Li ZH, Haratani K, Fahey CG, et al. TREX1 inactivation unleashes cancer cell STING-interferon signaling and promotes antitumor immunity. Cancer Discov. 2024;14(5):752–65. 38227896 10.1158/2159-8290.CD-23-0700 PMC11062818

[23] Zhao H, Wu L, Yan G, Chen Y, Zhou M, Wu Y, et al. Inflammation and tumor progression: signaling pathways and targeted intervention. Signal Transduct Target Ther. 2021;6(1):263. 34248142 10.1038/s41392-021-00658-5 PMC8273155

[24] Cao LL, Kagan JC. Targeting innate immune pathways for cancer immunotherapy. Immunity. 2023;56(10):2206–17. 37703879 10.1016/j.immuni.2023.07.018 PMC10591974

[25] Hopfner KP, Hornung V. Molecular mechanisms and cellular functions of cGAS-STING signalling. Nat Rev Mol Cell Biol. 2020;21(9):501–21. 32424334 10.1038/s41580-020-0244-x

[26] Ishikawa H, Ma Z, Barber GN. STING regulates intracellular DNA-mediated, type I interferon-dependent innate immunity. Nature. 2009;461(7265):788–92. 19776740 10.1038/nature08476 PMC4664154

[27] Liu J, Zhou J, Luan Y, Li X, Meng X, Liao W, et al. cGAS-STING, inflammasomes and pyroptosis: an overview of crosstalk mechanism of activation and regulation. Cell Commun Signal. 2024;22(1):22. 38195584 10.1186/s12964-023-01466-w PMC10775518

[28] Swanson KV, Deng M, Ting JP. The NLRP3 inflammasome: molecular activation and regulation to therapeutics. Nat Rev Immunol. 2019;19(8):477–89. 31036962 10.1038/s41577-019-0165-0 PMC7807242

[29] Lu Q, Chen Y, Li J, Zhu F, Zheng Z. Crosstalk between cGAS-STING pathway and autophagy in cancer immunity. Front Immunol. 2023;14:1139595. 36936940 10.3389/fimmu.2023.1139595 PMC10014609

[30] Zhang Z, Yang Z, Wang S, Wang X, Mao J. Ferroptosis and pyroptosis: mediating mechanisms of the cGAS/STING pathway and therapeutic strategies in heart failure. Ageing Res Rev. 2026;113:102932. 41202894 10.1016/j.arr.2025.102932

[31] Li T, Chen ZJ. The cGAS-cGAMP-STING pathway connects DNA damage to inflammation, senescence, and cancer. J Exp Med. 2018;215(5):1287–99. 29622565 10.1084/jem.20180139 PMC5940270

[32] Li C, Wen J, Zhan X, Shi W, Ye X, Yao Q, et al. Total tanshinones ameliorates cGAS-STING-mediated inflammatory and autoimmune diseases by affecting STING-IRF3 binding. Chin Med. 2024;19(1):107. 39148120 10.1186/s13020-024-00980-4 PMC11325629

[33] Long D, Mao C, Zhu Y, Xu Y. cGAS-STING pathway as a promising target for digestive diseases: insights from natural plant products. Front Nutr. 2025;12:1594120. 40621423 10.3389/fnut.2025.1594120 PMC12226309

[34] Liu M, Gu Y, Yang Y, Zhang K, Yang J, Wang W, et al. Network pharmacology, molecular dynamics simulation, and biological validation insights into the potential of *Ligustri Lucidi Fructus* for diabetic nephropathy. Int J Mol Sci. 2025. 10.3390/ijms26136303. 40650083 PMC12249564

[35] Cao L, Xu E, Zheng R, Zhangchen Z, Zhong R, Huang F, et al. Traditional Chinese medicine Lingguizhugan decoction ameliorate HFD-induced hepatic-lipid deposition in mice by inhibiting STING-mediated inflammation in macrophages. Chin Med. 2022;17(1):7. 34983596 10.1186/s13020-021-00559-3 PMC8728979

[36] Zhou Q, Wang K, Wang C, Sun X, Wang L, Sun J, et al. Wen-Shen-Tong-Luo-Zhi-Tong decoction alleviates bone loss in aged mice by suppressing LONP1-mediated macrophage senescence. Pharm Biol. 2025;63(1):524–48. 40719285 10.1080/13880209.2025.2537125 PMC12305870

[37] Sun L, Wu J, Du F, Chen X, Chen ZJ. Cyclic GMP-AMP synthase is a cytosolic DNA sensor that activates the type I interferon pathway. Science. 2013;339(6121):786–91. 23258413 10.1126/science.1232458 PMC3863629

[38] Wu J, Sun L, Chen X, Du F, Shi H, Chen C, et al. Cyclic GMP-AMP is an endogenous second messenger in innate immune signaling by cytosolic DNA. Science. 2013;339(6121):826–30. 23258412 10.1126/science.1229963 PMC3855410

[39] Corrales L, Gajewski TF. Molecular pathways: targeting the stimulator of interferon genes (STING) in the immunotherapy of cancer. Clin Cancer Res. 2015;21(21):4774–9. 26373573 10.1158/1078-0432.CCR-15-1362 PMC4750108

[40] Woo SR, Fuertes MB, Corrales L, Spranger S, Furdyna MJ, Leung MY, et al. STING-dependent cytosolic DNA sensing mediates innate immune recognition of immunogenic tumors. Immunity. 2014;41(5):830–42. 25517615 10.1016/j.immuni.2014.10.017 PMC4384884

[41] Deng L, Liang H, Xu M, Yang X, Burnette B, Arina A, et al. STING-dependent cytosolic DNA sensing promotes radiation-induced type I interferon-dependent antitumor immunity in immunogenic tumors. Immunity. 2014;41(5):843–52. 25517616 10.1016/j.immuni.2014.10.019 PMC5155593

[42] Corrales L, McWhirter SM, Dubensky TW Jr., Gajewski TF. The host STING pathway at the interface of cancer and immunity. J Clin Invest. 2016;126(7):2404–11. 27367184 10.1172/JCI86892 PMC4922692

[43] Yue B, Gao W, Lovell JF, Jin H, Huang J. The cGAS-STING pathway in cancer immunity: dual roles, therapeutic strategies, and clinical challenges. Essays Biochem. 2025. 10.1042/ebc20253006. 40052963 PMC12204001

[44] Sun P, Wang B, Liu C, Wang Z, Liu Y, Qiao YB, et al. A fluorescent STING ligand sensor for high-throughput screening of compounds that can enhance tumor immunotherapy. Cell Rep Methods. 2025;5(7):101106. 40669456 10.1016/j.crmeth.2025.101106 PMC12296492

[45] Zhu M, Tang X, Zhu Z, Gong Z, Tang W, Hu Y, et al. STING activation in macrophages by vanillic acid exhibits antineoplastic potential. Biochem Pharmacol. 2023;213:115618. 37211172 10.1016/j.bcp.2023.115618

[46] Bai B, Zhang L, Zhang Y, Yue R, Lu Y, Shi R, et al. Astragaloside IV potentiates cisplatin sensitivity in triple-negative breast cancer via STING signaling pathway activation. Phytomedicine. 2025;148:157330. 41016300 10.1016/j.phymed.2025.157330

[47] Tan Y, Zhu Q, Yang M, Yang F, Zeng Q, Jiang Z, et al. Tetrandrine activates STING/TBK1/IRF3 pathway to potentiate anti-PD-1 immunotherapy efficacy in non-small cell lung cancer. Pharmacol Res. 2024;207:107314. 39059614 10.1016/j.phrs.2024.107314

[48] Kang LP, Li NN, Chen PS, Chen HH, Huang DH, Jiang ZB. Plumbagin triggers STING pathway activation to suppress non-small cell lung cancer progression. Chem Biol Drug Des. 2025;106(4):e70176. 41065101 10.1111/cbdd.70176

[49] Luo B, Lu Q, Wang Q, Shen Y, Miao Y, Ma Y. Induction of the STING-dependent DNA damage pathway by cucurbitacin B enhances immunotherapy efficacy in osteosarcoma. Iran J Basic Med Sci. 2025;28(10):1319–27. 40896699 10.22038/ijbms.2025.86376.18663 PMC12399069

[50] Ge X, Yang GM, Zhang XL, Cao J, Qing YJ, Shen SB, et al. Isoliensinine inhibits mitophagy and sensitizes T cell malignancies for STING-mediated NK clearance. Acta Pharmacol Sin. 2026;47(1):242–54. 40841706 10.1038/s41401-025-01636-1 PMC12764981

[51] Xu Q, Jiang Z, Pan Y, Li S, Cao Z, Hua S, et al. Cucurbitacin B stimulates PD-1 immunotherapy response in malignant breast cancer by covalent targeting MTCH2. Phytomedicine. 2025;145:157017. 40582210 10.1016/j.phymed.2025.157017

[52] Li LG, Hu J, Han N, Chen NN, Yu TT, Ren T, et al. Dihydroartemisinin-driven TOM70 inhibition leads to mitochondrial destabilization to induce pyroptosis against lung cancer. Phytother Res. 2024;38(8):3856–76. 38761036 10.1002/ptr.8242

[53] Kang LP, Xu C, Xu P, Huang DH, Jiang ZB. Brazilin inhibits the proliferation of non-small cell lung cancer by regulating the STING/TBK1/IRF3 pathway. J Cell Mol Med. 2025;29(13):e70688. 40596709 10.1111/jcmm.70688 PMC12213455

[54] Jiang ZB, He QH, Kang LP, Jiang S, Liu JN, Xu C, et al. Rutecarpine suppresses non-small cell lung cancer progression through activating the STING pathway and elevating CD8+ T cells. Chem Biol Drug Des. 2025;105(2):e70070. 39989173 10.1111/cbdd.70070

[55] Wang Y, Fan N, Wang R, Li X, Zhao F, Miao L, et al. Discovery of airway-administered ionophores for Mn(2+) to mitigate lung metastasis by targeting disseminated tumor cell. ACS Nano. 2025;19(14):14330–50. 40178871 10.1021/acsnano.5c01732

[56] Deng L, Wei T, Zhang Y, Shen A, He X, Gao S, et al. Ultra-pH-sensitive nanoparticle of gambogenic acid for tumor targeting therapy via anti-vascular strategy plus immunotherapy. Int J Pharm. 2024;660:124303. 38848801 10.1016/j.ijpharm.2024.124303

[57] Huang MY, Xu CC, Chen Q, Zhang YM, Lyu WY, Ye ZH, et al. Ginsenoside Rh2 in combination with IFNγ potentiated the anti-cancer effect by enhancing interferon signaling response in colorectal cancer cells. Acta Pharmacol Sin. 2025;46(9):2534–46. 40263567 10.1038/s41401-025-01557-z PMC12373737

[58] Wang Y, Li F, Wang Z, Song X, Ren Z, Wang X, et al. Luteolin inhibits herpes simplex virus 1 infection by activating cyclic guanosine monophosphate-adenosine monophosphate synthase-mediated antiviral innate immunity. Phytomedicine. 2023;120:155020. 37632997 10.1016/j.phymed.2023.155020

[59] Qi H, Ma QH, Feng W, Chen SM, Wu CS, Wang Y, et al. Glycyrrhetinic acid blocks SARS-CoV-2 infection by activating the cGAS-STING signalling pathway. Br J Pharmacol. 2024;181(20):3976–92. 38922702 10.1111/bph.16473

[60] Cheng C, Wang Y, Wang H, Zhang M, Li Q, Xu B, et al. Phytochemical alkaloids orchestrate immunometabolism against viral infections. Natl Sci Rev. 2025;12(9):nwaf190. 40927436 10.1093/nsr/nwaf190 PMC12416277

[61] Zhao J, Xu G, Hou X, Mu W, Yang H, Shi W, et al. Schisandrin C enhances cGAS-STING pathway activation and inhibits HBV replication. J Ethnopharmacol. 2023;311:116427. 37001770 10.1016/j.jep.2023.116427

[62] Xie J, Shang L, Liu C, Mao J, He C, Luo M, et al. Corilagin inhibits human cytomegalovirus infection and replication via activating the cGAS-STING signaling pathway in vitro and in vivo. Int Immunopharmacol. 2024;143(Pt 2):113401. 39423664 10.1016/j.intimp.2024.113401

[63] Liu Y, Tang Q, Rao Z, Fang Y, Jiang X, Liu W, et al. Inhibition of herpes simplex virus 1 by cepharanthine via promoting cellular autophagy through up-regulation of STING/TBK1/P62 pathway. Antiviral Res. 2021;193:105143. 34303748 10.1016/j.antiviral.2021.105143

[64] J. Chen, H. Du, S. Cui, T. Liu, G. Yang, H. Sun, W. Tao, B. Jiang, L. Yu, F. You, E. fischeriana Root Compound Dpo Activates Antiviral Innate Immunity, Front Cell Infect Microbiol 7 (2017) 456. 10.3389/fcimb.2017.00456 PMC5662903 29124042

[65] Wang G, Liu J, Zhang Y, Xie J, Chen S, Shi Y, et al. Ginsenoside Rg3 enriches SCFA-producing commensal bacteria to confer protection against enteric viral infection via the cGAS-STING-type I IFN axis. ISME J. 2023;17(12):2426–40. 37950067 10.1038/s41396-023-01541-7 PMC10689736

[66] Guo Y, Wen J, Wang X, Wang X, Xu Y, Huang S, et al. *Sophora tonkinensis* enhances activation of cGAS-STING pathway and restrains HBV replication. Front Pharmacol. 2025;16:1630460. 41158134 10.3389/fphar.2025.1630460 PMC12554753

[67] Jin X, Jiang W, Zhao X, Wang W, Liu T, Shao Q, et al. Efficacy and mechanism of topical Chinese herbal formulation JieZe-1 on female lower genital tract co-infection with HSV-2 and *Candida albicans* via upregulating the cGAS/STING/IFN-I pathway. J Ethnopharmacol. 2026;368:121842. 42114577 10.1016/j.jep.2026.121842

[68] Qu J, Li J, Wang L, Li Y, Zhang Y, Li J, et al. Disturbance of cytoskeleton induced by ligustilide promotes hepatic stellate cell senescence and ameliorates liver fibrosis. Theranostics. 2025;15(16):8049–67. 40860128 10.7150/thno.108869 PMC12374544

[69] Wang J, Tian H, Gao Y, Qiu X, Bao Z, Zhao D, et al. Dual roles of SIRT7 inhibition by oroxylin A reprogram HSCs fate: PRMT5 succinylation-driven senescence and ecto-calreticulin-dependent NK cell immune clearance in liver fibrosis. Research (Wash D C). 2025;8:0808. 40777599 10.34133/research.0808 PMC12329212

[70] Zhao D, Gao Y, Su Y, Zhou Y, Yang T, Li Y, et al. Oroxylin A regulates cGAS DNA hypermethylation induced by methionine metabolism to promote HSC senescence. Pharmacol Res. 2023;187:106590. 36464146 10.1016/j.phrs.2022.106590

[71] Sun Y, Weng J, Chen X, Ma S, Zhang Y, Zhang F, et al. Oroxylin A activates ferritinophagy to induce hepatic stellate cell senescence against hepatic fibrosis by regulating cGAS-STING pathway. Biomed Pharmacother. 2023;162:114653. 37086511 10.1016/j.biopha.2023.114653

[72] Wang SS, Jin X, Ma WD, Li WY, Dou MM, Yan TY, et al. Oxymatrine regulates microglia to produce IFN-β by activating the STING/TBK1/IRF3 pathway against experimental autoimmune encephalomyelitis. Eur J Pharmacol. 2025;1009:178380. 41265623 10.1016/j.ejphar.2025.178380

[73] Xu A, Zhang Z, Zhu B, Xiao M, Jiang X, Yang Y, et al. Shaoyao Gancao decoction promotes splenic immune homeostasis mediating cGAS-STING signaling pathway to delay autoimmune hepatitis progression. J Ethnopharmacol. 2025;352:120121. 40523449 10.1016/j.jep.2025.120121

[74] Gu S, Wu G, Lu D, Wang Y, Tang L, Zhang W. Human kidney organoids model of Esculentoside A nephrotoxicity to investigate the role of epithelial-mesenchymal transition via STING signaling. Toxicol Lett. 2023;373:172–83. 36460195 10.1016/j.toxlet.2022.11.019

[75] Cheng SY, Yang YF, Wang YL, Yue ZP, Chen YZ, Wang WK, et al. Triptolide exposure triggers ovarian inflammation by activating cGAS-STING pathway and decrease oocyte quality in mouse. Food Chem Toxicol. 2025;196:115201. 39672454 10.1016/j.fct.2024.115201

[76] Liu R, Yue Z, Dong J, Zhang C, Guo C, Wang X. Dihydromyricetin (DHM) inhibits microglial pyroptosis and oxidative stress after spinal cord injury by promoting STING-mediated autophagy. Biochem Genet. 2025. 10.1007/s10528-025-11217-w. 40731204

[77] Liu Y, Jesus AA, Marrero B, Yang D, Ramsey SE, Sanchez GAM, et al. Activated STING in a vascular and pulmonary syndrome. N Engl J Med. 2014;371(6):507–18. 25029335 10.1056/NEJMoa1312625 PMC4174543

[78] Yan H, Huang J, Wang Y, Zhang Y, Ren W, Zhai Z, et al. Berbamine as potential STING inhibitor for KRAS-mutant non-small cell lung cancer. Pharmacol Res. 2025;216:107777. 40383171 10.1016/j.phrs.2025.107777

[79] Tang X, Zhu M, Zhu Z, Tang W, Zhang H, Chen Y, et al. Ginsenoside Re inhibits non-small cell lung cancer progression by suppressing macrophage M2 polarization induced by AMPKα1/STING positive feedback loop. Phytother Res. 2024;38(11):5088–106. 39119862 10.1002/ptr.8309

[80] Tu S, Mao D, Shi M, Zhang H, Liu C, Li X, et al. Icaritin ameliorates extracellular microparticles-induced inflammatory pre-metastatic niche via modulating the cGAS-STING signaling. Phytother Res. 2022;36(5):2127–42. 35257426 10.1002/ptr.7433

[81] Liu H, Wang Z, Liu Z. Formononetin restrains tumorigenesis of breast tumor by restraining STING-NF-κB and interfering with the activation of PD-L1. Discov Med. 2024;36(182):613–20. 38531802 10.24976/Discov.Med.202436182.58

[82] Wei J, Liu Z, Sun H, Xu L. Perillaldehyde ameliorates lipopolysaccharide-induced acute lung injury via suppressing the cGAS/STING signaling pathway. Int Immunopharmacol. 2024;130:111641. 38368770 10.1016/j.intimp.2024.111641

[83] Zhou XW, Wang J, Tan WF. Apigenin suppresses innate immune responses and ameliorates lipopolysaccharide-induced inflammation via inhibition of STING/IRF3 pathway. Am J Chin Med. 2024;52(2):471–92. 38480499 10.1142/S0192415X24500204

[84] He YQ, Deng JL, Zhou CC, Jiang SG, Zhang F, Tao X, et al. Ursodeoxycholic acid alleviates sepsis-induced lung injury by blocking PANoptosis via STING pathway. Int Immunopharmacol. 2023;125(Pt B):111161. 37948864 10.1016/j.intimp.2023.111161

[85] He YQ, Zhou CC, Deng JL, Wang L, Chen WS. Tanreqing inhibits LPS-induced acute lung injury in vivo and in vitro through downregulating STING signaling pathway. Front Pharmacol. 2021;12:746964. 34721036 10.3389/fphar.2021.746964 PMC8552121

[86] Chen Z, Wang W, Zeng K, Zhu J, Wang X, Huang W. Potential antiviral activity of rhamnocitrin against influenza virus H3N2 by inhibiting cGAS/STING pathway in vitro. Sci Rep. 2024;14(1):28287. 39550441 10.1038/s41598-024-79788-z PMC11569172

[87] Xu J, Tao L, Zhang M, Wang S. Panaxadiol acts as an HIF-1α inhibitor to suppress H9N2-induced inflammation. Vet Microbiol. 2025;309:110695. 40897026 10.1016/j.vetmic.2025.110695

[88] Niu Y, Liu M, Cui S, Liu K, Yang M, Hu X, et al. Integrative network pharmacology and multi-omics analysis reveal key targets and mechanisms of Saikosaponin B1 against acute lung injury. Metabolites. 2025. 10.3390/metabo15120782. 41441024 PMC12735089

[89] Ding R, Xie H, Zhang Y, Qin L, Peng G, Yi J, et al. Ginsenoside Rg1 ameliorates pulmonary hypertension by inhibiting cGAS/STING mediated cell senescence. Drug Des Devel Ther. 2025;19:6487–504. 40756267 10.2147/DDDT.S527938 PMC12317700

[90] Ren G, Lv W, Ding Y, Wang L, Cui Z, Li R, et al. Ginseng saponin metabolite 20(S)-protopanaxadiol relieves pulmonary fibrosis by multiple-targets signaling pathways. J Ginseng Res. 2023;47(4):543–51. 37397411 10.1016/j.jgr.2023.01.002 PMC10310916

[91] Ruan Y, Ren G, Wang M, Lv W, Shimizu K, Zhang C. The dual role of 20(S)-protopanaxadiol in alleviating pulmonary fibrosis through the gut-lung axis. Phytomedicine. 2024;129:155699. 38733907 10.1016/j.phymed.2024.155699

[92] Yan X, Luo J, Chen X, Wang L, Xu Z, Guo C, et al. 6-Shogaol reduces renal macrophage infiltration by targeting the STING pathway to alleviate cisplatin induced renal injury. Phytother Res. 2025;39(9):4081–93. 40728195 10.1002/ptr.70053

[93] Wang Y, Liu S, Zhu F, Wang X, Wang H, Long L, et al. Improved bioavailability and anti-nephrotoxicity efficacy of polydatin on cisplatin-induced AKI via a dual-targeting fucoidan delivery system. Int J Pharm X. 2025;10:100422. 41189670 10.1016/j.ijpx.2025.100422 PMC12581698

[94] Liu S, Gao X, Yin Y, Wang J, Dong K, Shi D, et al. Silk fibroin peptide self-assembled nanofibers delivered naringenin to alleviate cisplatin-induced acute kidney injury by inhibiting mtDNA-cGAS-STING pathway. Food Chem Toxicol. 2023;177:113844. 37244599 10.1016/j.fct.2023.113844

[95] Liu S, Gao X, Wang Y, Wang J, Qi X, Dong K, et al. Baicalein-loaded silk fibroin peptide nanofibers protect against cisplatin-induced acute kidney injury: fabrication, characterization and mechanism. Int J Pharm. 2022;626:122161. 36058409 10.1016/j.ijpharm.2022.122161

[96] Jin X, He T, Zhang T, Wang X, Chen X, Cong B, et al. Research on Sinomenine inhibiting the cGAS-STING signaling pathway to alleviate renal inflammatory injury in db/db mice. Pharmaceuticals (Basel). 2025. 10.3390/ph18070934. 40732224 PMC12299331

[97] Wang Y, He X, Xue M, Sun W, He Q, Jin J. Germacrone protects renal tubular cells against ferroptotic death and ROS release by re-activating mitophagy in diabetic nephropathy. Free Radic Res. 2023;57(6–12):413–29. 37897414 10.1080/10715762.2023.2277143

[98] Gao Y, Wei T, Mu L, Liu C, Zeng Y, Guo X, et al. Targeting STING and protecting mitochondrial function with Nephropathy II decoction to alleviate renal fibrosis. Phytomedicine. 2025;142:156785. 40279968 10.1016/j.phymed.2025.156785

[99] Gu L, Wang F, Wang Y, Sun D, Sun Y, Tian T, et al. Naringin protects against inflammation and apoptosis induced by intestinal ischemia-reperfusion injury through deactivation of cGAS-STING signaling pathway. Phytother Res. 2023;37(8):3495–507. 37125528 10.1002/ptr.7824

[100] Xie W, Yang T, Mo F, Meng W, Xu L, Liang J, et al. Activating PXR by Curcumol ameliorates DSS-induced ulcerative colitis via transcriptional repression of STING signaling. J Agric Food Chem. 2026;74(7):6193–207. 41672771 10.1021/acs.jafc.5c11938

[101] Yang S, Liu T, Yang J, Qiao M, Meng X, Shen X, et al. Toosendanin inhibits STING signaling pathway to alleviate ulcerative colitis via blocking LYN kinase and STING protein. Biochem Pharmacol. 2026;243(Pt 1):117516. 41187884 10.1016/j.bcp.2025.117516

[102] Lai Z, Zhang Y, Huang W, Wang J, Liu C, Ren M, et al. Brusatol ameliorates irinotecan-induced delayed diarrhea via inhibition of the cGAS-STING pathway and modulation of intestinal flora. Phytomedicine. 2025;145:157071. 40684488 10.1016/j.phymed.2025.157071

[103] Wang Y, Wei B, Wang D, Wu J, Gao J, Zhong H, et al. DNA damage repair promotion in colonic epithelial cells by andrographolide downregulated cGAS-STING pathway activation and contributed to the relief of CPT-11-induced intestinal mucositis. Acta Pharm Sin B. 2022;12(1):262–73. 35127384 10.1016/j.apsb.2021.03.043 PMC8799857

[104] Chen L, Xia S, Wang S, Zhou Y, Wang F, Li Z, et al. Naringenin is a potential immunomodulator for inhibiting liver fibrosis by inhibiting the cGAS-STING pathway. J Clin Transl Hepatol. 2023;11(1):26–37. 36406329 10.14218/JCTH.2022.00120 PMC9647116

[105] Li Y, Yu P, Fu W, Wang S, Zhao W, Ma Y, et al. Ginsenoside Rd inhibited ferroptosis to alleviate CCl(4)-induced acute liver injury in mice via cGAS/STING pathway. Am J Chin Med. 2023;51(1):91–105. 36437551 10.1142/S0192415X23500064

[106] Wang HF, Xu JS, Zong K, Liang ZW, Li RF, Xue JF, et al. Jujuboside B alleviates acetaminophen-induced hepatotoxicity in mice by regulating Nrf2-STING signaling pathway. Ecotoxicol Environ Saf. 2024;269:115810. 38100849 10.1016/j.ecoenv.2023.115810

[107] Shen P, Han L, Chen G, Cheng Z, Liu Q. Emodin attenuates acetaminophen-induced hepatotoxicity via the cGAS-STING pathway. Inflammation. 2022;45(1):74–87. 34409550 10.1007/s10753-021-01529-5

[108] Wang Z, Liu J, Mou Y, Zhou X, Liao W, Li Y, et al. Disruption of cholesterol homeostasis triggers NLRP3-cGAS-STING axis-dependent hepatic fibrosis and honokiol intervention effects. Phytomedicine. 2025;143:156904. 40449452 10.1016/j.phymed.2025.156904

[109] Han S, Ma Q, Huang H, Cai F, Chen Q, Tian J, et al. Puerarin from Pueraria montana var lobate. (Willd.) alleviates hepatic steatosis and inflammation in MAFLD through suppressing STING-IRF3/NF-κB signaling in macrophages. J Ethnopharmacol. 2026;360:121126. 41478535 10.1016/j.jep.2025.121126

[110] Ding T, Shen W, Tao W, Peng J, Pan M, Qi X, et al. Curcumol ameliorates alcohol and high-fat diet-induced fatty liver disease via modulation of the Ceruloplasmin/iron overload/mtDNA signaling pathway. J Nutr Biochem. 2025;136:109807. 39549858 10.1016/j.jnutbio.2024.109807

[111] Qi X, Zheng S, Ma M, Lian N, Wang H, Chen L, et al. Curcumol suppresses CCF-mediated hepatocyte senescence through blocking LC3B-lamin B1 interaction in alcoholic fatty liver disease. Front Pharmacol. 2022;13:912825. 35837283 10.3389/fphar.2022.912825 PMC9273900

[112] Xie X, Liao Y, Lin Z, Luo H, Wei G, Huang N, et al. Patchouli alcohol alleviates metabolic dysfunction-associated steatohepatitis via inhibiting mitochondria-associated endoplasmic reticulum membrane disruption-induced hepatic steatosis and inflammation in rats. Int Immunopharmacol. 2024;138:112634. 38971107 10.1016/j.intimp.2024.112634

[113] Ji J, Li Y, Xu T, Shao Q, Sun Z, Chen S, et al. Protective effects of berberine on MASLD: regulation of glucose and lipid metabolism through PI3K/Akt and STING pathways. Naunyn Schmiedebergs Arch Pharmacol. 2025;398(9):12405–20. 40146248 10.1007/s00210-025-04077-z

[114] Lu M, Dai Z, Lin Y, Sun C, Li S, Guo Z, et al. Co-assembled nanoparticles comprising sorafenib and hederagenin derivative for enhanced anti-hepatic fibrosis activity. Int J Nanomedicine. 2025;20:9135–53. 40703473 10.2147/IJN.S512005 PMC12283418

[115] Hernández-Aquino E, Muriel P. Beneficial effects of naringenin in liver diseases: molecular mechanisms. World J Gastroenterol. 2018;24(16):1679–707. 29713125 10.3748/wjg.v24.i16.1679 PMC5922990

[116] Chen H, Lu X, Xu B, Cheng G, Li Y, Xie D. Saikosaponin d protects pancreatic acinar cells against cerulein-induced pyroptosis through alleviating mitochondrial damage and inhibiting cGAS-STING pathway. J Appl Toxicol. 2024;44(7):1005–13. 38462915 10.1002/jat.4594

[117] Jia H, Li J, Chen X, Liu Z, Wu C, Liu C, et al. ErTao decoction alleviates liver fibrosis by suppressing STING-mediated macrophages and NLRP3 inflammasome activation. Phytomedicine. 2025;139:156489. 39954622 10.1016/j.phymed.2025.156489

[118] Qin X, Zhang L, Tang J, Zhang F, Zhang L, Yue M, et al. Salvianolic acid A ameliorates sepsis through inhibiting inflammation via binding STING and modulating TBK1/IRF3 signaling pathway. Int Immunopharmacol. 2025;162:115104. 40570564 10.1016/j.intimp.2025.115104

[119] Liu Y, Ye J, Fan Z, Wu X, Zhang Y, Yang R, et al. Ginkgetin alleviates inflammation and senescence by targeting STING. Adv Sci. 2025;12(2):e2407222. 10.1002/advs.202407222 PMC11727237 39558862

[120] Mu W, Xu G, Li L, Wen J, Xiu Y, Zhao J, et al. Carnosic acid directly targets STING C-terminal tail to improve STING-mediated inflammatory diseases. Adv Sci. 2025;12(14):e2417686. 10.1002/advs.202417686 PMC11984877 39965124

[121] Zhang Y, Liu Y, Jiang B, Chen L, Hu J, Niu B, et al. Targeting STING oligomerization with licochalcone D ameliorates STING-driven inflammatory diseases. Sci China Life Sci. 2024;67(12):2664–77. 39190126 10.1007/s11427-024-2703-6

[122] Liu L, Wang Z, Qi Y, Huang A, He C, Zhan X, et al. Kurarinone alleviates cGAS-STING-triggered inflammatory diseases by targeting STING. Phytother Res. 2025;39(8):3450–65. 40604353 10.1002/ptr.70023

[123] Shi W, Wen J, Gao Y, Liu T, Li H, Yang H, et al. Dihydrotanshinone I, a main compound of *Salvia miltiorrhiza*, alleviates autoimmune and inflammatory diseases by directly targeting IRF3. Phytomedicine. 2025;145:157005. 40609382 10.1016/j.phymed.2025.157005

[124] Luo W, Song Z, Xu G, Wang H, Mu W, Wen J, et al. LicochalconeB inhibits cGAS-STING signaling pathway and prevents autoimmunity diseases. Int Immunopharmacol. 2024;128:111550. 38232536 10.1016/j.intimp.2024.111550

[125] Wen J, Mu W, Li H, Yan Y, Zhan X, Luo W, et al. Glabridin improves autoimmune disease in Trex1-deficient mice by reducing type I interferon production. Mol Med. 2023;29(1):167. 38066431 10.1186/s10020-023-00754-y PMC10709943

[126] Yang D, Peng N, Zhang H, Qiu Z, Xu L, Pan M. Cordycepin ameliorates autoimmunity by promoting STING degradation via autophagy pathway. Br J Pharmacol. 2025;182(7):1546–60. 39675775 10.1111/bph.17425

[127] Chu L, Li C, Li Y, Yu Q, Yu H, Li C, et al. Perillaldehyde inhibition of cGAS reduces dsDNA-induced interferon response. Front Immunol. 2021;12:655637. 33968056 10.3389/fimmu.2021.655637 PMC8100446

[128] Huang K, Xiao JL, Tang JY, Pu HY, Li FH, Gao K, et al. Chlorogenic acid targets STING to alleviate pressure overload-induced heart failure and myocardial fibrosis. Phytomedicine. 2026;150:157625. 41338109 10.1016/j.phymed.2025.157625

[129] Yang N, Yu G, Liu T, Dang Y, Deng P, Lei Z, et al. Direct inhibition of macrophage STING signaling by curcumol protects against myocardial infarction via attenuating the inflammatory response. Phytomedicine. 2025;138:156403. 39889491 10.1016/j.phymed.2025.156403

[130] Wang Y, Tang X, Cui J, Wang P, Yang Q, Chen Y, et al. Ginsenoside Rb1 mitigates acute catecholamine surge-induced myocardial injuries in part by suppressing STING-mediated macrophage activation. Biomed Pharmacother. 2024;175:116794. 38776673 10.1016/j.biopha.2024.116794

[131] Song J, Wang M, Li Q, Zhao W, Chen X, Li C, et al. Mangiferin inhibits cGAS-STING pathway-related inflammation via Nrf2 activation to protect against sepsis-induced heart injury. Chin Med. 2026;21(1):47. 41572319 10.1186/s13020-026-01329-9 PMC12829080

[132] Zhang J, Qi S, Du Y, Dai H, Liu N. Effect of quercetin on inhibiting gefitinib-activated non-small cell lung cancer-induced cell pyroptosis in cardiomyocytes via modulating mitochondrial autophagy mediated by the SHP2/ROS/AMPK/XBP-1/DJ-1 signaling pathway. Oncol Rep. 2025;53(5):57. 40183386 10.3892/or.2025.8890 PMC11976366

[133] Zhang R, Wu J, Wang D, Li K, Hou Y, Yu Y, et al. Xin-Ji-Er-Kang alleviates chronic heart failure by suppressing mtDNA/cGAS-STING signaling through NR3C1-mediated MFN2 upregulation. Phytomedicine. 2026;150:157714. 41422729 10.1016/j.phymed.2025.157714

[134] Li F, Zhou Y, Liu B, Du Z, Huang W. Epigallocatechin gallate-mediated inhibition of mitochondrial DNA sensing regulated by TBK1/cGAS/STING and NLRP3 alleviates cardiovascular toxicity in atherosclerosis. Int Immunopharmacol. 2025;164:115375. 40840141 10.1016/j.intimp.2025.115375

[135] Li W, Huang Z, Luo Y, Cui Y, Xu M, Luo W, et al. Tetrandrine alleviates atherosclerosis via inhibition of STING-TBK1 pathway and inflammation in macrophages. Int Immunopharmacol. 2023;119:110139. 37099944 10.1016/j.intimp.2023.110139

[136] Zhai H, Wang D, Wang Y, Gu H, Jv J, Yuan L, Wang C, Chen L. Kaempferol alleviates adipose tissue inflammation and insulin resistance in db/db mice by inhibiting the STING/NLRP3 signaling pathway. Endocr Connect. 2024; 13(5). 10.1530/EC-23-0379 PMC11046349 38466634

[137] Wu R, Liu R, Chen R, Li Y, Xue X, Zhang Y, et al. Aurantio-obtusin improves obesity and protects hepatic inflammation by rescuing mitochondrial damage in overwhelmed brown adipose tissue. Chin Med. 2025;20(1):41. 40133913 10.1186/s13020-025-01097-y PMC11934537

[138] Rui W, Chen Z, Tao S, Yao X, Xie W, Qin D, et al. Protocatechuic acid alleviates cGAS-STING-mediated neuroinflammation by enhancing microglial autophagy in mouse models of Parkinson’s disease. Phytomedicine. 2025;148:157493. 41218352 10.1016/j.phymed.2025.157493

[139] Chen P, Zhang Z, Lei J, Zhu J, Liu G. Ellagitannin component punicalin ameliorates cognitive dysfunction, oxidative stress, and neuroinflammation via the inhibition of cGAS-STING signaling in the brain of an aging mouse model. Phytother Res. 2024;38(12):5690–712. 39313488 10.1002/ptr.8343

[140] Cui Y, Zhi SM, Ding PF, Zhu T, Chen XX, Liu XZ, et al. Silybin attenuates microglia-mediated neuroinflammation via inhibition of STING in experimental subarachnoid hemorrhage. Int Immunopharmacol. 2025;151:114305. 39986195 10.1016/j.intimp.2025.114305

[141] Wei H, Chen Y, Qin Z, Wang H, Liu Y, Song T, et al. Artesunate demonstrates neuroprotective effect through activation of lysosomal function and inhibition of cGAS-STING pathway. Neuropharmacology. 2025;272:110426. 40118208 10.1016/j.neuropharm.2025.110426

[142] Song C, Yin G, Wang L, Zhang F. Platycodin D attenuates behavioral deficits, amyloid-β accumulation and mitochondrial impairment in AD models by inhibiting the cGAS-STING pathway. Neuromolecular Med. 2025;27(1):55. 40751867 10.1007/s12017-025-08878-6

[143] Gao D, Hao JP, Li BY, Zheng CC, Miao BB, Zhang L, et al. Tetrahydroxy stilbene glycoside ameliorates neuroinflammation for Alzheimer’s disease via cGAS-STING. Eur J Pharmacol. 2023;953:175809. 37328043 10.1016/j.ejphar.2023.175809

[144] Liu X, Chen W, Wang C, Liu W, Hayashi T, Mizuno K, et al. Silibinin ameliorates depression/anxiety-like behaviors of Parkinson’s disease mouse model and is associated with attenuated STING-IRF3-IFN-β pathway activation and neuroinflammation. Physiol Behav. 2021;241:113593. 34536434 10.1016/j.physbeh.2021.113593

[145] Li X, Zhang X, Yuan X, Yu H, Huang C, Liu K, et al. Echinacoside mitigates sepsis-associated encephalopathy by inhibiting STING pathway and reducing neuroinflammation. Eur J Pharmacol. 2025;1005:178092. 40885235 10.1016/j.ejphar.2025.178092

[146] Hu E, Wei Y, Liao T, Liu D, Gong Z, Mao J, et al. Quercetin alleviates osteoarthritis pain by inhibiting vascular endothelial growth factor A through regulating cGAS/STING pathway. J Cell Mol Med. 2026;30(1):e70992. 41474220 10.1111/jcmm.70992 PMC12755058

[147] Xu X, Zhang H, Chang A, Peng H, Li S, Zhang K, et al. Astilbin alleviates IL-17-induced hyperproliferation and inflammation in HaCaT cells via inhibiting ferroptosis through the cGAS-STING pathway. Int J Mol Sci. 2025. 10.3390/ijms26115075. 40507886 PMC12155464

[148] Li Y, Liu H, Zeng Z, Lin H, Chen X, Yuan X, et al. Ginsenoside Rb3 attenuates skin flap ischemia-reperfusion damage by inhibiting STING-IRF3 signaling. J Mol Histol. 2022;53(4):763–72. 35732862 10.1007/s10735-022-10081-x

[149] Qiu W, Zheng Z, Wang J, Cai Y, Zou J, Huang Z, et al. Targeting mitochondrial DNA-STING-NF-κB axis-mediated microglia activation by cryptotanshinone alleviates ischemic retinopathy. Phytomedicine. 2025;142:156779. 40279967 10.1016/j.phymed.2025.156779

[150] Jia ZC, Liu SJ, Chen TF, Shi ZZ, Li XL, Gao ZW, et al. Chlorogenic acid can improve spermatogenic dysfunction in rats with varicocele by regulating mitochondrial homeostasis and inhibiting the activation of NLRP3 inflammasomes by oxidative mitochondrial DNA and cGAS/STING pathway. Bioorg Chem. 2024;150:107571. 38936048 10.1016/j.bioorg.2024.107571

[151] Li M, Niu Y, Tian L, Zhang T, Zhou S, Wang L, et al. Astragaloside IV alleviates macrophage senescence and d-galactose-induced bone loss in mice through STING/NF-κB pathway. Int Immunopharmacol. 2024;129:111588. 38290207 10.1016/j.intimp.2024.111588

[152] Ling CQ, Yue XQ, Ling C. Three advantages of using traditional Chinese medicine to prevent and treat tumor. J Integr Med. 2014;12(4):331–5. 25074882 10.1016/S2095-4964(14)60038-8

[153] Xing F, Lv H, Xiang W, Wang L, Zong Q, Lv G, et al. Traditional medicine Bazi Bushen potentiates immunosurveillance of senescent liver cancer cells via cGAS-STING signaling activation in macrophages. Cancer Lett. 2025;627:217544. 39929434 10.1016/j.canlet.2025.217544

[154] Zhang X, Yuan G, Chen Y, Xu Y, Liu T, Zhao Q, et al. Qianlie Xiaozheng formula inhibits prostate cancer via the STING/TBK1/IRF3 pathway by suppressing CHK1. Adv Biol. 2025;9(12):e00246. 10.1002/adbi.202500246 41025394

[155] Wang H, Guo L, Zhao Y, Ye J, Zhao T, Zhang C. Feiwei mixture exerts antitumor activity against non-small cell lung cancer via regulating NR1D1-mediated immune cell infiltration. Adv Biol. 2026;10(2):e00574. 10.1002/adbi.202500574 41725319

[156] Wu Z, Zhao X, Li R, Wen X, Xiu Y, Long M, et al. The combination of Schisandrin C and Luteolin synergistically attenuates hepatitis B virus infection via repressing HBV replication and promoting cGAS-STING pathway activation in macrophages. Chin Med. 2024;19(1):48. 38500179 10.1186/s13020-024-00888-z PMC10946137

[157] Yao Q, Wen J, Chen S, Wang Y, Wen X, Wang X, et al. Shuangdan Jiedu Decoction improved LPS-induced acute lung injury by regulating both cGAS-STING pathway and inflammasome. J Ethnopharmacol. 2025;336:118661. 39159837 10.1016/j.jep.2024.118661

[158] Li H, Wen J, Cui Y, Wang S, Shi W, Yang H, et al. Yinqiao powder effectively alleviates acute lung injury via regulating cGAS-STING signaling pathway. Front Med. 2025. 10.1007/s11684-025-1172-0. 41454071

[159] Deng J, He Y, Sun G, Yang H, Wang L, Tao X, et al. Tanreqing injection protects against bleomycin-induced pulmonary fibrosis via inhibiting STING-mediated endoplasmic reticulum stress signaling pathway. J Ethnopharmacol. 2023;305:116071. 36584920 10.1016/j.jep.2022.116071

[160] Wang Y, He X, Wang H, Hu W, Sun L. Qingfei xieding prescription ameliorates mitochondrial DNA-initiated inflammation in bleomycin-induced pulmonary fibrosis through activating autophagy. J Ethnopharmacol. 2024;325:117820. 38286157 10.1016/j.jep.2024.117820

[161] Xu G, Su J, Hong Y, Zhang E, Zheng Z, Li J, et al. Bu-fei decoction ameliorates naïve CD4(+) T cell senescence and attenuates age-related COPD by regulating the C1qbp-cGAS-STING signaling pathway. Phytomedicine. 2025;149:157513. 41270386 10.1016/j.phymed.2025.157513

[162] Qi J, Luo Q, Zhang Q, Wu M, Zhang L, Qin L, et al. Yi-shen-xie-zhuo formula alleviates cisplatin-induced AKI by regulating inflammation and apoptosis via the cGAS/STING pathway. J Ethnopharmacol. 2023;309:116327. 36889420 10.1016/j.jep.2023.116327

[163] Ma Y, Bai B, Liu D, Shi R, Zhou Q. Shenqi Fuzheng injection reduces cisplatin-induced kidney injury via cGAS/STING signaling pathway in breast cancer mice model. Breast Cancer (Dove Med Press). 2024;16:451–69. 39165276 10.2147/BCTT.S475860 PMC11335009

[164] Zhu J, Yuan A, Le Y, Chen X, Guo J, Liu J, et al. Yi-Qi-Jian-Pi-Xiao-Yu formula inhibits cisplatin-induced acute kidney injury through suppressing ferroptosis via STING-NCOA4-mediated ferritinophagy. Phytomedicine. 2024;135:156189. 39515100 10.1016/j.phymed.2024.156189

[165] Zheng M, Hu Z, Wang Y, Wang C, Zhong C, Cui W, et al. Zhen Wu decoction represses renal fibrosis by invigorating tubular NRF2 and TFAM to fuel mitochondrial bioenergetics. Phytomedicine. 2023;108:154495. 36257219 10.1016/j.phymed.2022.154495

[166] Guo Y, Xue T, Meng X, Yan P, Zhang M, Zhang Q, et al. Qihuang Yishen formula attenuates renal tubular injury by modulating inflammation via the cGAS/STING pathway in diabetic kidney disease. Phytomedicine. 2025;148:157399. 41124706 10.1016/j.phymed.2025.157399

[167] Peng Y, Wu S, Xu Y, Ye X, Huang X, Gao L, et al. Huangqi-Danshen decoction alleviates renal fibrosis through targeting SCD1 to modulate cGAS/STING signaling. J Ethnopharmacol. 2025;342:119364. 39832629 10.1016/j.jep.2025.119364

[168] Zhou Y, Wang X, Lyu J, Ruan Q, Zeng X, Li Y, et al. Baitong Decoction ameliorates DSS-induced colitis via modulation of STING and JAK/STAT pathways. ACS Omega. 2025;10(43):51215–27. 41210816 10.1021/acsomega.5c06233 PMC12593019

[169] Li H, Xia C, Song Y, Chen J, Liu Y. Unveiling the mechanisms of American ginseng and achyranthes in treatment of primary Sjogren’s syndrome via mtDNA-cGAS-STING pathway insights from network pharmacology, molecular dynamics, and experimental validation. Front Immunol. 2025;16:1675429. 41112290 10.3389/fimmu.2025.1675429 PMC12531212

[170] Zhu W, Chen L, Li Y, Gu R, Zhang Y, Lu Y, et al. Maidong Dishao Decoction can inhibit the activation of the cGAS-STING signaling pathway of Sjögren’s syndrome by reducing oxidative stress in salivary gland epithelial cells. J Ethnopharmacol. 2026;354:120470. 40882790 10.1016/j.jep.2025.120470

[171] Shi W, Xu G, Gao Y, Yang H, Liu T, Zhao J, et al. Compound Danshen Dripping Pill effectively alleviates cGAS-STING-triggered diseases by disrupting STING-TBK1 interaction. Phytomedicine. 2024;128:155404. 38507852 10.1016/j.phymed.2024.155404

[172] Zhang H, Song X, Ge S, Song W, Wang F, Yin Q, et al. Zixue powder attenuates septic thrombosis via reducing neutrophil extracellular trap through blocking platelet STING activation. J Ethnopharmacol. 2024;331:118337. 38740110 10.1016/j.jep.2024.118337

[173] Feng Y, Yusufu R, Chen T, Guan Z, Zhang L, Li S, et al. Fufang epimedium formula alleviates acute liver injury through dual modulation of PI3K/AKT/Bcl-2 anti-apoptotic signaling pathway and cGAS-STING-IRF3 anti-inflammatory signaling pathway. J Ethnopharmacol. 2025;353(Pt B):120415. 40812556 10.1016/j.jep.2025.120415

[174] Shen AL, Wen ZY, Du YQ, Ding HM, Hu ZQ, Zhou L, et al. Tao Hong Si Wu decoction attenuates myocardial injury in diabetic mice by regulating the cGAS/STING/NLRP3 axis. 3 Biotech. 2025;15(12):413. 41250668 10.1007/s13205-025-04516-x PMC12619888

[175] Chen Y, Wang G, Jiang Y, Qian K, Liu Y, Qiu C, et al. Traditional medicine Kuanxiong Aerosol alleviates atherosclerotic plaque inflammation by mitigating CX3CR1(+) macrophage apoptosis via the cGAS-STING pathway. Phytomedicine. 2025;146:157120. 40753933 10.1016/j.phymed.2025.157120

[176] Zhu L, Liu Z, Liu J, Li Z, Bao Y, Sun X, et al. NCOA4 linked to endothelial cell ferritinophagy and ferroptosis:a key regulator aggravate aortic endothelial inflammation and atherosclerosis. Redox Biol. 2025;79:103465. 39700692 10.1016/j.redox.2024.103465 PMC11729014

[177] Fu G, Li T, Zhao Y, Zhang S, Xue X, Yang Y, et al. Dingzhen pills inhibit neuronal ferroptosis and neuroinflammation by inhibiting the cGAS-STING pathway for Parkinson’s disease mice. Chin Med. 2025;20(1):87. 40524254 10.1186/s13020-025-01135-9 PMC12168260

[178] Luo H, Wang G, Liu T, Guo J, Li Q, Cao Z, et al. Buyang Huanwu Decoction alleviates vascular cognitive impairment by inhibiting neuroinflammation via regulation of the p53/cGAS/STING pathway. J Ethnopharmacol. 2026;362:121319. 41638453 10.1016/j.jep.2026.121319

[179] Yang J, Yuan G, Shuang R, Chen C, Zhang J, Wu T, et al. Zhi-Zi-Hou-Po decoction ameliorates depressive-like behaviors via TFAM-induced mitophagy in neurons to alleviate neuroinflammation. J Ethnopharmacol. 2026;362:121388. 41702466 10.1016/j.jep.2026.121388

[180] Gao Y, Wang H, Ma P, Nie K, Gong M, Su H, et al. Jiao-tai-wan and its bioactive constituent jatrorrhizine exert antidepressant effects via the STING pathway. J Ethnopharmacol. 2026;354:120547. 40915371 10.1016/j.jep.2025.120547

[181] Luo J, Min Y, Zhou L, Xiao F, Zhang X, Sun A, et al. Jiangtang Tiaozhi formula relieves HFD-induced obesity related type 2 diabetes by inhibiting the cGAS-STING pathway. J Cell Mol Med. 2025;29(22):e70882. 41250907 10.1111/jcmm.70882 PMC12623462

[182] Yang L, He T, Chen G, Ou X, Huang B, Yan T, et al. San Huang decoction accelerates diabetic wound healing by restraining STING signaling to attenuate inflammation and enhance angiogenesis. J Ethnopharmacol. 2026;362:121327. 41643877 10.1016/j.jep.2026.121327

[183] Liu L, Zhan X, Wang X, Wen J, He C, Chen X, et al. *Epimedium brevicornu* Maxim. extract activates natural killer cells against hepatocellular carcinoma via the cGAS-STING pathway. Front Pharmacol. 2025;16:1681650. 41357886 10.3389/fphar.2025.1681650 PMC12678249

[184] Liu J, Xiang J, Jin C, Ye L, Wang L, Gao Y, et al. Medicinal plant-derived mtDNA via nanovesicles induces the cGAS-STING pathway to remold tumor-associated macrophages for tumor regression. J Nanobiotechnology. 2023;21(1):78. 36879291 10.1186/s12951-023-01835-0 PMC9990354

[185] Zhai P, Chen Q, Wang X, Ouyang X, Yang M, Dong Y, et al. The combination of Tanshinone IIA and Astragaloside IV attenuates myocardial ischemia-reperfusion injury by inhibiting the STING pathway. Chin Med. 2024;19(1):34. 38419127 10.1186/s13020-024-00908-y PMC10900662

[186] Qiu C, Xia F, Tu Q, Tang H, Liu Y, Liu H, et al. Multimodal lung cancer theranostics via manganese phosphate/quercetin particle. Mol Cancer. 2025;24(1):43. 39905491 10.1186/s12943-025-02242-9 PMC11796208

[187] Gao N, Huang Y, Jing S, Zhang M, Liu E, Qiu L, et al. Environment-responsive dendrobium polysaccharide hydrogel embedding manganese microsphere as a post-operative adjuvant to boost cascaded immune cycle against melanoma. Theranostics. 2024;14(10):3810–26. 38994034 10.7150/thno.94354 PMC11234272

[188] Du S, Chen C, Qu S, Song H, Yang J, Li Y, et al. DNAzyme-assisted nano-herb delivery system for multiple tumor immune activation. Small. 2022;18(45):e2203942. 36156383 10.1002/smll.202203942

[189] Ling Y, Liang X, Yan K, Zeng G, Zhu X, Jiang J, et al. Bimetallic Ca/Zn Nanoagonist Remould the immunosuppressive hepatocellular carcinoma microenvironment following incomplete microwave ablation via pyroptosis and the STING signaling pathway. Adv Sci. 2025;12(23):e2500670. 10.1002/advs.202500670 PMC12199322 40305756

[190] Bai G, Liu H, Li W, Chen S, Wang L, Wang Z, et al. Mitochondria-targeting polymer cLipG/CuET activates the cGAS/STING pathway to enhance cholangiocarcinoma immunotherapy. J Nanobiotechnol. 2025;23(1):631. 10.1186/s12951-025-03689-0 PMC12512424 41068916

[191] Peng H, Chang A, Zhang H, Xu X, Wang W, Zhang K, et al. A novel biomimetic nanoplatform amplifies ferroptosis for specific cGAS-STING pathway activation to enhance hepatocellular carcinoma chemo-immunotherapy. J Nanobiotechnol. 2025;23(1):799. 10.1186/s12951-025-03870-5 PMC12752450 41291739

[192] Xu D, Hu J, Mei J, Zhou J, Wang Z, Zhang X, et al. Nanoadjuvant-triggered STING activation evokes systemic immunotherapy for repetitive implant-related infections. Bioact Mater. 2024;35:82–98. 38283386 10.1016/j.bioactmat.2024.01.020 PMC10818060

[193] Xu A, Yang R, Zhang M, Wang X, Di Y, Jiang B, et al. Macrophage targeted triptolide micelles capable of cGAS-STING pathway inhibition for rheumatoid arthritis treatment. J Drug Target. 2022;30(9):961–72. 35467469 10.1080/1061186X.2022.2070173

[194] Xiao J, Zhou S, Fei F, Long L, Guo C. Pectin-loaded ferulic acid nanoparticles: a potential therapeutic strategy for ulcerative colitis via modulation of the cGAS-STING pathway. Toxicol Appl Pharmacol. 2025;499:117317. 40174805 10.1016/j.taap.2025.117317

[195] Li W, Du Y, Zhang B, Gu D, Zhao X, Chen L, et al. Naringenin-loaded SBMA/GelMA hydrogel: restoring immune balance and promoting angiogenesis via the PPARα/STING pathway in diabetic wounds. Colloids Surf B Biointerfaces. 2025;252:114700. 40233481 10.1016/j.colsurfb.2025.114700

[196] Wang X, Lu Q, Liu X, Zheng Z. Formosanin C inhibits infiltration of MDSCs by activating the STING pathway in non-small cell lung cancer. Int Immunopharmacol. 2026;168(Pt 1):115844. 41242273 10.1016/j.intimp.2025.115844

[197] Jiang ZB, Xu C, Xu P, Huang DH, Kang LP. Lycorine suppresses non-small-cell lung cancer progression through activating STING pathway and stimulating an antitumor immune response. Chem Biol Drug Des. 2024;104(6):e70036. 39707625 10.1111/cbdd.70036

[198] Yan X, Yao C, Fang C, Han M, Gong C, Hu D, et al. Rocaglamide promotes the infiltration and antitumor immunity of NK cells by activating cGAS-STING signaling in non-small cell lung cancer. Int J Biol Sci. 2022;18(2):585–98. 35002511 10.7150/ijbs.65019 PMC8741839

